# Beyond Mimicking Enzymes: NewTAML/Peroxide Abstracts sp^3^ C–H Bonds to Initiate Biotranscendent Water-Purifying Mineralization of Fluoroquinolone Antibiotics

**DOI:** 10.1021/jacs.5c12768

**Published:** 2025-12-23

**Authors:** Xiaowei Ma, Longzhu Q. Shen, Minerva G. Schafer, Karl H. G. Schulz, Roberto R. Gil, Alexander D. Ryabov, Terrence J. Collins

**Affiliations:** Institute for Green Science, Department of Chemistry, 6612 Carnegie Mellon University, 4400 Fifth Avenue, Pittsburgh, Pennsylvania 15213, United States

## Abstract

In neutral aqueous media under ambient temperature, the bis-amido-bis-sulfonamido-ligated [Fe^III^{4-NO_2_C_6_H_3_-1,2-(*N*COCMe_2_ *N*SO_2_)_2_CHMe}­(OH_2_)]^−^ NewTAML catalyst (**2**), activates hydrogen peroxide to mimic and greatly surpass enzymatic activity (biotranscendence) by sweeping fluoroquinolone antibiotics rapidly toward minerals. Stepwise identification of intermediates was paired with mechanistic examination of key steps. For ofloxacin, the **2**/H_2_O_2_ activated catalyst (AC) first attacks ofloxacin’s piperazine unit in two enzyme-mimicking parallel pathways, cleaving secondary (desaturation) and primary (*N*-demethylation) sp^3^ C–H bonds via H atom abstraction to form dehydro- and desmethyl-ofloxacin, respectively; desaturation is a novel expression of TAML peroxidase-like reactivity. Kinetic data obtained with selectively deuterated ofloxacin compounds revealed for desaturation and *N*-demethylation, respectively, kinetic isotope effects (*k*_H_/*k*_D_) of 8.6 and 3.8; Δ*H* ^‡^ and Δ*S* ^‡^ are presented. Dehydro- and desmethyl-ofloxacin are further oxidized through several identified intermediates toward minerals; ion chromatography revealed F^–^, CH_3_COO^–^, HCOO^–^, NO_2_^–^, and NO_3_^–^ as final products. **2**/H_2_O_2_ similarly oxidizes ciprofloxacin and norfloxacin. Desaturation by **2**/H_2_O_2_ of 1-methyl-4-phenylpiperazine delivered a rate that is comprehensibly half that of ofloxacin. DFT calculations provided compelling evidence that the **2**/H_2_O_2_ effective AC is oxidized by two-electrons above the ferric state producing ofloxacin desaturation via sequential hydrogen atom abstraction, single electron transfer and hydrolysis events. Iron­(IV) compounds, once considered plausible as ACs in TAML/peroxide C–H bond activations, cannot oxidize ofloxacin sp^3^ C–H bonds. The temporal toxicity profile of ofloxacin’s degradation intermediates based on *in silico* toxicities highlights that **2**/H_2_O_2_ can serve as a powerful *safe-and-sustainable-by-design* (SSbD) fluoroquinolone detoxifying water treatment.

## Graphical abstract

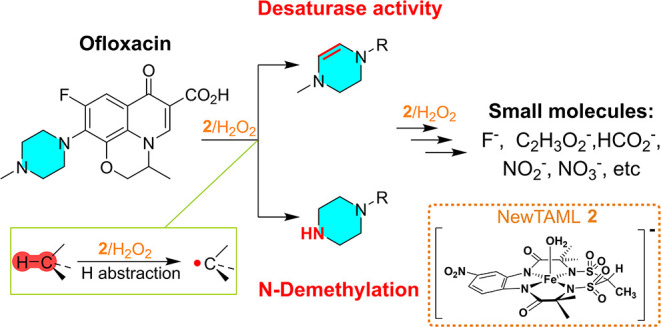

## 1 Introduction

The synthetic virtuosity that chemists have acquired over almost two centuries to create myriad commercially useful chemicals and materials together with the triumphs of organic synthesis in delivering beguilingly complex natural products sum to one of the greatest technical achievements of our civilization. Synthetic compounds are typically key components of the vast array of products and processes upon which our everyday existence rests. Perhaps nowhere is this more obvious than with the antibacterial agents of the pharmaceutical industry among which fluoroquinolones are the most commonly used (and often overused) class.^1–3^ As with chemicals in general, fluoroquinolones have been commercialized on the strength of favorable technical and cost performances, making rapid cures for debilitating and even lethal bacterial infections more widely available at very high benefit/cost ratios from the curative perspective. However, while the high bioactivity of antibiotics is a therapeutic necessity, many are also structurally complex, environmentally persistent, bioaccumulative, and resistant to enzymatic degradation.^4,5^ Long after human and animal excretion (typically into water) has concluded the health-promoting phase of an antibiotic life cycle, released residual drug can continue to impact aquatic life via low concentration adverse effects, all while bacterial resistance is promoted toward rendering useless these precious chemical contributions to human and animal welfare.^3,4,6^

Fluoroquinolones (FQs) provide a notable case in point. Up to 70% of ingested fluoroquinolones are excreted from patients unmetabolized to end up, at least partially, in environmental waters on a global scale.^6,7^ Some fluoroquinolones, for example ofloxacin and ciprofloxacin (Chart 1), are known micropollutants (MPs) that elicit adverse effects at low concentrations in water and are current or past entries in the EU Water Framework Directive Watch List^8^ as one measure of global safety concern.^9^ The antibiotics industry could be lifted onto a safer and more sustainable plane if the downsides that accompany normal antibiotic use could be safely, easily, and cheaply neutralized without limiting the health benefits. This work cements the scientific case for such a profound benefit of NewTAML/peroxide as a foundational achievement within the SSbD framework.^10^

**Chart 1:**
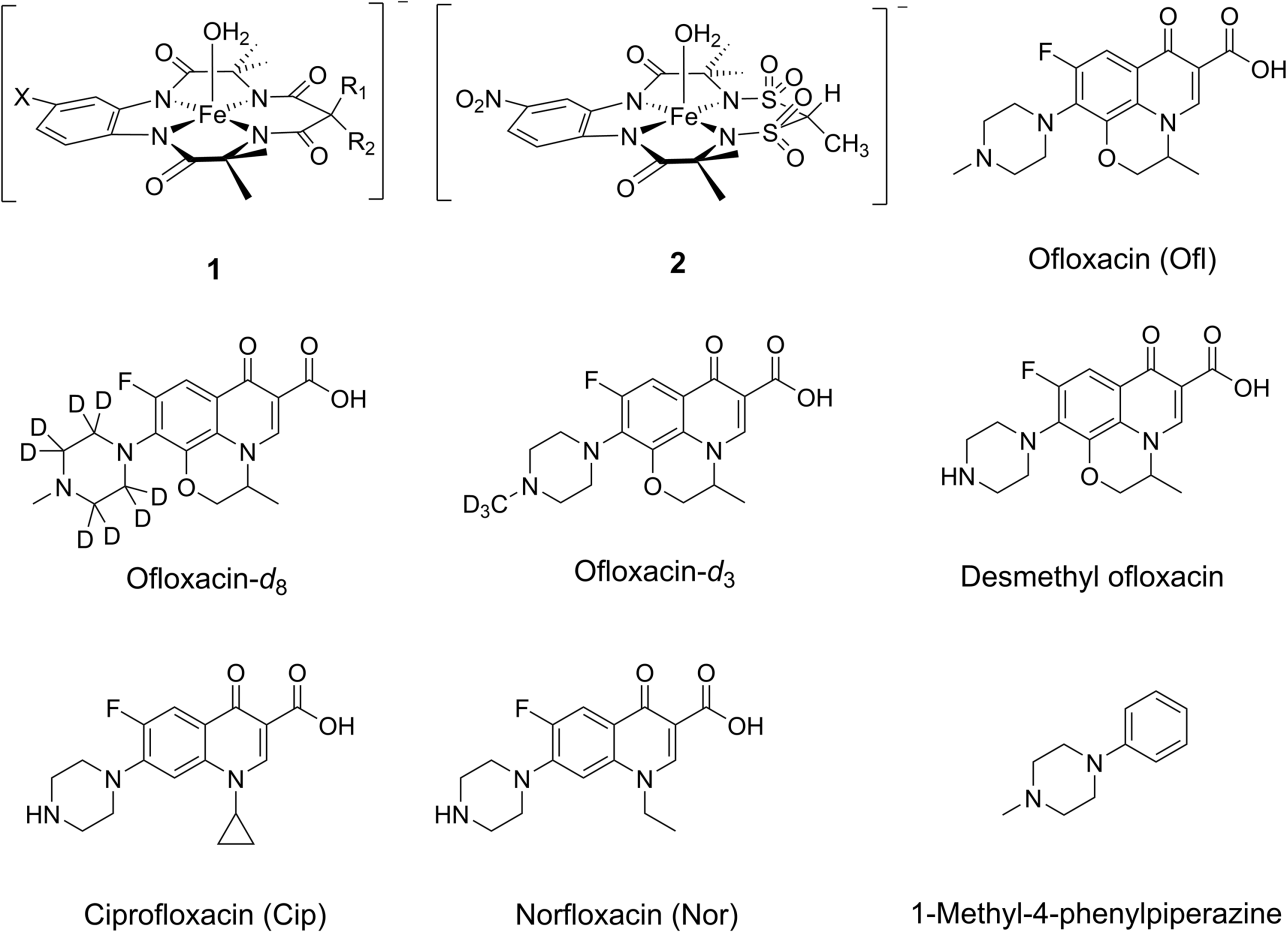
TAML Activators, Fluoroquinolone Antibiotics and 1-Methyl-4-phenylpiperazine Used in This Study (**1a**, X = H, R = Me; **1b**, X = NO_2_, R = F)

TAML complexes are low molecular weight ferric functional replicas of peroxidase enzymes.^11^ The latest generation, NewTAML catalysts such as **2** (Chart 1), function more effectively than earlier TAMLs such as **1** ^12^ in removing MPs from environmental and waste waters giving impressive technical performances under ultradilute catalyst (2.5 to 30 nM) and very dilute hydrogen peroxide (1 to 10 ppm) concentrations, with treatment times ranging from a few minutes to several hours, thereby expanding the powers of this very easy to use approach for oxidatively neutralizing MPs.^13^

To make the many techniques and themes that comprise this study easier to follow, we direct the reader to the following flow diagram (Scheme 1) that identifies where the various components are to be found in the paper. This study establishes that (i) the **2**/H_2_O_2_ degrades fluoroquinolones to small ions including F^–^, CH_3_COO^–^, HCOO^–^, NO_2_^–^ and NO_3_^–^ while achieving near mineralization, (ii) ofloxacin degradation starts via two parallel pathways, desaturation and *N*-demethylation, (iii) **2**/H_2_O_2_ abstraction of sp^3^ C–H bonds on the Ofl piperazine moiety is the rate-limiting step of both pathways that first unfold in enzyme-like desaturation and *N*-demethylation, (iv) **2**/H_2_O_2_ also induces desaturation and *N*-demethylation in the model compound, 1-methyl-4-phenylpiperazine, comprehensibly at half the rate found for ofloxacin, (v) several other intermediates that form after the desaturation and *N*-dealkylation have been identified by HPLC and LC-MS, (vi) ofloxacin-like reactivity was found for other fluoroquinolones indicating that the common piperazine unit is also the most vulnerable component of these targets, (vii) NewTAML **2** outperforms TAMLs of previous generations in fluoroquinolone degradation processes with the reactivity dropping in the representative series as **2** > **1b** ≫ **1a**; (viii) as revealed by DFT, AC compositions are highly reactive toward C–H bond activation when oxidized by two electrons above the ferric resting state, whereas those at the Fe­(IV) state are effectively inert in these reaction channels.

**Scheme 1:**
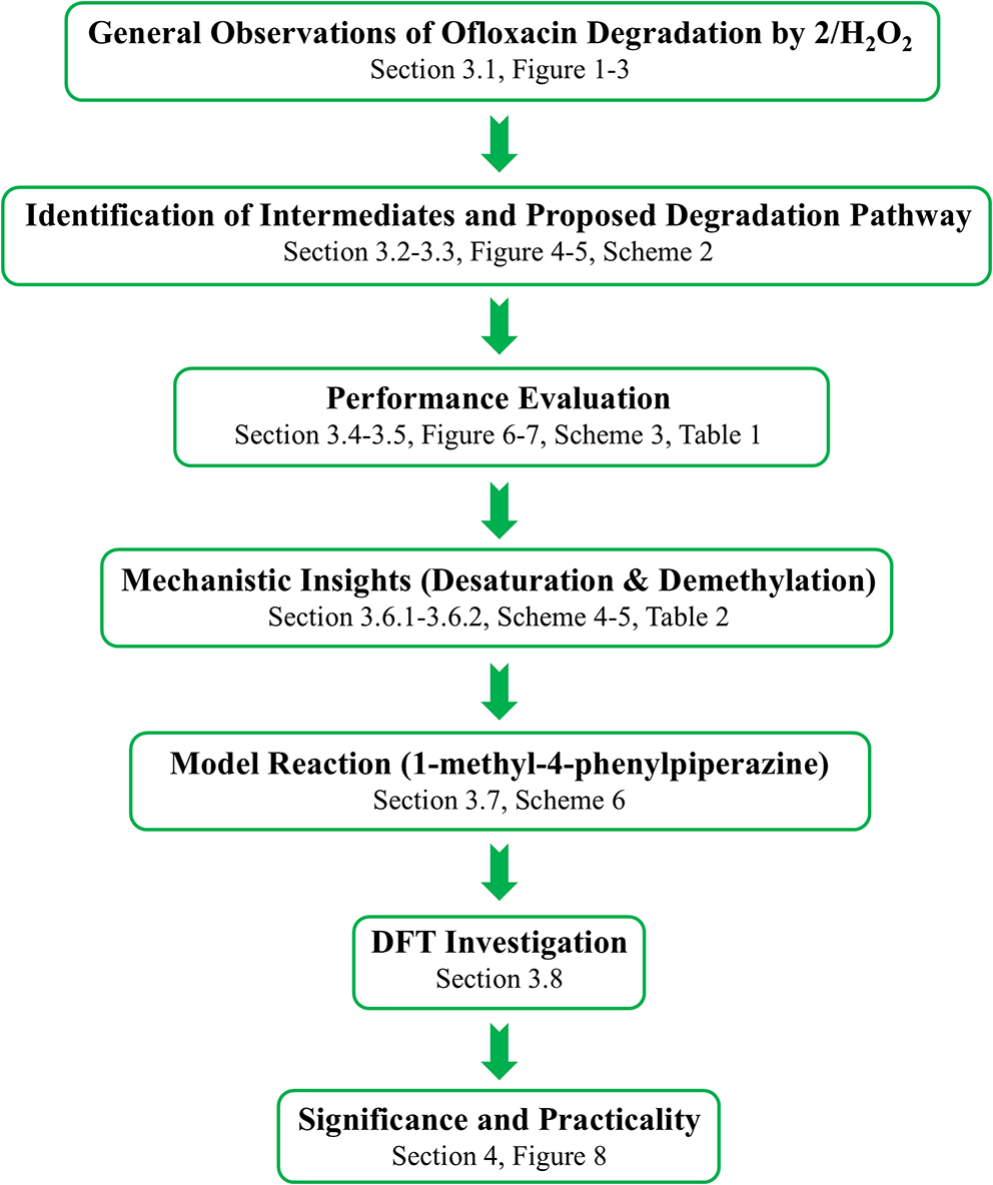
Schematic Illustration of the Overall Research and Interpretation Workflow

## 2 Experimental Section

### 2.1 Materials

All chemicals were analytical grade, and all lab solvents were HPLC grade unless otherwise specified. Ofloxacin, ofloxacin-*d*_3_, ciprofloxacin, norfloxacin, sodium thiosulfate, KH_2_PO_4_ and K_2_HPO_4_ (both ACS reagent grade), hydrogen peroxide (30% w/w), formic acid (LC-MS grade) were all purchased from Sigma-Aldrich. Phosphoric acid (85% w/w), water, methanol, and acetonitrile were obtained from Fisher Scientific. Deuterium oxide was acquired from Cambridge Isotope Laboratories, Inc. Desmethylofloxacin and ofloxacin-*d*_8_ were purchased from Cayman Chemical Company (Ann Arbor, MI). 1-Methyl-4-phenylpiperazine was obtained from AstaTech, lnc. (Bristol, PA). NewTAML **2** was a gift from SUDOC, LLC (Pittsburgh, PA). **1a** and **1b** were obtained over 15 years ago from GreenOx Catalysts, Inc. (now closed). Municipal wastewater was obtained from a plant in Western PA.

### 2.2 Instrumentation

Chromatographic analyses were carried out on a Shimadzu HPLC system [Shimadzu CMB-20A controller, LC-20AB pump, DGU-20A3 degasser, SPD-M20AV diode array detector, RF-20A XS fluorimeter detector, CTO-20A column oven, and SIL-20AC autosampler] with a Phenomenex Kinetex EVO C18 100 Å (100 mm × 4.6 mm, 5 μm) column. Different HPLC methods were used for different fluoroquinolones with 10 μL injection volume; details can be found in Table S1. LC–MS data were obtained using a Thermo Scientific Exactive Plus EMR Orbitrap Mass Spectrometer and Thermo Scientific Vanquish Flex Binary UPLC equipped with a Phenomenex Kinetex EVO C18 100 Å (100 mm × 4.6 mm, 5 μm) column under the positive electrospray ionization mode. The chromatographic methods of LC-MS are shown in Table S2. Ion chromatography analyses were performed using the Thermo Scientific Dionex Ion chromatography system (ICS) 5000+. A Dionex IonPac AS18 IC (4 mm × 250 mm) coupled with an AG18 (4 mm × 50 mm) guard column was used. Analyses were performed under gradient elution with concentration of KOH (mobile phase) started and held at 2.5 mM for 10 min, increased to 30 mM over 20 min, maintained for 10 min, then decreased back to 2.5 mM within 5 min and held for another 5 min. The flow rate was 1.2 mL min^–1^ and the injection volume was 25 μL. NMR data were collected on a Bruker NEO 500 MHz NMR instrument with a Multinuclear Prodigy Cryoprobe. The concentration of hydrogen peroxide stock solution was checked daily with an Agilent 8453 diode array spectrophotometer (ε = 72.8 cm^–1^ M^–1^ at 230 nm).^14^ The pH measurements were acquired with a Fisherbrand Accumet AB15 Basic pH meter calibrated with standard buffer solutions at pHs 4, 7, and 10.

### 2.3 Experimental Details

In a typical degradation process, fluoroquinolone, TAML and hydrogen peroxide stock solutions were sequentially added to phosphate buffer (0.01 M pH 7.0) to start the reaction. Stock solution concentrations were varied giving the different initial conditions noted in each figure. The reaction mixtures were analyzed by UV–vis, HPLC, LC-MS, IC, and NMR. Transformation of 1-methyl-4-phenylpiperazine was conducted the same way and analyzed by HPLC and HR-ESI-MS. For IC and ^19^F NMR analyses of the final mineralization products, the reactions were carried out using the same procedure, with total volumes of 10 and 50 mL, respectively. After 6 h of reaction, the solutions were evaporated and redissolved in 1 mL water or D_2_O for subsequent analyses.

Kinetic studies followed similar procedures with a total reaction volume of 6 mL. After initiating the reaction, at certain time intervals, 1 mL of the solution was withdrawn and quenched with Na_2_S_2_O_3_ at five times the concentration of H_2_O_2_. Concentrations of fluoroquinolones were measured by HPLC. The initial rates were calculated from linear plots of fluoroquinolones concentration vs time prior to the degradation percentage exceeding 10–20%.

### 2.4 DFT Calculations

Computational suite Gaussian 16, rev. C.01 ^15^ was employed to investigate the dehydrogenation mechanism of ofloxacin. 1,4-dimethylpiperazine, with the same reactive moiety of Ofl hosting the dehydrogenation event, was chosen as the model molecule for studying the reaction mechanism. The experimental system can hold up to eight possible combinatorial chemical characters in the dehydrogenation process: four forms of the potential active states (one-electron and two-electron oxidized above Fe­(III) with axial oxo and hydroxo) of **2** and two forms of 1,4-dimethylpiperazine (mono- and doubly protonated). All eight hypothetical scenarios were created and simulated *in silico*. The initial structure of **2** was built *in silico* starting by modifying the known structure of a published analogue.^12^ The initial 3D structure of ofloxacin was taken from PubChem (https://pubchem.ncbi.nlm.nih.gov/compound/Ofloxacin). Density functionals M06L^16^ was chosen in this study based on the benchmarking results against two NewTAML (Chart S1) crystal structures (more details are available in the Table S3). A 6-311+G­(d) basis set was used in the geometry optimization and frequency analysis for the ground and transition states. The optimized structures were fed into the def2QZVPD basis set^17^ for single point calculations. The free energies were constructed by correcting the electronic energies from the triple-ζ basis sets with the quadruple-ζ ones and were set as the default energy presentation in this report unless specified otherwise. The SMD continuum model^18^ was used where appropriate to account for the solvent effect. The spin state of each AC and its reactive complex was set based on the electronic configuration expected from studies of older TAMLs^19,20^ (*s* = 1/2 for 2e-ox and *s* = 1 for 1e-ox), which have been specified in the structural output in the SI. All the reported geometrical configurations were obtained after the default convergence criteria had been met. Transition states were examined and confirmed by the imaginary frequency corresponding to the expected chemistry transferring event.

## 3 Results

### 3.1 General Observations: UV–vis and HPLC Monitoring

Ofloxacin has two strong UV–vis bands at 287 (ε 21,590 M^–1^ cm^–1^) and 332 nm (ε 9550 M^–1^ cm^–1^). Therefore, its **2**/H_2_O_2_ degradation was first examined by this most convenient spectroscopy under conditions approximating common environmental aqueous media; 25 °C and pH 7. The spectral data in Figure 1 indicate that the intensity of both bands decreases as **2**/H_2_O_2_ attacks ofloxacin with a slight blueshift of the 287 nm λ_max_ band. The inset to Figure 1 shows that (i) H_2_O_2_ alone is inactive and (ii) the **2**/H_2_O_2_ process proceeds at a constant rate to completion in ca. 40 min. Then, a slower process is suggested by the spectral changes; the overall absorbance decrease at 287 nm was 37.2% after 90 min. These observations suggested that the ofloxacin oxidation was either incomplete or afforded a less reactive product with a similar UV–vis spectrum to ofloxacin. The former option was ruled out by HPLC.

**Figure 1:**
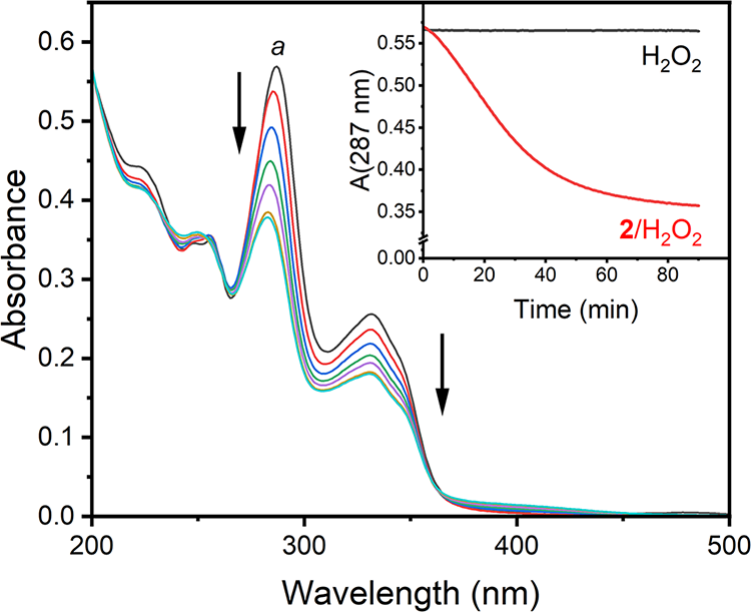
UV–vis monitoring of ofloxacin degradation by **2**/H_2_O_2_. Inset shows the absorbance versus time change at 287 nm with/without **2**. Conditions: [ofloxacin] = 2.5 × 10^–5^ M, [**2**] = 1 × 10^–7^ M, [H_2_O_2_] = 1 × 10^–3^ M, pH 7.0 (0.01 M phosphate), 25 °C. Spectrum *a* was recorded immediately after mixing the reagents; the remaining spectra were run after 10, 20, 30, 50, 70, 90 min.

Following the ofloxacin degradation by **2**/H_2_O_2_, HPLC measurements (Figure 2) established that the ofloxacin peak (retention time/RT 8.4 min) had disappeared after ca. 50 min, which agreed with the absorbance versus time profile in the Inset to Figure 1. New HPLC peaks with RTs 4.7, 6.4, 7.3, and 7.8 min (referred to as OA1, OA7, OA4 and OB0, respectively) became observable as the ofloxacin peak vanished indicating that the apparent incomplete degradation observed by UV–vis (Figure 1) was due to the formation of products with similar UV absorption spectra to ofloxacin. Ofloxacin and OA1 were the two dominating species in solution up to a reaction time of 30 min. The highest concentrations of intermediates OA1 and OB0 were reached after ca. 25 min followed by a gradual decrease in the OA1 and OB0 peaks. This trend can be seen in Figure 3 which presents concentration versus time profiles for ofloxacin and for all intermediates detected by HPLC. It is noteworthy that the concentrations of OA1 and OB0 did not change significantly after 90 min. However, addition of a second aliquot of **2** resulted in their complete elimination, confirming that the reaction stopped at 90 min because of catalyst deactivation. This signals that the concentration of **2** can be easily set such that it undergoes near complete to complete deactivation under functioning catalysis^12,21^ after the targeted MP is completely destroyed. This controllable catalyst lifetime behavior enables precautionary performances that approach the optimal result because water can be completely purged of antibiotic contamination with very little release to the environment of the catalyst that makes it possible, thereby delivering measurable advantages for the health, environmental and fairness performances of TAML systems.

**Figure 2:**
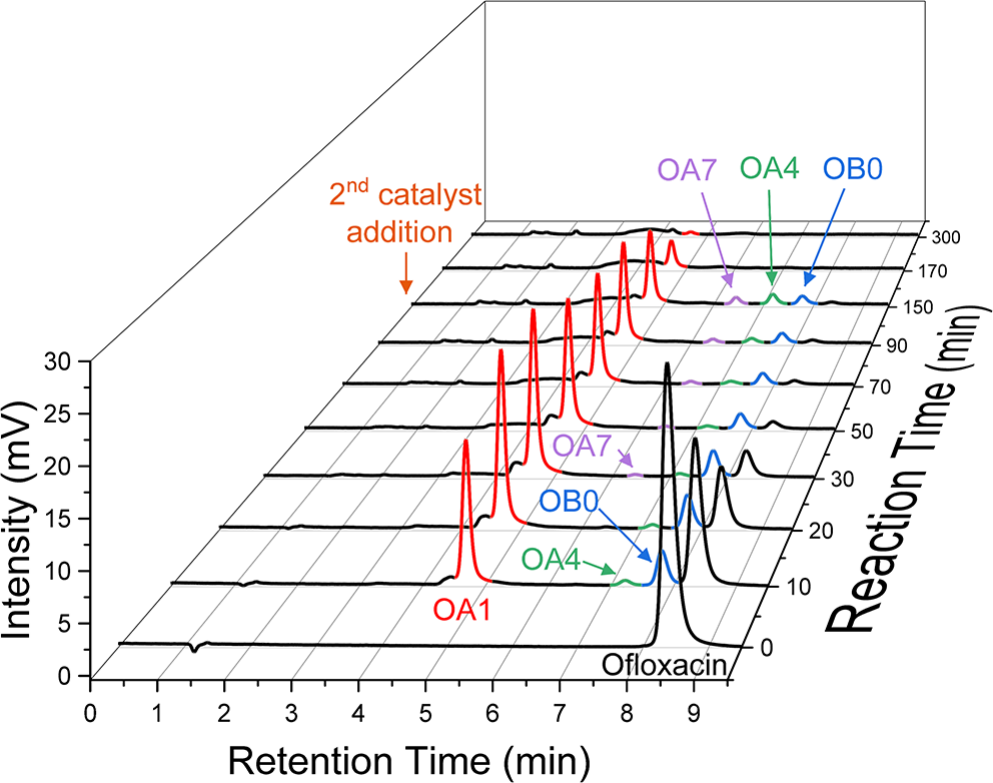
HPLC graphs of the **2**/H_2_O_2_ ofloxacin degradation at different reaction times. An aliquot of **2** was added at 0 and 150 min. Conditions: [ofloxacin] = 3 × 10^–5^ M, [**2**] = 1 × 10^–7^ M (each addition), [H_2_O_2_] = 1 × 10^–3^ M, pH 7.0 (0.01 M phosphate), 25 °C.

**Figure 3:**
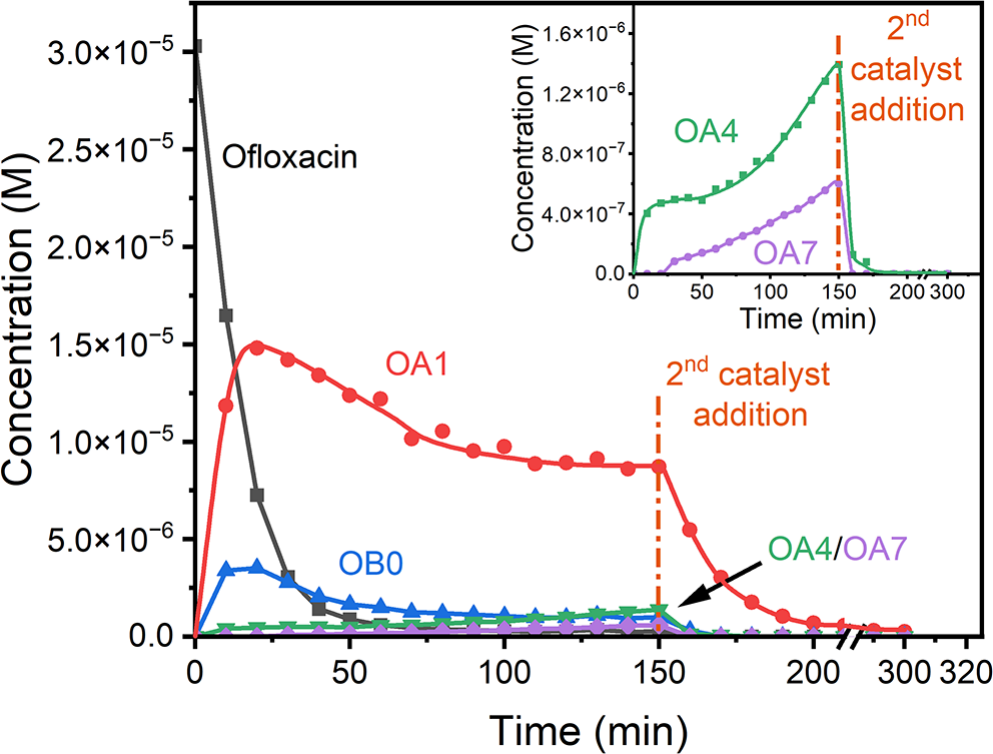
HPLC study of the concentration versus time profiles for ofloxacin and its fragments under **2**/H_2_O_2_ treatment. Inset: zoomed-in profiles for OA4 and OA7. Conditions: [ofloxacin] = 3 × 10^–5^ M, [**2**] = 1 × 10^–7^ M, [H_2_O_2_] = 1 × 10^–3^ M pH 7.0 (0.01 M phosphate), 25 °C. The profiles for OA1, OA4 and OA7 were constructed using averaged calibration parameters measured for ofloxacin and OB0 which are commercially available.

At the same time the concentration of OA4 did not level off after 90 min indicating that it may be produced also without involvement of **2**. The [OA4] versus time profile in the Inset to Figure 3 suggests that the initial rapid growth in the OA4 concentration results from a **2**-catalyzed process, but that its continued slower formation after **2** is inactivated may be the result of a different mechanism. In any case, the second aliquot of **2** rapidly destroys all detected intermediates, other than a trace of OA1 (Figure 3), the slowest degrading intermediate. Notably, **2**/H_2_O_2_ degradation of OA1 is slower than that of ofloxacin.

The concentration profiles for OA1 and OB0 in Figure 3 suggest that both intermediates are produced directly from ofloxacin in a parallel fashion via pathways we have labeled A and B, respectively. This is supported by the mass balance variations in the early stages of the degradation because the total mass of ofloxacin, OA1 and OB0 is close to 100% (104% and 85% after 10 and 20 min, respectively, with respect to the amount of ofloxacin introduced).

Intermediate OA7 was detectable after 30 min. No intermediates other than OA1 and OB0 were detected when the initial concentration of **2** was doubled compared to the 1 × 10^–7^ M of the Figures 2 and 3 experiments (see Figure S1, Supporting Information). Under such conditions, OB0 was undetectable after 40 min while a tiny amount of OA1 was observed after 150 min.

### 3.2 Identification of Intermediates

#### 3.2.1 Mass Spectrometry and Nuclear Magnetic Resonance

Samples with retention times corresponding to intermediates OA1, OA4, OA7 and OB0 were separated by HPLC and investigated by direct-inject high-resolution electrospray ionization mass spectrometry (HR-ESI-MS) in the positive mode. A *m*/*z* of 360.1342 [M + H]^+^ was obtained for OA1 (Figure S2), 2 Da lighter than ofloxacin suggesting dehydrogenation. ^1^H and ^13^C NMR spectroscopy were used to identify the dehydrogenation site. The ofloxacin (5 × 10^–3^ M) oxidation was run in 1 mL of unbuffered D_2_O for 30 min in the presence of 2.5 × 10^–5^ M **2** and 0.01 M H_2_O_2_, i.e., with 2 equiv of peroxide vs ofloxacin. OA1 alone was observed in the mixture by HPLC and the other intermediates were practically undetectable (Inset to Figure S3, top right). The reaction mixture was transferred to an NMR tube; ^1^H and ^13^C NMR spectra were recorded and compared to those of ofloxacin (Figures 4 and S3, respectively). This shows that under the right conditions TAML/peroxide can deliver high selectivity oxidative organic transformations that are of potential use in synthetic chemistry.

**Figure 4:**
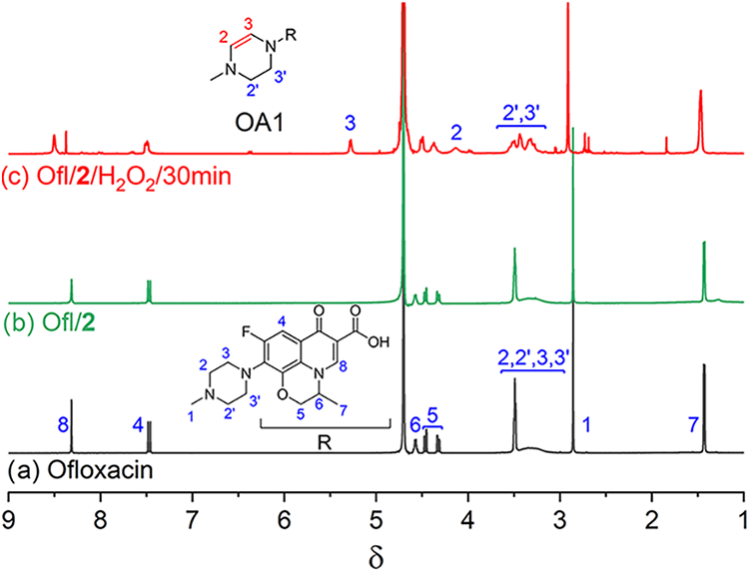
^1^H NMR spectra of (a) ofloxacin, (b) ofloxacin + **2**, and (c) reaction mixture after 30 min. Conditions: [ofloxacin] = 5 × 10^–3^ M, [**2**] = 2.5 × 10^–5^ M, [H_2_O_2_] = 1 × 10^–2^ M, D_2_O, 25 °C.

The ^1^H NMR spectra of (i) ofloxacin, (ii) the ofloxacin/**2** mixture and, (iii) the product of ofloxacin oxidation are presented in Figure 4. The complex spectrum of ofloxacin has been extensively discussed in the literature^22–25^ and therefore the assignments made were based both on our data and the conclusions of other researchers. Iron­(III) TAML activators, including **2**, are paramagnetic species and therefore a line broadening may occur if **2** interacts with organic matter in solution.^26^ This is likely why the ofloxacin resonances for hydrogens 4–8 are broader in Figure 4b than in Figure 4a. The existence of the effect, which is minimal, is supported by DFT calculations (see Section 3.5). The 2,2′,3,3′ hydrogens of the piperazine ring appear as a distinctive pattern at δ 3.55–3.05 because of the ring flip^23^ which changes slightly in the presence of **2**. This group of signals underwent major change when subjected to **2**/H_2_O_2_ (Figure 4c) identifying the piperazine ring as the site of dehydrogenation. The resonances at δ 3.55–3.05 acquired the features of two broadened overlapping triplets of lower integral intensity while two new broad resonances appeared at δ 4.14 and 5.29, i.e., significantly downfield compared to the parent signals at δ 3.55–3.05. This is consistent with the assumption that the postulated dehydrogenation or desaturation involves the piperazine ring of ofloxacin as in eq 1 and provides convincing evidence that the structure of the most abundant intermediate OA1 is correctly shown in Figure 4.
1

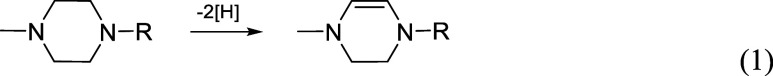




The ^13^C NMR spectrum of the sample as in Figure 4c (Figure S3) was also consistent with dehydrogenation as in eq 1, the key evidence being resonances attributable to two alkenyl carbons at δ 77.6 and 90.6 that were absent in the spectrum of ofloxacin.

Mass-spectral analysis of intermediate OB0 revealed that its mass (*m*/*z* of 348.1358 [M + H]^+^, Figure S4) is 14 Da lower than that of ofloxacin suggesting demethylation of the piperazine ring. This involves a type of C–N bond cleavage that has been previously established for TAML activator treatment of amines (eq 2).^27,28^


2

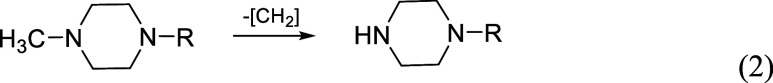




The product of ofloxacin demethylation, desmethylofloxacin, is commercially available. It has a similar HPLC retention time as OB0 (Figure S5) supporting that OB0 is desmethylofloxacin (Scheme 2).

**Scheme 2:**
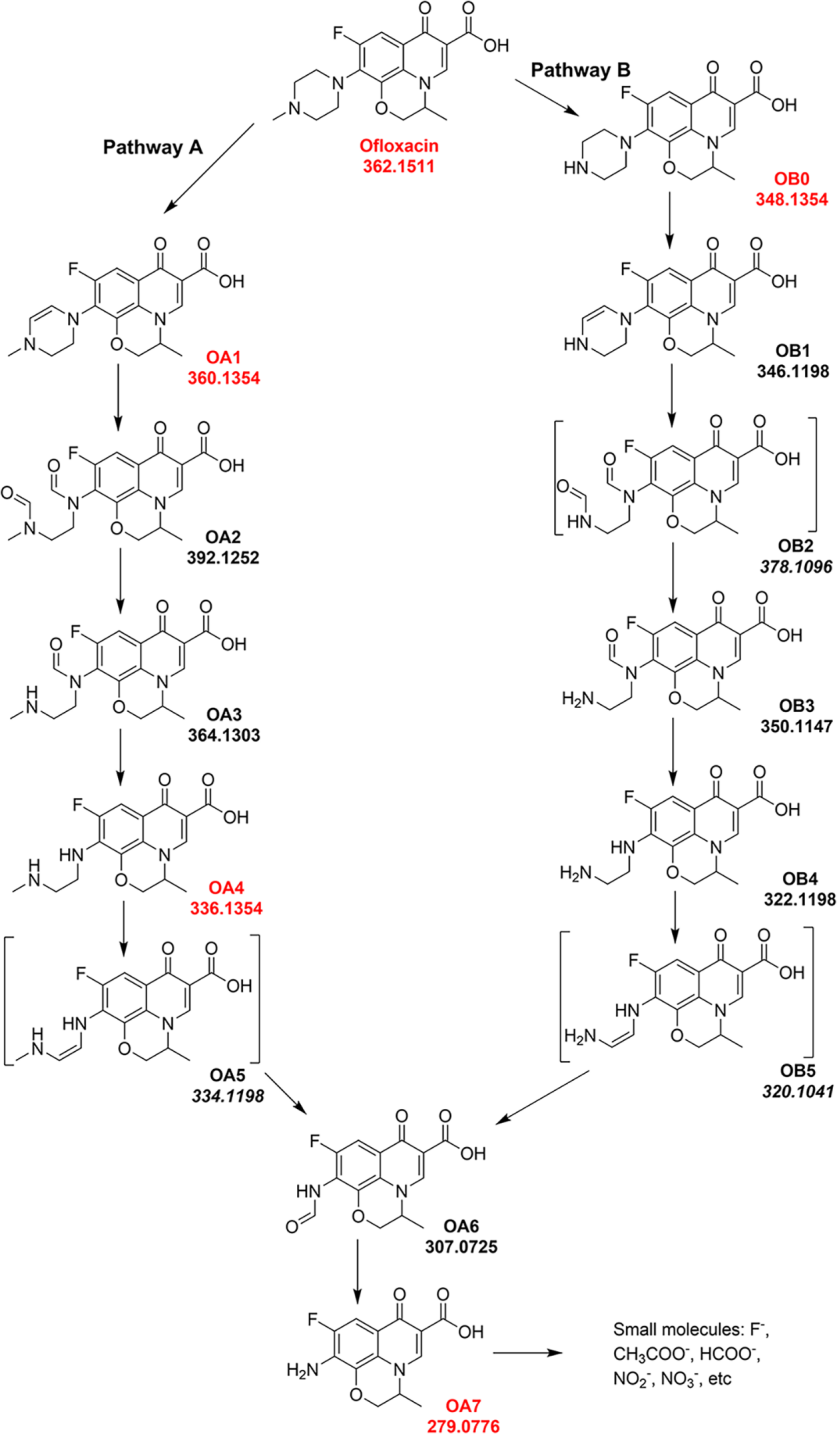
Proposed Degradation Pathways of Ofloxacin by **2**/H_2_O_2_ at pH 7^a^

a Identifiers of degradation intermediates detected by HPLC and LC-MS are in red and black, respectively. Postulated structures are in square brackets. The mass under each compound identifier is the exact mass of [M + H]^+^; the exact mass of each postulated compound is italicized. The two pathways could cross between each other at various junctures.

The *m*/*z* of OA4 measured by HR-ESI-MS (336.1341 [M + H]^+^, Figure S6) is 26 Da lighter than ofloxacin consistent with the loss of two carbons and two hydrogens and with the structure shown in Scheme 2. In turn, OA7 (*m*/*z* 279.0766 [M + H]^+^, Figure S7) is 83 Da lighter than ofloxacin, the difference being the piperazine unit, and therefore OA7 might be considered as “depiperazinated” ofloxacin (Scheme 2). Both OA4 and OA7 have been previously suggested as intermediates during the photodegradation of ofloxacin.^29–31^

### 3.3 Extended Series of Intermediates and Final Products

That the early phase of the ofloxacin degradation by **2**/H_2_O_2_ proceeds by parallel Pathways A and B is thus supported by a convincing weight of evidence including the structural assignments of OA1, OA4, OA7, and OB0, plus the results of other workers,^29–31^ to provide a reliable foundation for the sequence of intermediates of Scheme 2.

Additional information was collected by LC-MS. A solution containing ofloxacin, H_2_O_2_ and **2** (10^–4^, 10^–3^ and 5 × 10^–7^ M, respectively; pH 7.0, 25 °C) was kept for 30 min and then injected to the LC-MS system in the positive mode. The data obtained were screened for the intermediates of Scheme 2. Those with significant [M + H]^+^ signals with measured masses in high agreement with the theoretical values are shown in black (HR-ESI-MS spectra are in Figures S8 and S9). Those with less evident [M + H]^+^ signals are in square brackets in Scheme 2.

Pathway A starts with the desaturation of the piperazine ring of ofloxacin to give OA1. Then, the C=C bond of OA1 is oxidatively cleaved to produce OA2. Oxidative cleavage of alkene to aldehyde using other iron-based catalysts including horseradish peroxidase has been extensively investigated by other workers.^32–35^ Intermediate OA2 then loses two formyl groups to afford first OA3 and then OA4. A similar transformation of OA2 to OA4 was reported by Yu et al. under photocatalysis.^36^ The remaining part of the piperazine unit of OA4 can be reasonably suggested to undergo dehydrogenation giving OA5 followed by oxidative C=C bond cleavage to afford OA6, and then deformylation to OA7 which is finally oxidized into small fragments: OA7 is rapidly destroyed upon addition of the second aliquot of **2** (Figure 2).

Pathway B starts with the formation of OB0 via TAML-catalyzed *N*-demethylation.^27,28^ Then, its demethylated piperazine ring may undergo the same sequence of events as observed for OA1 including the desaturation, oxidative cleavage of the C=C bond, etc., the final intermediate being OA7 as in pathway A.

The nature of the OA7 degradation products was explored by ion chromatography^37^ and the observed peaks were identified by comparison with standards (Figure 5). The detection of fluoride, acetate, formate, nitrite and nitrate (RTs 5.95, 6.63, 7.75, 16.20, and 21.06 min, respectively) is indicative of deep mineralization of ofloxacin. The likely source of chloride (RT 12.82 min) is the starting material target, ofloxacin hydrochloride, and of sulfate (RT 23.10 min) is **2**, which degrades under functioning catalytic conditions as a significant advantage for health, environmental and fairness performances. Quantitative IC analysis showed that fluoride release reached 84.80% of the total fluorine in ofloxacin. Nitrite and nitrate accounted for 4.73 and 62.45% of the nitrogen, while the remaining nitrogen might exist as small nitrogen-containing organic molecules or ammonium. Acetate and formate contributed 1.40 and 8.56% of the carbon (Figure S10). ^19^F NMR was used to further investigate the fluorine release process. As shown in Figure S11, after mineralization by **2**/H_2_O_2_, the peak of ofloxacin at δ −122.50 had disappeared, while a new peak (δ −122.26) corresponding to fluoride had appeared. In addition, two small peaks assigned to alkyl C–F bonds were observed at δ −70 to −75, with no aryl C–F signals detected, indicating that dearomatization of the fluorine bearing ring in the starting ofloxacin had occurred. Quantification of the three peaks using trifluoroacetic acid (TFA) as an internal standard (Figure S12) showed that the fluoride signal accounted for 89.47% of the fluorine in ofloxacin, in good agreement with the IC results. The two alkyl C–F peaks represented 4.45 and 8.19%, respectively, giving a total of 102.11%, which is in good agreement with the mass balance and confirms the absence of other fluorine species. These confirm that **2**/H_2_O_2_ is capable of sweeping ofloxacin toward a combination of near minerals and minerals, a fact that holds broad significance for the treatment of fluoroquinolone contaminated waters where **2**/H_2_O_2_ delivers a remarkably simple to use, high performance process;^13^ mineralization can be considered to be the ultimate objective for removing all vestiges of toxicity.

**Figure 5:**
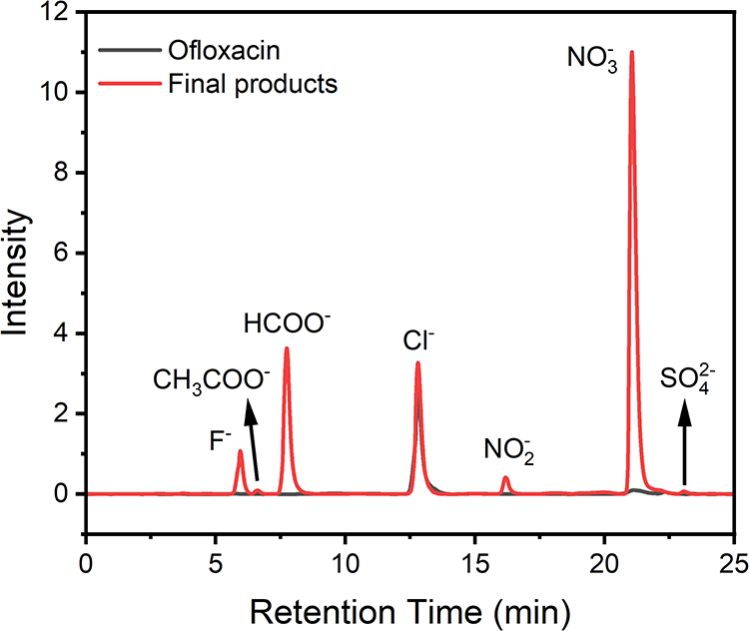
Ion chromatograms of ofloxacin (black) and the final products after **2**/H_2_O_2_ treatment (red). Initial conditions: [ofloxacin] = 2 × 10^–5^ M, [**2**] = 1 × 10^–7^ M (added at 0 and 3h), [H_2_O_2_] = 2 × 10^–3^ M, pH 7.0 (0.01 M phosphate), 25 °C, concentrated 10-fold after reaction.

### 3.4 Other TAMLs and Other Fluoroquinolones

Two other TAML catalysts, **1a** and **1b**, were tested for reactivity comparisons with **2** (Figure 6a). The effective catalytic activity decreases in the series **2** > **1b** ≫ **1a**, i.e., **2** is the most active catalyst and was designed both in pursuit of this high reactivity and to overcome amido ligand perhydrolysis as a catalyst deactivation pathway.^12^ This was further substantiated by measuring the initial rates of ofloxacin degradation by H_2_O_2_ in the presence of **1a**, **b** and **2** (Figure 6b). The rates of ofloxacin degradation were found to be directly proportional to the TAML concentrations in all three cases, the slopes being 49 and 16 times higher for **2** and **1b**, respectively, compared to the prototype TAML catalyst, **1a**.^38^

**Figure 6:**
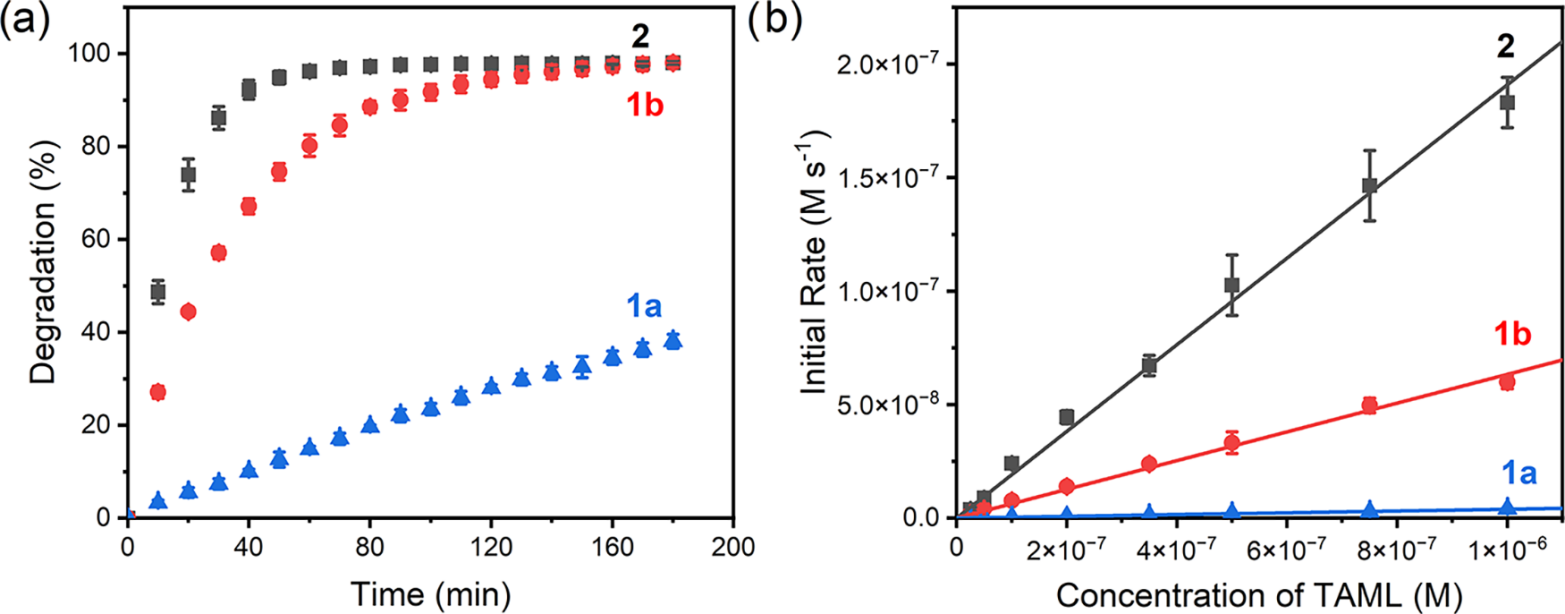
(a) Ofloxacin degradation by H_2_O_2_/TAMLs (**1a**, **b** and **2**). (b) Initial rates of the ofloxacin degradation by H_2_O_2_ versus TAML concentrations. Conditions: [ofloxacin] = 3 × 10^–5^ M, [TAML] = 1 × 10^–7^ M, [H_2_O_2_] = 1 × 10^–3^ M, pH 7.0 (0.01 M phosphate), 25 °C.

The degradations of ciprofloxacin, norfloxacin and desmethylofloxacin (Chart 1) by **2**/H_2_O_2_ were also examined (Figure 7). The **2**/H_2_O_2_ process degraded ciprofloxacin and norfloxacin with similar rates though slightly slower than ofloxacin and desmethylofloxacin reflecting structural differences in the quinolinone ring systems. Notably, 98% of ofloxacin and desmethylofloxacin were degraded after 90 min and 100% degradation of ciprofloxacin and norfloxacin was confirmed.

**Figure 7:**
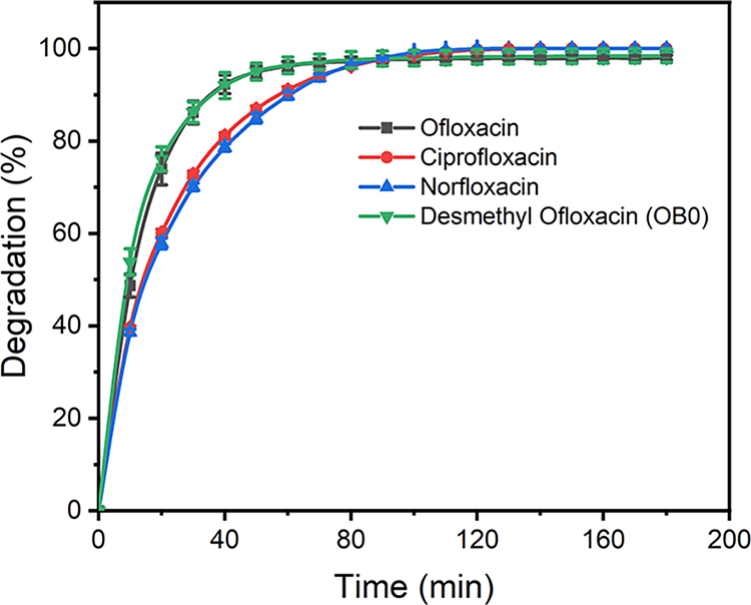
Degradation of fluoroquinolones by **2**/H_2_O_2_. Conditions: [fluoroquinolone] = 3 × 10^–5^ M, [**2**] = 1 × 10^–7^ M, [H_2_O_2_] = 1 × 10^–3^ M, pH 7.0 (0.01 M phosphate), 25 °C.

### 3.5 Detailed Kinetic Information

The TAML/peroxide oxidative degradations of a multitude of substrates were studied kinetically allowing key steps of the stoichiometric mechanism to be established reliably as depicted in Scheme 3.^26^

**Scheme 3:**
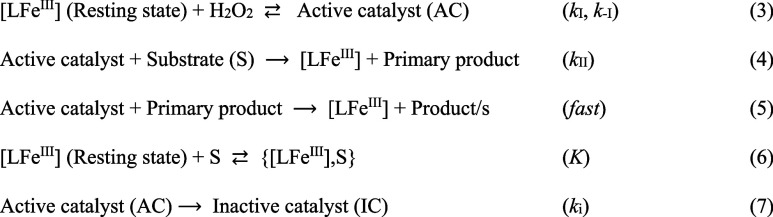
Series of Characterized Steps of the Stoichiometric Mechanism of TAML-Catalyzed Peroxide Oxidation of Targeted Substrates (S) in Aqueous Media

In the early phase of the TAML/peroxide catalytic cycle, the iron­(III)-TAML catalyst reacts first with peroxide to afford the active catalyst (AC, step 3) followed by conversion of S to a primary product (step 4) which may be further oxidized (step 5)—additional degradation steps characteristically occur in later phases as typified by this study. The S may bind reversibly to the resting state (step 6). This binding in clear cases has to date been found to cause inhibition that varies with the nature of the substrate and manifests in a reduction in *k*_I_ because the adducts {[LFe^III^],S} so far have been found to be less reactive toward H_2_O_2_ than [LFe^III^]. DFT calculations presented herein provide support that when S is ofloxacin, the {[LFe^III^],S} adduct (Figure S13) is unusually stable with a free energy of formation of −12 kcal mol^–1^. Finally, AC may undergo irreversible inactivation (step 7). The second-order rate constants *k*_I_ and *k*_II_ (*k*_–I_ is assumed to be negligible) provide essential insight into TAML catalytic cycles, both fundamentally for each TAML and comparatively between TAMLs, and are calculated herein from the hyperbolic dependencies of the initial rates of substrate S depletion as functions of either H_2_O_2_ or *S* concentrations (steps 3–5 predict eq 3). The determination of *k*_i_ values requires experiments featuring the values of *S*_0_ and S_∞_ (*S* concentration at times 0 and ∞, respectively) under conditions where conversion of *S* is incomplete at the point of termination of catalysis, eq 4.^26,39^

8 $$-\frac{d\left[S\right]}{dt}=\frac{k_{I}k_{\mathrm{II}}\left[H_{2}O_{2}\right]\left[S\right]}{k_{I}\left[H_{2}O_{2}\right]+k_{\mathrm{II}}\left[S\right]}\left[{\mathrm{Fe}}_{t}^{\mathrm{III}}\right]$$

9 $$\ln\frac{S_{0}}{S_{\infty }}=\frac{k_{\mathrm{II}}}{k_{i}}\left[{\mathrm{Fe}}_{t}^{\mathrm{III}}\right]$$

The extensive data in Figures S14–S18 regarding the oxidation of fluoroquinolone antibiotics by H_2_O_2_ in the presence of **1**–**2** (Chart 1) are consistent with the mechanism in Scheme 3. The calculated rate constants *k*_I_, *k*_II_ and *k*_i_ are collected in Table 1 and allow for discussion of the deeper implications.

**Table 1:** Rate Constants *k*_I_, *k*_II_ and *k*_i_ for TAML/H_2_O_2_ Degradation of Fluoroquinolones (25 °C) and the Second-Order Rate Constants for Their Ozone Degradations, *k*_O_3__ (20–21 °C) all at pH 7

substrate	TAML	*k*_I_ (M^–1^ s^–1^)	10^–3^ × *k*_II_ (M^–1^ s^–1^)	10^3^ × *k*_i_ (s^–1^)	10^–3^ × *k*_O_3__ (M^–1^ s^–1^)
ofloxacin	**2**	310 ± 10	170 ± 10	2.9 ± 0.2	2.6^a^
	**1b**	150 ± 10	41 ± 2		
	**1a**	10 ± 1	3.4 ± 0.2		
ciprofloxacin	**2**	190 ± 10	150 ± 10	1.4 ± 0.1	19^b^
	**1b**	95 ± 4	30 ± 1		
	**1a**	5.1 ± 0.1	2.8 ± 0.1		
norfloxacin	**2**	180 ± 10	160 ± 10	1.3 ± 0.1	25^c^
	**1b**	92 ± 3	28 ± 1		
	**1a**	5.5 ± 0.2	2.9 ± 0.1		
desmethyl-ofloxacin	**2**	320 ± 10	150 ± 10		
1-methyl-4-phenylpiperazine	**2**	220 ± 10	77 ± 5		

a From ref 40.

b From ref 41.

c From ref 42.

The reactivity comparison series of TAMLs toward H_2_O_2_ (in terms of *k*_I_) is (**2** > **1b** ≫ **1a**). The same series holds for *k*_II_, indicating that **2** exhibits the best technical performance at neutral pH. For different fluoroquinolones, their similar *k*_II_ values indicate that they might have the same initial reactive sites and mechanism, whereas the variation in *k*_I_ arises in part from differences in the influences of substrate inhibition processes in the activation step. The rate constants *k*_II_ for **2** are noticeably higher than the second-order rate constants *k*_O_3__ for the ozonation of fluoroquinolones where ozone is arguably the most adopted oxidation technology for deliberately destroying fluoroquinolones in municipal wastewaters.

### 3.6 Mechanistic Insights into the Cleavage of sp^3^ C–H and C–N Bonds of Fluoroquinolones by Active TAML Species

#### 3.6.1 Desaturase Activity of TAML Activators

Activation of sp^3^ C–H bonds by transition metal complexes continues to attract the attention of researchers^43–47^ which arguably began in 1969 with publication of the pioneering study of Shilov and co-workers.^48^ Though the high expectations associated with the quest for efficient conversions of hydrocarbons have faded somewhat because of the difficulties of finding systems that exhibit useful selectivities and can scale easily, significant interest remains with numerous reports of selective C–H bonds transformations of organic compounds in the presence of metal complexes^44,46,47^ and metal-containing enzymes.^43^ The TAML-catalyzed oxidative degradation of fluoroquinolone antibiotics by H_2_O_2_ is part of this evolving enterprise. For each of the three TAML activators in Table 1, the rate constants *k*_II_ are identical within experimental error for all three fluoroquinolones suggesting that the oxidized iron species AC attack in a slow step at a common fluoroquinolone site which turns out to be the C–H bond-rich piperazine ring.

As shown in Scheme 2, the piperazine ring of ofloxacin, which alone bears a methyl group on nitrogen, degrades via two parallel pathways, affording the characterized intermediates OA1 and OB0, respectively. Pathway A is particularly noteworthy because it shows that TAML activators, in addition to their known peroxidase and catalase activities,^49^ are capable of performing mechanistically challenging desaturase activity that have hitherto been the province of iron-containing oxidizing biocatalysts^50–52^ including α-ketoglutarate-dependent enzymes.^50^ The rate constants for ofloxacin in Table 1 were evaluated by measuring the initial rates of disappearance of ofloxacin and hence each *k*_II_ is an effective value consisting of two terms, viz. *k*_II_^A^ and *k*_II_^B^ (eq 5), that quantitatively characterizes Pathway A and B, respectively and collectively; ciprofloxacin and norfloxacin have no piperazine *N*-CH_3_ group.

10 $$k_{\mathrm{II}}=k_{\mathrm{II}}^{A}+k_{\mathrm{II}}^{B}$$

The rate constants *k*_II_^B^ have been obtained directly for ofloxacin by measuring the initial rates of formation of OB0 using the HPLC technique. In this case

11 $$\frac{d\left[\mathrm{OB}0\right]}{dt}=\frac{k_{I}k_{\mathrm{II}}^{B}\left[H_{2}O_{2}\right]\left[S\right]}{k_{I}\left[H_{2}O_{2}\right]+\left(k_{\mathrm{II}}^{A}+k_{\mathrm{II}}^{B}\right)\left[S\right]}\left[{\mathrm{Fe}}_{t}^{\mathrm{III}}\right]$$

­(*k*_–I_ assumed to be negligible) because the two steps 12 and 13 should be considered instead of the single step 4.

12 $$\mathrm{active}\;\mathrm{catalyst}+\mathrm{Ofl}\to \left[{\mathrm{LFe}}^{\mathrm{III}}\right]+\mathrm{OA}1\left(k_{\mathrm{II}}^{A}\right)$$

13 $$\mathrm{active}\;\mathrm{catalyst}+\mathrm{Ofl}\to \left[{\mathrm{LFe}}^{\mathrm{III}}\right]+\mathrm{OB}0\left(k_{\mathrm{II}}^{B}\right)$$

The value of *k*_II_^B^ is extractable from the slope of the double inverse of eq 6 (Figures S19–S21); *k*_II_^A^ is therefore the difference between *k*_II_ and *k*_II_^B^ (eq 5). The thus-calculated rate constants *k*_II_^A^ and *k*_II_^B^ are summarized in Table 2 to provide independent kinetic information about the relative rates of desaturase and demethylating activities of TAMLs, respectively.

**Table 2:** Rate Constants *k*_II_^A^ and *k*_II_^B^ for the TAML-Catalyzed Degradation of Ofloxacin and Its *d*_8_ and *d*_3_ Derivatives (Chart 1) by H_2_O_2_ at pH 7 and 25 °C

substrate	TAML	10^–3^ × *k*_II_^A^ (M^–1^ s^–1^)	10^–3^ × *k*_II_^B^ (M^–1^ s^–1^)	*k*_II_^A^/*k*_II_^B^
ofloxacin	**2**	146 ± 15	24 ± 1	6.08
	**1b**	24 ± 3	17 ± 1	1.41
	**1a**	2.9 ± 0.3	0.47 ± 0.02	6.23
ofloxacin-*d*_8_	**2**	17 ± 1	31 ± 1	0.55
ofloxacin-*d*_3_	**2**	164 ± 17	6.3 ± 0.3	26.0

The desaturase activity of TAML **2** is significant where one can conceive of two reaction channels where the amount of each route followed should depend upon the ratio of the unprotonated versus protonated methylated piperazine nitrogen atom at pH 7 and the relative rates for the two abstraction processes (Scheme 4 shows one of these, viz., C–H abstraction associated with *k*_II_^A^ where similar steps are envisioned for the *k*_II_^B^ pathway, and S1). We will now lead into these dual processes and explain our thinking on what controls the contribution of each toward the end of in this section. The second order rate constant *k*_II_^A^ for **2** equals 1.5 × 10^5^ M^–1^ s^–1^ under ambient conditions. The related activity of **1b** is 6.1 times lower though both TAMLs possess close reactivity to Orange II dye.^12,53,54^ It seems that the relative activity of tetra-amido and bis-amido bis-sulfonamido TAMLs **1b** and **2**, respectively, may be governed not only by the properties of the individual catalysts, but also by both the nature of the substrate and/or the pathway.

**Scheme 4:**
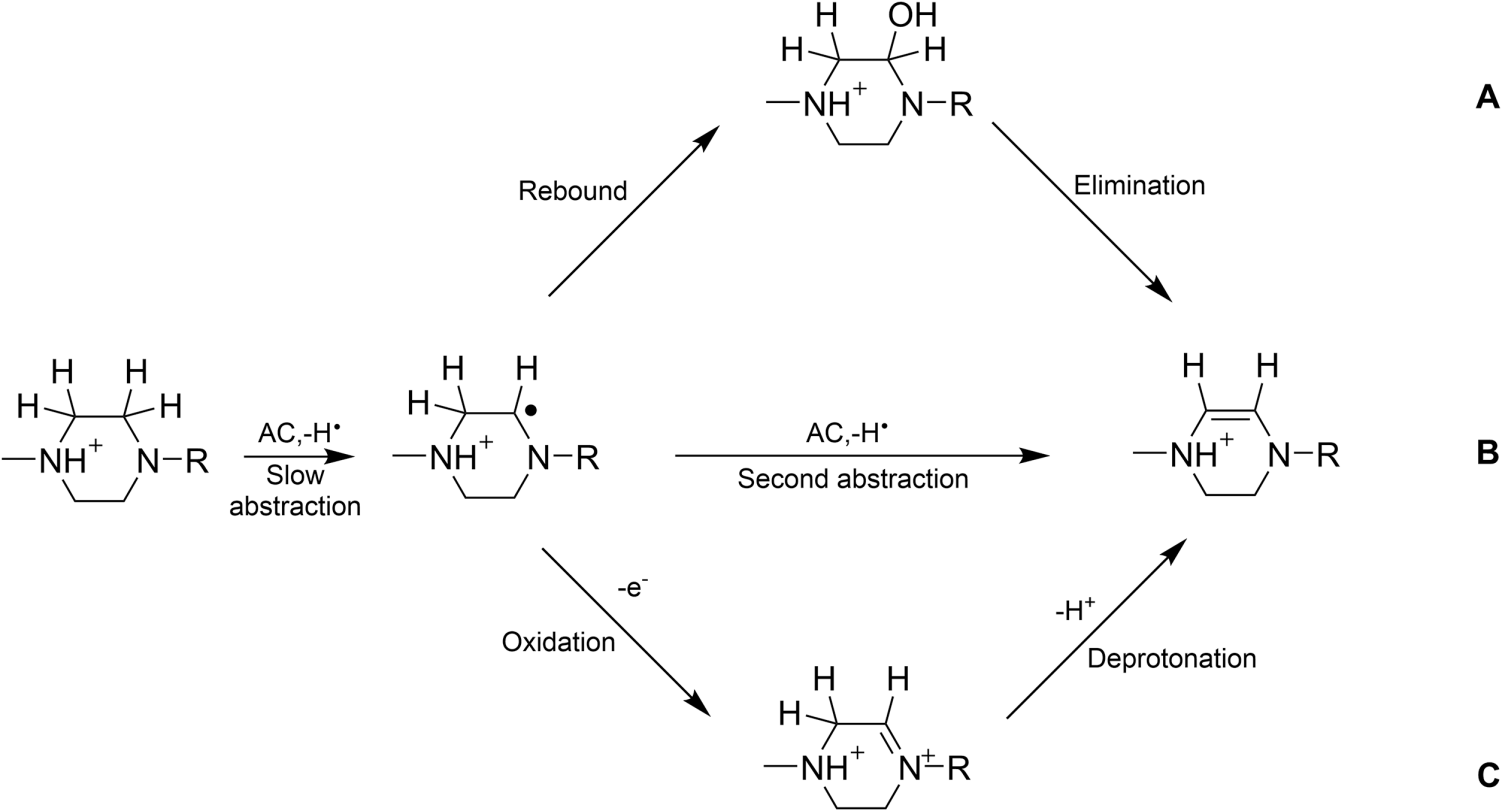
Potential Pathways for the TAML/H_2_O_2_ Desaturase Activity Following Rate-Limiting sp^3^ C–H Abstraction by the Activated Species: (A) Rebound-Elimination, (B) 2nd Radical Abstraction and, (C) Oxidation/Deprotonation

The desaturase activity of **2** is characterized by a large kinetic isotope effect, *k*_H_/*k*_D_ = 8.6, measured for the *k*_II_^A^ pathway indicative of an sp^3^ C–H bond cleavage in the rate-limiting step. Measurements of *k*_II_^A^ in the temperature range of 278–318 K (Table S4 and Figure S22) gave access to the activation parameters Δ*H* ^‡^ and Δ*S* ^‡^ of 37 ± 5 kJ mol^–1^ and −23 ± 2 J K^–1^ mol^–1^ for the desaturase activity of **2**.

Experiments with ofloxacin-*d*_8_ also confirmed that OA1 and OB0 are formed via parallel pathways as presented in Scheme 2. The concentration versus time profiles vary predictably when ofloxacin is selectively deuterated (Figure S23). Thus, more OB0 is produced when the piperazine ring methylene C–H bonds are rendered less reactive by deuteration (ofloxacin-*d*_8_). The qualitative competitive experiment and direct kinetic *k*_H_/*k*_D_ measurements gave consistent results, allowing consideration of the mechanistic options for the desaturase activity of TAML/peroxide system (Scheme 4), which have been inspired by studies of the relevant enzymatic processes.^45,51,55^ The p*K*_a_ values of ofloxacin are 3.3 for the carboxylic acid and 8.4 for the tertiary alkyl nitrogen of the piperazine ring in ofloxacin, indicative of the zwitterionic character of ofloxacin at pH 7.^56^ More precisely, at pH 7, 96.2% of the methylated amine is protonated. The presence of small amounts of unprotonated methyl-amine opens the possibility of some desaturation activity starting on the α-*C* atoms commensurate with the fact that DFT calculations suggest that abstraction does not occur on *C*-atoms that are α to protonated *N*-atoms (see text in Section 3.8) The amine at the piperazine/quinolinone is considerably less basic and therefore less protonated and the stabilization that its available lone pair can afford to support abstraction from one of its α-methylene CH’s probably translates to making these sites the primary locus of the opening H atom abstraction for desaturation (Scheme 4). However, in the tiny fraction of unprotonated more basic methylated-*N* at pH 7, the corresponding lone pair should be more radical-stabilizing (Scheme S1) and the acid–base equilibrium could continuously adjust, lead to a portion of desaturation proceeding via this route. Both routes are likely to be operating simultaneously. Below, we will mainly discuss the route shown in Scheme 4, because the methylated nitrogen is more basic, and therefore its unprotonated form should account for only a small fraction of the transformation. Again, the two routes share the same mechanism, differing only in the protonation site and the site of the initial abstraction.

The transformations that follow the rate limiting step are not kinetically observable, i.e., kinetics is typically silent regarding details of fast steps. In Scheme 4, we consider three intimate follow-on pathways (or processes) for the piperazine radical formed in the RDS to move on to the observed desaturase product — (A) rebound/elimination, (B) second H atom abstraction and, (C) electron transfer with deprotonation — as options based on prior literature proposals in this general area, and we integrate these with our own data and arguments to arrive at what we think is the most likely mechanism.

The rebound/elimination process A (Scheme 4), where rebound is yet to be established for C–H abstractions by TAML reactive intermediates, seems unlikely because while spontaneous dehydration of alkenes is known to occur under very acidic conditions and higher temperatures it is usually slow.^57^ The follow-up H-atom abstraction of process B to desaturation is of a type that has been widely considered in the literature.^45,58^ Process B might seem plausible here because the primary radical is a high energy species which may activate an adjacent C–H bond toward abstraction by a second AC. However, abstraction by the local Fe­(IV)­(OH) produced in the first abstraction, while appearing to be plausible for this activated sp^3^-C–H bond, is best considered highly unlikely based on the evidence of the DFT calculations we present below that characterize an exceptionally low reactivity for [TAMLFe­(IV)­(OH)]^−^ toward CH abstractions, but with confidence reduced by the likely high reactivity of the donor with its adjacent carbon radical. The electron transfer oxidation/deprotonation process C is inspired by the work of Kochi^59–61^ who showed that that alkyl radicals are oxidized by Cu^II^ in water with second-order rate constants as high as 10^8^ M^–1^ s^–1^. We consider this to be the most likely follow on pathway since the Cu^II/I^ reduction potential is significantly lower than that of TAML-Fe^IV/III^ (0.159 vs 0.87 V, respectively, in an aqueous environment).^62,63^ And the presence of the lone pair on the unprotonated nitrogen would favor the formation of an iminium cation intermediate (captured in DFT calculations-see Section 3.8) from which proton removal from the β-carbon would give the observed product.

To better understand the relative probabilities of the A–C processes and to help settle a long-standing uncertainty about the relative C–H bond abstracting reactivities of TAML-Fe species oxidized by one and two electrons above the resting ferric state, we have turned to DFT calculations (Section 3.8) that will be presented following our experimental analysis of the parallel demethylation process.

#### 3.6.2 Mechanism of the Ofloxacin Demethylation by TAML/H_2_O_2_

The fascination of metal complex and enzyme catalyzed oxidative cleavages of H_3_C–N bonds derives mechanistically from the rate-limiting sp^3^ C–H bond cleavages which play the dominant role in numerous catalytic transformations^64–66^ and practically from the wide ranging impacts of such reactions in pharmacology, toxicology, and biochemical pathways.^67^

The mechanism we suggest in Scheme 5 for the TAML/H_2_O_2_ N-demethylation processes has been quantified with **1a**, **1b**, **2** /H_2_O_2_ for ofloxacin by the rate constants *k*_II_^B^. Experiments with ofloxacin-*d*_3_ revealed a kinetic isotope effect *k*_H_/*k*_D_ of 3.8 (Table 2) supporting that the methyl C–H bond is the primary target of the active TAML species. Enthalpy and entropy of activation values, Δ*H* ^‡^ = 43 ± 4 kJ mol^–1^ and Δ*S* ^‡^ = −20 ± 1 J K^–1^ mol^–1±^, were also measured for the **2**/H_2_O_2_ *N*-demethylation *k*_II_^B^ pathway (Table S4 and Figure S22). These are similar to the corresponding values measured for the *k*_II_^A^ pathway, consistent with C–H bond cleavage in the rate-limiting step. The slightly higher value of Δ*H* ^‡^ for N-demethylation likely originates in the higher bond dissociation energy of primary versus secondary C–H bonds. Not surprisingly, entropic features are identical for the competing pathways. All mechanistically relevant results plus the options previously discussed by other workers^64,65^ lead us again to propose that process C in Scheme 4 is most likely; the methyl-*N*-deprotonation shown in Scheme 5 likely occurs prior to the abstraction with Pathway A carrying the bulk of the reacion as reflected in Scheme S1 for the desaturation process. Increasing pH leads to the formation of more OB0 demonstrating that deprotonation of the methylated nitrogen is a key step in the demethylation process (Figure S24). The initial radical abstraction of TAML/H_2_O_2_ conforms with the mechanistic conception of how *N*-dealkylation proceeds in cytochrome P450 and peroxidase enzymes,^64,65^ lending further credence to the idea that the TAMLs imitate enzymatic behavior faithfully, even if they do so at much greater rates. It is worth noting that *N*-demethylation is ca. 6 times slower than desaturation and *k*_H_/*k*_D_ is also lower. As with ΔΔ*H* ^‡^, these observations presumably reflect a higher dissociation energy of the NCH_2_–H versus NCH–H bond,^68^ but also could be influenced by the low concentration of the unprotonated methylated piperazine *N*-atom.

**Scheme 5:**
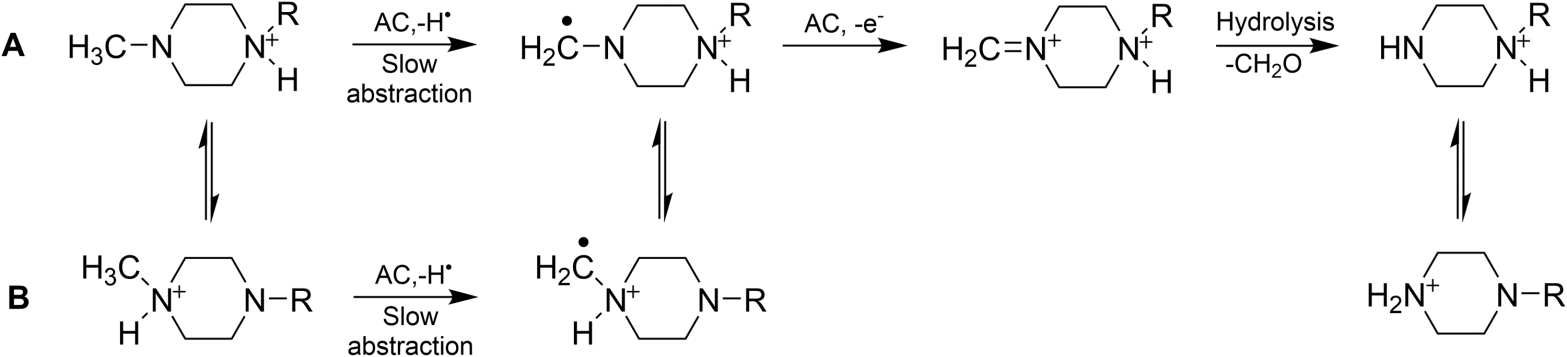
Suggested Pathways for the **2**/H_2_O_2_ Demethylation of the Tertiary Alkylamine during Ofloxacin Degradation by H_2_O_2_ with Rate-Limiting Hydrogen Atom Abstraction from the Methyl Group, with Proton Migration Occurring either before (Pathway A) or after (Pathway B) the Abstraction, followed by Electron-Transfer Oxidation and Hydrolysis Releasing Formaldehyde

### 3.7 Desaturation of 1-Methyl-4-phenylpiperazine and a Possible Ofloxacin Anchoring Effect in Its TAML Catalyzed Oxidation

The **2**/H_2_O_2_ desaturase reaction channel was found in both ofloxacin and its 1-methyl-4-phenylpiperazine surrogate oxidations. When 1-methyl-4-phenylpiperazine (10^–4^ M) was treated by **2**/H_2_O_2_ (2 × 10^–7^/5 × 10^–3^ M) for 20 min and then injected to the HR-ESI-MS instrument in the positive mode, both its desaturation and *N*-demethylation products were detected and several additional degradation products commensurate with Scheme 2 were also found (Scheme 6 and Figure S25).

**Scheme 6:**
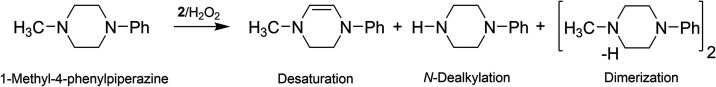
Identified Products of Degradation of 1-Methyl-4-phenylpiperazine by **2**/H_2_O_2_

In addition to these compounds, a distinct peak with *m*/*z* of 176.1309, which is 1 Da less than 1-methyl-4-phenylpiperazine, was also detected. The isotopic peaks of this compound are only 0.5 Da from the parent peak indicating that it is a [M+2H]^2+^ signal and its corresponding [M + H]^+^ peak at 351.2535 was also detected (Figure S26). These peaks match the signal of the 1-methyl-4-phenylpiperazine dimer, which might be formed by the coupling of two radicals produced after the first step of hydrogen abstraction. Interestingly, ofloxacin does not form such a dimer, which might be due to steric hindrance from its bulky substituents. This further suggests that the coupling site of the dimer is likely located on the piperazine ring. These observations provide additional support for the previously proposed desaturation mechanism of ofloxacin.

The kinetics of the **2**/H_2_O_2_ degradation of 1-methyl-4-phenylpiperazine was explored as described above for ofloxacin. Rate law 8 was confirmed (Figure S27) and the corresponding rate constants *k*_I_ and *k*_II_ are included in Table 1. The rate constant *k*_II_ is half that of ofloxacin adding a further observation to the theme that bigger molecules react faster than smaller ones in the presence of TAMLs,^69^ although this effect and its magnitude are likely to be substrate dependent. Since the aryl piperazine substituent in ofloxacin is more electron-withdrawing than the phenyl group of 1-methyl-4-phenylpiperazine, we doubt that the rate ordering reflects polarizations leading to bond strength differences as such effects should favor a slower reaction for ofloxacin. It seems more likely that this is a manifestation of an anchoring effect because bigger molecules with several potential binding centers with TAMLs may preorganize the oxidation events.

### 3.8 DFT Investigation

DFT calculations have proven to be useful in this study for (i) identifying the likely structure and oxidation state at iron of AC, and, (ii) examining the interactions of AC with the ofloxacin model compound, 1,4-dimethylpiperazine. Calculations showed that two **2** ACs, [(L^•+^)­Fe­(IV)­(O)]^−^ and (L^•+^)­Fe­(IV)­(OH) (selected molecular orbital contours are available in the Figures S28 and S29), both oxidized by two electrons above the ferric state, are reactive toward C–H bond abstraction. Moreover, while these are highly reactive toward monoprotonated 1,4-dimethylpiperazine, they are inert toward its doubly protonated form. This indicates that the presence of a deprotonated *N*-atom α to the carbon atom stabilizes via its lone pair the *C*-radical formed upon hydrogen atom abstraction (HAT) (which also removes the abstraction slowing positive charge on the *N*-atom) as a requirement for the abstraction processes. The one-electron oxidized species above the ferric state, [TAML-Fe­(IV)­(O)]^2–^ and [TAML-Fe­(IV)­(OH)]^−^, are both remarkably unreactive toward both mono- and diprotonated 1,4-dimethylpiperazine. The computed reaction barriers and the estimated rate constants for the HAT from 1,4-dimethylpiperazine are provided in the Table S5. The transition state structure of the HAT process for 1,4-dimethylpiperazine was also captured computationally and reported in the Figure S30.

Alongside the HAT, DFT calculations revealed a single electron transfer (SET) event that removed one electron from the carbon radical intermediate and led to the production of an iminium intermediate (molecular orbital contours graph in Figure S31). The iminium intermediate, also supported by conceptual thinking concerning organic reaction chemistry, highlights the important role of nitrogen in facilitating the HAT step and brings insight into the overall inert behavior of the two-electron oxidized ACs [**2**-O]^−^ and [**2**–OH] toward doubly protonated piperazine. The unprotonated nitrogen also provides support for why, in the **2**-catalyzed desaturation of ofloxacin, the process likelydoes not proceed with a second hydrogen abstraction or rebound/elimination following the initial hydrogen abstraction. Instead, the overall mechanistic narrative of ofloxacin dehydrogenation is a favored sequence of HAT, SET and final hydrolysis as summarized in Scheme 4C.

## 4 Discussion

Inspired initially by oxidizing enzymes, TAML catalysts have evolved through iterative design to far surpass their progenitors in reactivity, exhibiting faster reactions while deeply oxidizing and typically near-mineralizing persistent micropollutants that natural enzymes struggle to break down. The fluoroquinolone subjects of this work are cases in point. TAML **2** is one of the most advanced TAMLs to date. It activates H_2_O_2_ to deliver a biotranscendent oxidative destruction of fluoroquinolones that are water pollutants of concern (and of global significance as such) that minimizes toxicity concerns by the extreme depth of oxidation it easily achieves. The implications of this study overall are not confined just to signaling virtually ideal degradation processes for trace fluoroquinolones in water, but rather offer an advanced picture for understanding how TAML catalysis work to safely and sustainably neutralize micropollutants in general.

### 4.1 Early and Larger Degradation Products

The primary early products of **2**/H_2_O_2_ degradation of ofloxacin have been reliably identified as dehydroofloxacin (OA1) and desmethylofloxacin (OB0) to provide a solid foundation for identification of subsequent products (Scheme 2). The temporal concentration dependence has been measured for all detected intermediates. Example profiles for the **2**/H_2_O_2_ destruction of ofloxacin are presented in Figures 2 and 3.

### 4.2 Evidence for Safety and Sustainability of the 2/H_2_O_2_/Ofloxacin System

To develop a weight of evidence that the **2**/H_2_O_2_ intensified oxidative mineralization of any micropollutant or mixture of micropollutants is likely to be safe and sustainable by design (SSbD) in the context of TAML catalysts used in low nM quantities that also degrade in use, one must first examine the nature and toxicity of all degradation intermediates in recognition of the possibility that one or more could be toxic, even more toxic, than the initial target(s). The ability of **2**/H_2_O_2_ to effectively mineralize fluoroquinolones, and many other micropollutants by prior demonstration and implication,^27,69–73^ offers credence to the proposition that intermediate toxicities can be safely managed by setting operational parameters to ensure the near-complete destruction of toxic nonmineral intermediates prior to release of treated waters to the environment.

In addressing this challenge, the value of degradation toxicity profiles has been recently highlighted by us^37^ and other workers.^74,75^ In the absence of experimental data on the toxicities of the degradation products presented in Scheme 2, we deployed the Toxicity Estimation Software Tool (T.E.S.T., US EPA).^76^ According to the consensus method in T.E.S.T, the predicted fathead minnow LC_50_ (96 h) of ofloxacin and its degradation products are shown in Figure 8a, where the two parallel pathways show similar toxicity trends and the final detectable bigger intermediate OA7 shows a nearly 4-fold decrease in toxicity compared to ofloxacin. The data in Figures 3 and 8a were used to create the comprehensive toxicity profile in Figure 8b which shows the toxicity variation in the **2**/H_2_O_2_/ofloxacin system. Despite a slight rise in normalized toxicity in the early stages, the subsequent degradation of the primary products OA1 and OB0 led to a rapid decrease in toxicity to ultimately eliminate more than 99% of the relative toxicity of ofloxacin. The final intermediate in Scheme 2 (OA7) continues to degrade toward minerals, a process that is boosted by the addition of a second aliquot of **2** (Figure 8b). This analysis contributes to the weight of evidence that the **2**/H_2_O_2_/ofloxacin system is safe and sustainable and fits extremely well into the field the European Commission is encouraging the chemical enterprise to build to populate Europe’s chemical technologies of the future called the *Safe and Sustainable by Design* (SSbD) framework as well as working toward the United Nation Sustainable Development Goals.^10,77^ We believe the properties of TAML/peroxide for removing micropollutants from water are iconic of the goals the EU has set for SSbD processes.

**Figure 8:**
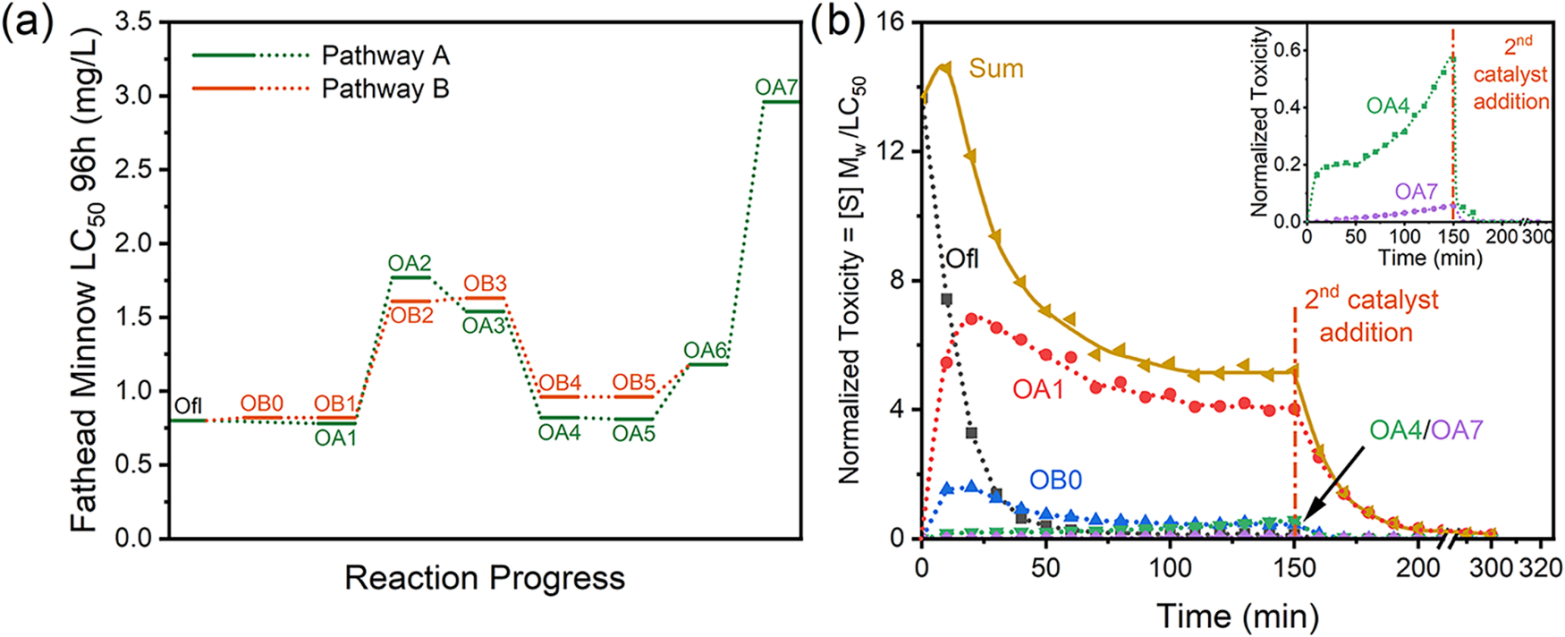
(a) Predicted fathead minnow LC_50_ (96h) of ofloxacin and its identified degradation products. (b) The comprehensive toxicity profile for the **2**/H_2_O_2_/ofloxacin system.

### 4.3 Identification of Small Molecule Degradation Products

Small molecules generated are usually much less toxic than the pollutants themselves and the larger intermediates, but it is important to identify them as well to be sure. The ions F^–^, CH_3_COO^–^, HCOO^–^, NO_2_^–^ and NO_3_^–^ were identified in this work by ion chromatography, providing direct and strong evidence that **2**/H_2_O_2_ is capable of mineralizing bioresistant micropollutants like the fluoroquinolones.

### 4.4 Mechanistic Challenges

Thirteen bigger compounds are shown in Scheme 2 and the mechanisms of transformation of each may be examined. Naturally, it is hardly possible to characterize the mechanistic details for all 13 and hence, the most accessible intermediates were selected for a deeper mechanistic look, particularly the TAML/H_2_O_2_ generated dehydro- and desmethyl-derivatives. Though these enzyme-mimicking processes result in the cleavage of the C–H and C–H plus C–N bonds, respectively, they have been found to be mechanistically similar because both involve the rate-limiting activation of sp^3^ C–H bonds as verified by kinetic isotope effect studies. The kinetic data reveal that the AC of **2**, accomplishes hydrogen abstraction with facility. The short-lived carbon-centered radical products then transform rapidly to give the final products as it is shown in Schemes 4 and 5.

DFT calculations added support to the hydrogen abstraction mechanism for the desaturation process and revealed that a two-electron oxidized species and not a one-electron oxidized species above the ferric state is likely to be the active species. The study also shows that TAML/H_2_O_2_ exhibits aggressive desaturase activity as a novel expression of the well-established peroxidase activity, thereby permitting us to further enrich understanding of TAML catalysis and to cement the conception of its biotranscendent capabilities.

### 4.5 Practicality

The compositions of real waters, such as municipal wastewater, are usually complex and the concentrations of micropollutants are typically very low (ppb–ppt). To emphasize the treatment dexterity of **2**/H_2_O_2_, three studied fluoroquinolone antibiotics were spiked into primary (after sedimentation of solid waste) and secondary (after biological treatment) municipal wastewater. In both cases, the fluoroquinolone trio could be effectively removed using 20 nM **2** and 3.4 ppm (secondary wastewater)/6.8 ppm (primary wastewater) H_2_O_2_ (Figure S32). The initial concentrations of fluoroquinolones are similar to those of significantly contaminated municipal wastewaters pointing to the practical implications of the work.^78^ These properties entail astoundingly positive implications for municipal wastewater treatment.

## 5 Conclusion

The biotranscendent catalytic oxidation of fluoroquinolone antibiotics including ofloxacin and its analogs, ciprofloxacin and norfloxacin, by **2**/H_2_O_2_ in neutral aqueous media starts with the rate limiting abstraction of primary and secondary sp^3^ C–H bonds on the piperazine unit (*N*-methylpiperazine in the case of ofloxacin) and the ofloxacin model compound, 1-methyl-4-phenylpiperazine, resulting via parallel pathways in the formation of a C=C bond and ring N-demethylation, desaturation and demethylation, respectively. The former process indicates that TAML activators possess desaturase activity typically displayed by iron-containing α-ketoglutarate-dependent dioxygenase enzymes. Evidence for the nature of the activated species in these processes was obtained by DFT computational methods which highlight that the two-electron oxidation species above the ferric resting state is necessary for abstraction of a hydrogen atom from a sp^3^ carbon and that one-electron oxidation at iron does not suffice. Additionally, the abstraction from a pyrazine heterocycle requires that at least one nitrogen atom should be deprotonated. Thirteen bigger intermediates with exact masses of [M + H^+^] ranging from 392.13 to 279.08 Da form that were clearly identified or postulated based on limited evidence. The smallest and final of these, 10-amino-9-fluoro-3-methyl-7-oxo-2,3-dihydro-7*H*-[1,4]­oxazino­[2,3,4-*ij*]­quinoline-6-carboxylate (OA7) degrades further to small ions F^–^, CH_3_COO^–^, HCOO^–^, NO_2_^–^, and NO_3_^–^. A toxicity profile for the parallel sequences constructed using the T.E.S.T system for predicting fathead minnow LC_50_ (96 h) values supports the conclusion that **2**/H_2_O_2_ effectively eliminates the toxicity of ofloxacin. The demonstrated removal of fluoroquinolones by **2**/H_2_O_2_ under ultradilute catalysis conditions from both primary and secondary municipal wastewaters demonstrates that biotranscendent catalytic oxidation by **2**/H_2_O_2_ (**2** decomposes in the catalytic functioning media) promises to be of great practical value toward building a safer and more sustainable chemical enterprise while serving as an example of the type of chemical technologies the European Union is seeking through its safe-and-sustainable-by-design (SSbD) framework.

## Supplementary Material





## Notes

ESI-MS analysis of the degradation products, ^13^C NMR spectra of ofloxacin and OA1, DFT calculation of the noncovalent associate between **2** and ofloxacin, calibration curve in ion chromatogram analysis, ^19^F NMR data, detailed kinetic curves, activation parameters calculation, DFT calculations and an experimental example of the wastewater application. Tables as described in the text, including HPLC and LC-MS methods, and rate constants for the **2**-catalyzed degradation of ofloxacin by H_2_O_2_ at pH 7 under different temperatures (PDF)
Molecular coordinates for the DFT calculations (PDF)

## References

[1] Werner N. L., Hecker M. T., Sethi A. K., Donskey C. J. (2011). Unnecessary Use
of Fluoroquinolone Antibiotics in Hospitalized Patients. BMC Infect. Dis..

[2] Thai V.-A., Dang V. D., Thuy N. T., Pandit B., Vo T.-K.-Q., Khedulkar A. P. (2023). Fluoroquinolones:
Fate, Effects on the Environment
and Selected Removal Methods. J. Cleaner Prod..

[3] Kümmerer K. (2009). Antibiotics
in the Aquatic Environment – A Review – Part I. Chemosphere.

[4] Gangar T., Patra S. (2023). Antibiotic Persistence
and Its Impact on the Environment. 3 Biotech.

[5] Khetan S. K., Collins T. J. (2007). Human Pharmaceuticals in the Aquatic Environment: A
Challenge to Green Chemistry. Chem. Rev..

[6] Van
Doorslaer X., Dewulf J., Van Langenhove H., Demeestere K. (2014). Fluoroquinolone Antibiotics: An Emerging Class of Environmental
Micropollutants. Sci. Total Environ..

[7] Hughes S. R., Kay P., Brown L. E. (2013). Global Synthesis and Critical Evaluation of Pharmaceutical
Data Sets Collected from River Systems. Environ.
Sci. Technol..

[8] Gomez Cortes, L. ; Marinov, D. ; Sanseverino, I. ; Navarro Cuenca, A. ; Niegowska, M. ; Porcel Rodriguez, E. ; Stefanelli, F. ; Lettieri, T. ; Joint Research Centre (European Commission) . Selection of Substances for the 4th Watch List under the Water Framework Directive; Publications Office of the European Union, 2022.

[9] Yang Y., Zhang X., Jiang J., Han J., Li W., Li X., Yee Leung K. M., Snyder S. A., Alvarez P. J. J. (2022). Which Micropollutants
in Water Environments Deserve More Attention Globally?. Environ. Sci. Technol..

[10] European Commission . Safe and Sustainable by Design. https://research-and-innovation.ec.europa.eu/research-area/industrial-research-and-innovation/chemicals-and-advanced-materials/safe-and-sustainable-design_en. (accessed July 18, 2025).

[11] Collins T. J., Ryabov A. D. (2017). Targeting of High-Valent Iron-TAML Activators at Hydrocarbons
and Beyond. Chem. Rev..

[12] Warner G. R., Somasundar Y., Jansen K. C., Kaaret E. Z., Weng C., Burton A. E., Mills M. R., Shen L. Q., Ryabov A. D., Pros G., Pintauer T., Biswas S., Hendrich M. P., Taylor J. A., Vom Saal F. S., Collins T. J. (2019). Bioinspired, Multidisciplinary,
Iterative Catalyst Design Creates the Highest Performance Peroxidase
Mimics and the Field of Sustainable Ultradilute Oxidation Catalysis
(SUDOC). ACS Catal..

[13] Ma X., Johnson C. R., Schafer M. C., van der Linde M. M., Ryabov A. D., Collins T. J. (2024). Advancing the Sustainability
of the
Pharmaceutical Industry: TAML/Peroxide Destroys Trace Pharmaceuticals
Where Unprecedented Efficiencies Increase with Decreasing TAML Concentrations. ACS Sustainable Chem. Eng..

[14] Panda C., Dhar B. B., Malvi B., Bhattacharjee Y., Sen Gupta S. (2013). Catalytic Signal Amplification Using
[Fe III (Biuret-Amide)]-Mesoporous
Silica Nanoparticles : Visual Cyanide Detection. Chem. Commun..

[15] Frisch, M. J. ; Trucks, G. W. ; Schlegel, H. B. ; Scuseria, G. E. ; Robb, M. A. ; Cheeseman, J. R. ; Scalmani, G. ; Barone, V. ; Petersson, G. A. ; Nakatsuji, H. Gaussian 16 Rev. C.01; Gaussian, Inc.: Wallingford CT, 2016.

[16] Zhao Y., Truhlar D. G. (2006). A New Local Density
Functional for Main-Group Thermochemistry,
Transition Metal Bonding, Thermochemical Kinetics, and Noncovalent
Interactions. J. Chem. Phys..

[17] Chai J.-D., Head-Gordon M. (2008). Systematic
Optimization of Long-Range Corrected Hybrid
Density Functionals. J. Chem. Phys..

[18] Marenich A. V., Cramer C. J., Truhlar D. G. (2009). Universal
Solvation Model Based on
Solute Electron Density and on a Continuum Model of the Solvent Defined
by the Bulk Dielectric Constant and Atomic Surface Tensions. J. Phys. Chem. B.

[19] de
Oliveira F. T., Chanda A., Banerjee D., Shan X., Mondal S., Que L., Bominaar E. L., Münck E., Collins T. J. (2007). Chemical and Spectroscopic Evidence for an FeV-Oxo
Complex. Science.

[20] Chanda A., Shan X., Chakrabarti M., Ellis W. C., Popescu D. L., Tiago de Oliveira F., Wang D., Que L., Collins T. J., Münck E., Bominaar E. L. (2008). (TAML)­FeIV=O
Complex in Aqueous Solution: Synthesis and Spectroscopic and Computational
Characterization. Inorg. Chem..

[21] Somasundar Y., Park M., Daniels K. D., Warner G. R., Ryabov A. D., Snyder S. A., Collins T. J. (2021). Transformative Catalysis Purifies
Municipal Wastewater of Micropollutants. ACS
ES&T Water.

[22] Al-Omar, M. A. Ofloxacin. In Profiles of Drug Substances, Excipients and Related Methodology; Elsevier, 2009; Vol. 34, pp 265–298 10.1016/S1871-5125(09)34006-6. 22469176

[23] Qi J., Gao X.-X., Zhao M.-X., Xiang J.-F., Lin C.-X., Xu Y.-Z., Wu J.-G. (2007). Studies on NMR Behavior of Ofloxacin
in Different pH Environment. Chem. J. Chin.
Univ..

[24] Holzgrabe U., Branch S. K. (1994). 1H, 19F and 13C NMR Spectral Data of Fifteen Gyrase
Inhibitors and Some Metabolites. Magn. Reson.
Chem..

[25] Sagdinc S., Bayarı S. (2004). Spectroscopic Studies on the Interaction of Ofloxacin
with Metals. J. Mol. Struct..

[26] Somasundar Y., Shen L. Q., Hoane A. G., Tang L. L., Mills M. R., Burton A. E., Ryabov A. D., Collins T. J. (2018). Structural, Mechanistic,
and Ultradilute Catalysis Portrayal of Substrate Inhibition in the
TAML–Hydrogen Peroxide Catalytic Oxidation of the Persistent
Drug and Micropollutant, Propranolol. J. Am.
Chem. Soc..

[27] Shen L. Q., Beach E. S., Xiang Y., Tshudy D. J., Khanina N., Horwitz C. P., Bier M. E., Collins T. J. (2011). Rapid, Biomimetic
Degradation in Water of the Persistent Drug Sertraline by TAML Catalysts
and Hydrogen Peroxide. Environ. Sci. Technol..

[28] Do
Pham D. D., Kelso G. F., Yang Y., Hearn M. T. W. (2014). Studies
on the Oxidative N-Demethylation of Atropine, Thebaine and Oxycodone
Using a Fe III -TAML Catalyst. Green Chem..

[29] Chen R., Ding S., Fu N., Ren X. (2023). Preparation of a G-C3N4/Ag3PO4
Composite Z-Type Photocatalyst and Photocatalytic Degradation of Ofloxacin:
Degradation Performance, Reaction Mechanism, Degradation Pathway and
Toxicity Evaluation. J. Environ. Chem. Eng..

[30] Chen X., Yao J., Xia B., Gan J., Gao N., Zhang Z. (2020). Influence
of pH and DO on the Ofloxacin Degradation in Water by UVA-LED/TiO2
Nanotube Arrays Photocatalytic Fuel Cell: Mechanism, ROSs Contribution
and Power Generation. J. Hazard. Mater..

[31] Wang Z., Cai X., Xie X., Li S., Zhang X., Wang Z. (2021). Visible-LED-Light-Driven
Photocatalytic Degradation of Ofloxacin and Ciprofloxacin by Magnetic
Biochar Modified Flower-like Bi2WO6: The Synergistic Effects, Mechanism
Insights and Degradation Pathways. Sci. Total
Environ..

[32] Rajagopalan A., Lara M., Kroutil W. (2013). Oxidative Alkene Cleavage by Chemical
and Enzymatic Methods. Adv. Synth. Catal..

[33] Takemoto M., Iwakiri Y., Suzuki Y., Tanaka K. (2004). A Mild Procedure for
the Oxidative Cleavage of Substituted Indoles Catalyzed by Plant Cell
Cultures. Tetrahedron Lett..

[34] Dhakshinamoorthy A., Pitchumani K. (2006). Clay-Anchored
Non-Heme Iron–Salen Complex Catalyzed
Cleavage of CC Bond in Aqueous Medium. Tetrahedron.

[35] Gonzalez-de-Castro A., Xiao J. (2015). Green and Efficient: Iron-Catalyzed Selective Oxidation of Olefins
to Carbonyls with O2. J. Am. Chem. Soc..

[36] Chen P., Blaney L., Cagnetta G., Huang J., Wang B., Wang Y., Deng S., Yu G. (2019). Degradation of Ofloxacin
by Perylene Diimide Supramolecular Nanofiber Sunlight-Driven Photocatalysis. Environ. Sci. Technol..

[37] Somasundar Y., Burton A. E., Mills M. R., Zhang D. Z., Ryabov A. D., Collins T. J. (2021). Quantifying Evolving
Toxicity in the TAML/Peroxide
Mineralization of Propranolol. iScience.

[38] Horwitz C. P., Fooksman D. R., Vuocolo L. D., Gordon-Wylie S. W., Cox N. J., Collins T. J. (1998). Ligand Design Approach for Securing
Robust Oxidation Catalysts. J. Am. Chem. Soc..

[39] Emelianenko M., Torrejon D., DeNardo M. A., Socolofsky A. K., Ryabov A. D., Collins T. J. (2014). Estimation
of Rate Constants in Nonlinear
Reactions Involving Chemical Inactivation of Oxidation Catalysts. J. Math. Chem..

[40] Mathon B., Coquery M., Liu Z., Penru Y., Guillon A., Esperanza M., Miège C., Choubert J.-M. (2021). Ozonation of 47
Organic Micropollutants in Secondary Treated Municipal Effluents:
Direct and Indirect Kinetic Reaction Rates and Modelling. Chemosphere.

[41] Dodd M. C., Buffle M.-O., von Gunten U. (2006). Oxidation
of Antibacterial Molecules
by Aqueous Ozone: Moiety-Specific Reaction Kinetics and Application
to Ozone-Based Wastewater Treatment. Environ.
Sci. Technol..

[42] Ling W., Ben W., Xu K., Zhang Y., Yang M., Qiang Z. (2018). Ozonation
of Norfloxacin and Levofloxacin in Water: Specific Reaction Rate Constants
and Defluorination Reaction. Chemosphere.

[43] Liu Y., You T., Wang H.-X., Tang Z., Zhou C.-Y., Che C.-M. (2020). Iron- and
Cobalt-Catalyzed C­(Sp 3)–H Bond Functionalization Reactions
and Their Application in Organic Synthesis. Chem. Soc. Rev..

[44] Liu B., Romine A. M., Rubel C. Z., Engle K. M., Shi B.-F. (2021). Transition-Metal-Catalyzed,
Coordination-Assisted Functionalization of Nonactivated C­(Sp3)–H
Bonds. Chem. Rev..

[45] Bryliakov K. P. (2024). Mechanisms
of C­(Sp3)–H and C=C Selective Oxidative Heterofunctionalizations
by Non-Heme Fe and Mn Mimics of Oxygenase Enzymes. Coord. Chem. Rev..

[46] Zhang Z., Chen P., Liu G. (2022). Copper-Catalyzed Radical
Relay in
C­(Sp 3)–H Functionalization. Chem. Soc.
Rev..

[47] Li B., Elsaid M., Ge H. (2022). Transition-Metal-Catalyzed Site-Selective
γ- and δ-C­(Sp3)–H Functionalization Reactions. Chem.

[48] Gol’dshleger N. F., Tyabin M. B., Shilov A. E., Shteinman A. A. (2018). Activation of Saturated Hydrocarbons. Deuterium-Hydrogen
Exchange
in Solution of Transition Metal Complexes. Russ.
J. Phys. Chem..

[49] Ghosh A., Mitchell D. A., Chanda A., Ryabov A. D., Popescu D. L., Upham E. C., Collins G. J., Collins T. J. (2008). Catalase–Peroxidase
Activity of Iron­(III)–TAML Activators of Hydrogen Peroxide. J. Am. Chem. Soc..

[50] Papadopoulou A., Meyer F., Buller R. M. (2023). Engineering
Fe­(II)/α-Ketoglutarate-Dependent
Halogenases and Desaturases. Biochemistry.

[51] Gao S.-S., Naowarojna N., Cheng R., Liu X., Liu P. (2018). Recent Examples
of α-Ketoglutarate-Dependent Mononuclear Non-Haem Iron Enzymes
in Natural Product Biosyntheses. Nat. Prod.
Rep..

[52] Dunham N. P., Chang W., Mitchell A. J., Martinie R. J., Zhang B., Bergman J. A., Rajakovich L. J., Wang B., Silakov A., Krebs C., Boal A. K., Bollinger J. M. (2018). Two Distinct Mechanisms for C–C Desaturation
by Iron­(II)- and 2-(Oxo)­Glutarate-Dependent Oxygenases: Importance
of α-Heteroatom Assistance. J. Am. Chem.
Soc..

[53] DeNardo M. A., Mills M. R., Ryabov A. D., Collins T. J. (2016). Unifying Evaluation
of the Technical Performances of Iron-Tetra-Amido Macrocyclic Ligand
Oxidation Catalysts. J. Am. Chem. Soc..

[54] Pal P., Schafer M. C., Hendrich M. P., Ryabov A. D., Collins T. J. (2023). The Mechanism
of Formation of Active Fe-TAMLs Using HClO Enlightens Design for Maximizing
Catalytic Activity at Environmentally Optimal, Circumneutral pH. Inorg. Chem..

[55] Cerone M., Smith T. K. (2022). Desaturases: Structural
and Mechanistic Insights into
the Biosynthesis of Unsaturated Fatty Acids. IUBMB Life.

[56] Ghosh B. C., Deb N., Mukherjee A. K. (2010). Determination of Individual Proton Affinities of Ofloxacin
from Its UV–Vis Absorption, Fluorescence and Charge-Transfer
Spectra: Effect of Inclusion in β-Cyclodextrin on the Proton
Affinities. J. Phys. Chem. B.

[57] Vinnik M. I., Obraztsov P. A. (1990). The Mechanism
of the Dehydration of Alcohols and the
Hydration of Alkenes in Acid Solution. Russ.
Chem. Rev..

[58] Hull J. F., Balcells D., Sauer E. L. O., Raynaud C., Brudvig G. W., Crabtree R. H., Eisenstein O. (2010). Manganese
Catalysts for C–H
Activation: An Experimental/Theoretical Study Identifies the Stereoelectronic
Factor That Controls the Switch between Hydroxylation and Desaturation
Pathways. J. Am. Chem. Soc..

[59] Kochi J. K. (1967). Mechanisms
of Organic Oxidation and Reduction by Metal Complexes. Science.

[60] Kochi J. K., Subramanian R. V. (1965). Kinetics
of Electron-Transfer Oxidation of Alkyl Radicals
by Copper­(II) Complexes. J. Am. Chem. Soc..

[61] Kochi J. K., Bemis A., Jenkins C. L. (1968). Mechanism of Electron Transfer Oxidation
of Alkyl Radicals by Copper­(II) Complexes. J.
Am. Chem. Soc..

[62] Popescu D.-L., Vrabel M., Brausam A., Madsen P., Lente G., Fabian I., Ryabov A. D., van Eldik R., Collins T. J. (2010). Thermodynamic, Electrochemical, High-Pressure
Kinetic,
and Mechanistic Studies of the Formation of Oxo FeIV–TAML Species
in Water. Inorg. Chem..

[63] Cho S. K., Kim H. C., Kim M. J., Kim J. J. (2016). Voltammetric Observation
of Transient Catalytic Behavior of SPS in Copper Electrodeposition—Its
Interaction with Cuprous Ion from Comproportionation. J. Electrochem. Soc..

[64] Karki S. B., Dinnocenzo J. P., Jones J. P., Korzekwa K. R. (1995). Mechanism of Oxidative
Amine Dealkylation of Substituted N,N-Dimethylanilines by Cytochrome
P-450: Application of Isotope Effect Profiles. J. Am. Chem. Soc..

[65] Miwa G. T., Walsh J. S., Kedderis G. L., Hollenberg P. F. (1983). The Use
of Intramolecular Isotope Effects to Distinguish between Deprotonation
and Hydrogen Atom Abstraction Mechanisms in Cytochrome P-450- and
Peroxidase-Catalyzed N-Demethylation Reactions. J. Biol. Chem..

[66] Ouyang K., Hao W., Zhang W.-X., Xi Z. (2015). Transition-Metal-Catalyzed Cleavage
of C–N Single Bonds. Chem. Rev..

[67] Guengerich F. P. (2008). Cytochrome
P450 and Chemical Toxicology. Chem. Res. Toxicol..

[68] Wayner D.
D. M., Clark K. B., Rauk A., Yu D., Armstrong D. A. (1997). C–H
Bond Dissociation Energies of Alkyl Amines: Radical Structures and
Stabilization Energies. J. Am. Chem. Soc..

[69] Frame H. C., Shen L. Q., Ryabov A. D., Collins T. J. (2024). Two Major Pathways
in TAML-Catalyzed Degradation of Carbendazim by H2O2: Elimination
of a Nitrogen-Rich Benzimidazole Fungicide and Its Parallel Nitration. ACS ES&T Water.

[70] Pinzón-Espinosa A., Collins T. J., Kanda R. (2021). Detoxification of Oil Refining Effluents
by Oxidation of Naphthenic Acids Using TAML Catalysts. Sci. Total Environ..

[71] Gupta S. S., Stadler M., Noser C. A., Ghosh A., Steinhoff B., Lenoir D., Horwitz C. P., Schramm K.-W., Collins T. J. (2002). Rapid Total
Destruction of Chlorophenols by Activated Hydrogen Peroxide. Science.

[72] Mills M. R., Arias-Salazar K., Baynes A., Shen L. Q., Churchley J., Beresford N., Gayathri C., Gil R. R., Kanda R., Jobling S., Collins T. J. (2015). Removal of Ecotoxicity of 17α-Ethinylestradiol
Using TAML/Peroxide Water Treatment. Sci. Rep..

[73] Tang L. L., DeNardo M. A., Gayathri C., Gil R. R., Kanda R., Collins T. J. (2016). TAML/H2O2 Oxidative
Degradation of Metaldehyde: Pursuing
Better Water Treatment for the Most Persistent Pollutants. Environ. Sci. Technol..

[74] Egorova K. S., Galushko A. S., Ananikov V. P. (2020). Introducing
Tox-Profiles of Chemical
Reactions. Angew. Chem., Int. Ed..

[75] Egorova K. S., Galushko A. S., Dzhemileva L. U., D’yakonov V. A., Ananikov V. P. (2021). Building Bio-Profiles for Common Catalytic Reactions. Green Chem..

[76] US EPA, O . Toxicity Estimation Software Tool (TEST). https://www.epa.gov/comptox-tools/toxicity-estimation-software-tool-test. (accessed July 24, 2025).

[77] UNDP . Sustainable Development Goals. https://www.undp.org/sustainable-development-goals. (accessed July 24, 2025).

[78] Liu X., Zhang G., Liu Y., Lu S., Qin P., Guo X., Bi B., Wang L., Xi B., Wu F., Wang W., Zhang T. (2019). Occurrence and Fate of Antibiotics
and Antibiotic Resistance Genes in Typical Urban Water of Beijing,
China. Environ. Pollut..

[79] Boerner, T. J. ; Deems, S. ; Furlani, T. R. ; Knuth, S. L. ; Towns, J. In ACCESS: Advancing Innovation: NSF’s Advanced Cyberinfrastructure Coordination Ecosystem: Services & Support, Practice and Experience in Advanced Research Computing 2023: Computing for the Common Good, PEARC ’23; Association for Computing Machinery: New York, NY, USA, 2023; pp 173–176 10.1145/3569951.3597559.

[80] Brown, S. T. ; Buitrago, P. ; Hanna, E. ; Sanielevici, S. ; Scibek, R. ; Nystrom, N. A. In Bridges-2: A Platform for Rapidly-Evolving and Data Intensive Research, Practice and Experience in Advanced Research Computing 2021: Evolution Across All Dimensions, PEARC ’21; Association for Computing Machinery: New York, NY, USA, 2021; pp 1–4.

