# Supplementary material for: Beyond Mimicking Enzymes: NewTAML/Peroxide Abstracts sp3 C–H Bonds to Initiate Biotranscendent Water-Purifying Mineralization of Fluoroquinolone Antibiotics
Source: J Am Chem Soc. 2025 Dec 23;148(1):292–307. doi: 10.1021/jacs.5c12768 (PMC12814164; doi:10.1021/jacs.5c12768)
Supplement: Supplementary file 1 [file ja5c12768_si_001.pdf]

Supporting Information For

Beyond mimicking enzymes: NewTAML/peroxide abstracts sp^3 C–H bonds to initiate biotranscendent water-purifying mineralization of fluoroquinolone antibiotics

Xiaowei Ma, Longzhu Q. Shen*, Minerva G. Schafer, Karl H. G. Schulz, Roberto R. Gil, Alexander D. Ryabov*, and Terrence J. Collins*

Institute for Green Science, Department of Chemistry, 4400 Fifth Avenue, Carnegie Mellon University, Pittsburgh, Pennsylvania 15213, United States

*To whom correspondence should be addressed: T.J.C. (tc1u@andrew.cmu.edu); A.D.R (ryabov@andrew.cmu.edu); L.Q.S (lshen@andrew.cmu.edu).

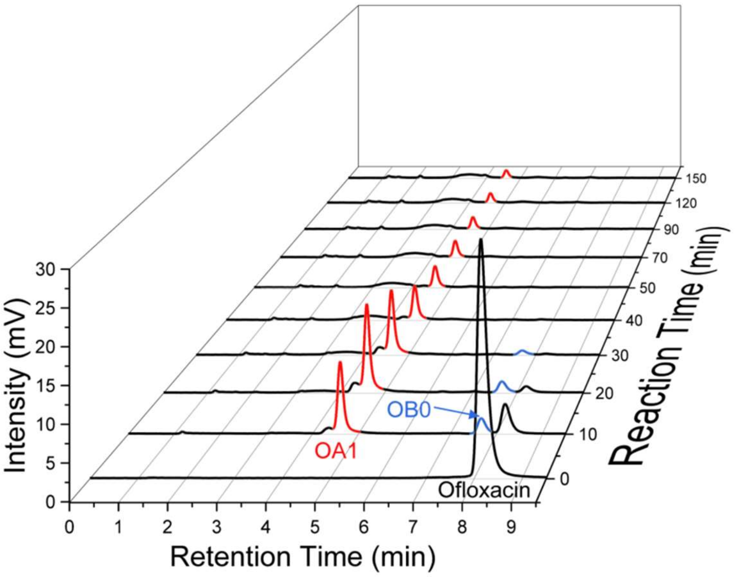

Figure S1. HPLC graphs of ofloxacin degradation by **2**/H_2O_2 at different reaction times. Conditions: [ofloxacin] = 3×10^{-5} M, [**2**] = 2×10^{-7} M, [H_2O_2] = 1×10^{-3} M, pH 7.0 (0.01 M phosphate), 25 °C.

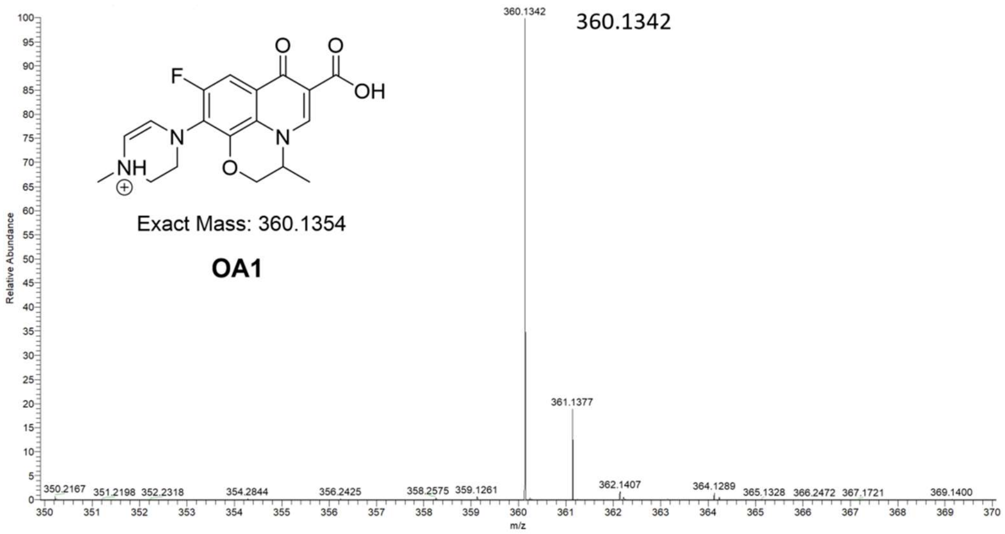

Figure S2. The HR-ESI-MS spectrum of product OA1.

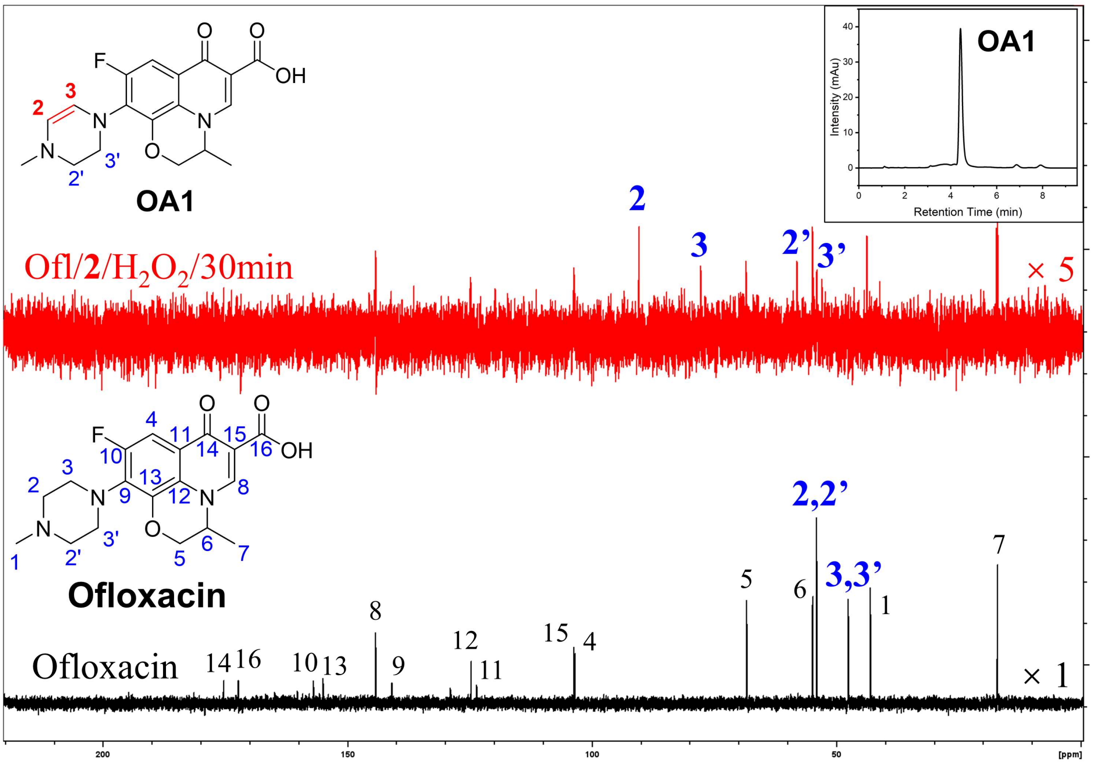

Figure S3. ^{13}C NMR spectra of ofloxacin (black) and reaction mixture after 30 min reaction (red). Conditions: [ofloxacin] = 5×10^{-3} M, [**2**] = 2.5×10^{-5} M, [H_2O_2] = 1×10^{-2} M, D_2O, 25 °C.

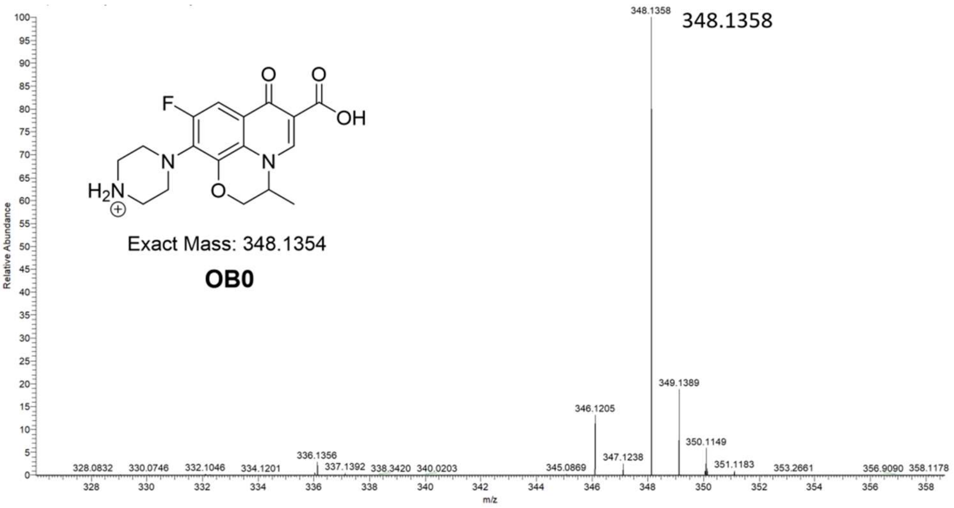

Figure S4. The HR-ESI-MS spectrum of product OB0.

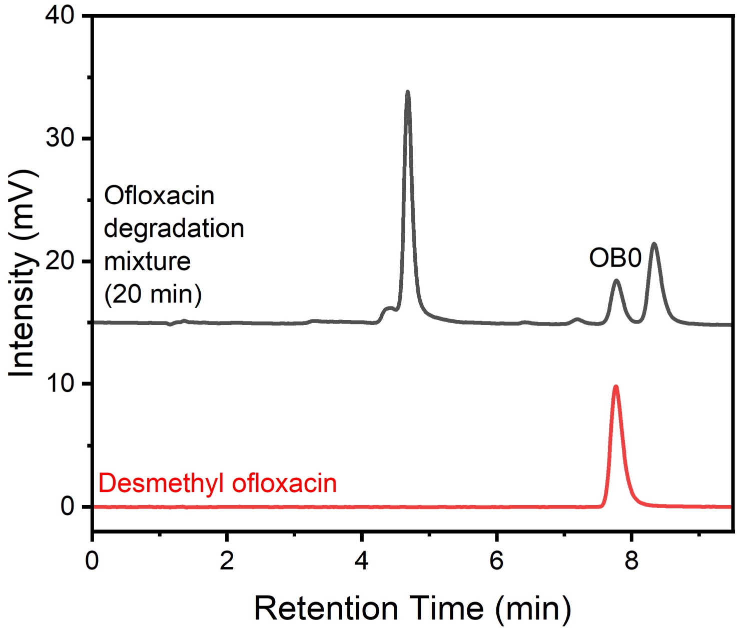

Figure S5. HPLC graphs of ofloxacin degradation products by **2**/H_2O_2 after 20 min (black) and a trace of desmethylofloxacin standard (red). Conditions: [ofloxacin] = 3×10^{-5} M, [**2**] = 1×10^{-7} M, [H_2O_2] = 1×10^{-3} M, pH 7.0 (0.01 M phosphate), 25 °C.

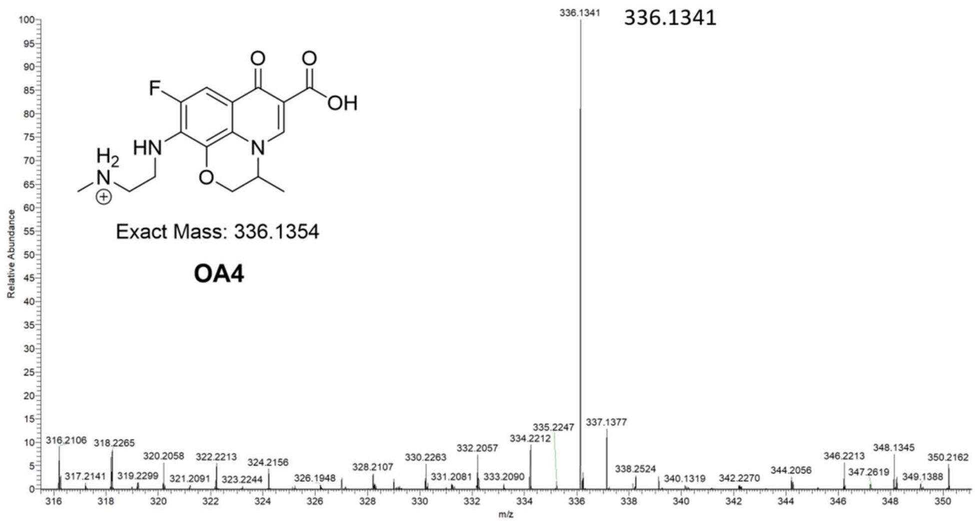

Figure S6. The HR-ESI-MS spectrum of product OA4.

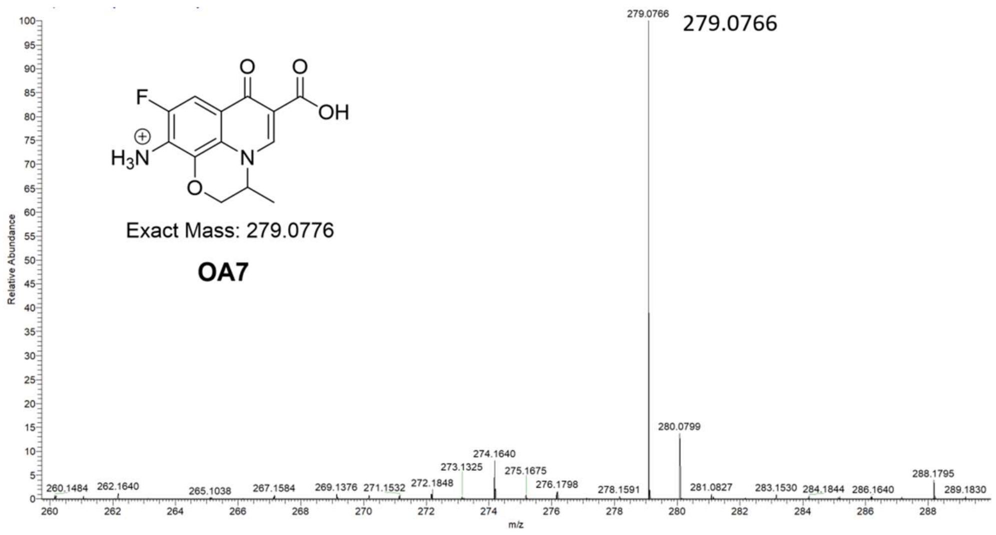

Figure S7. The HR-ESI-MS spectrum of product OA7.

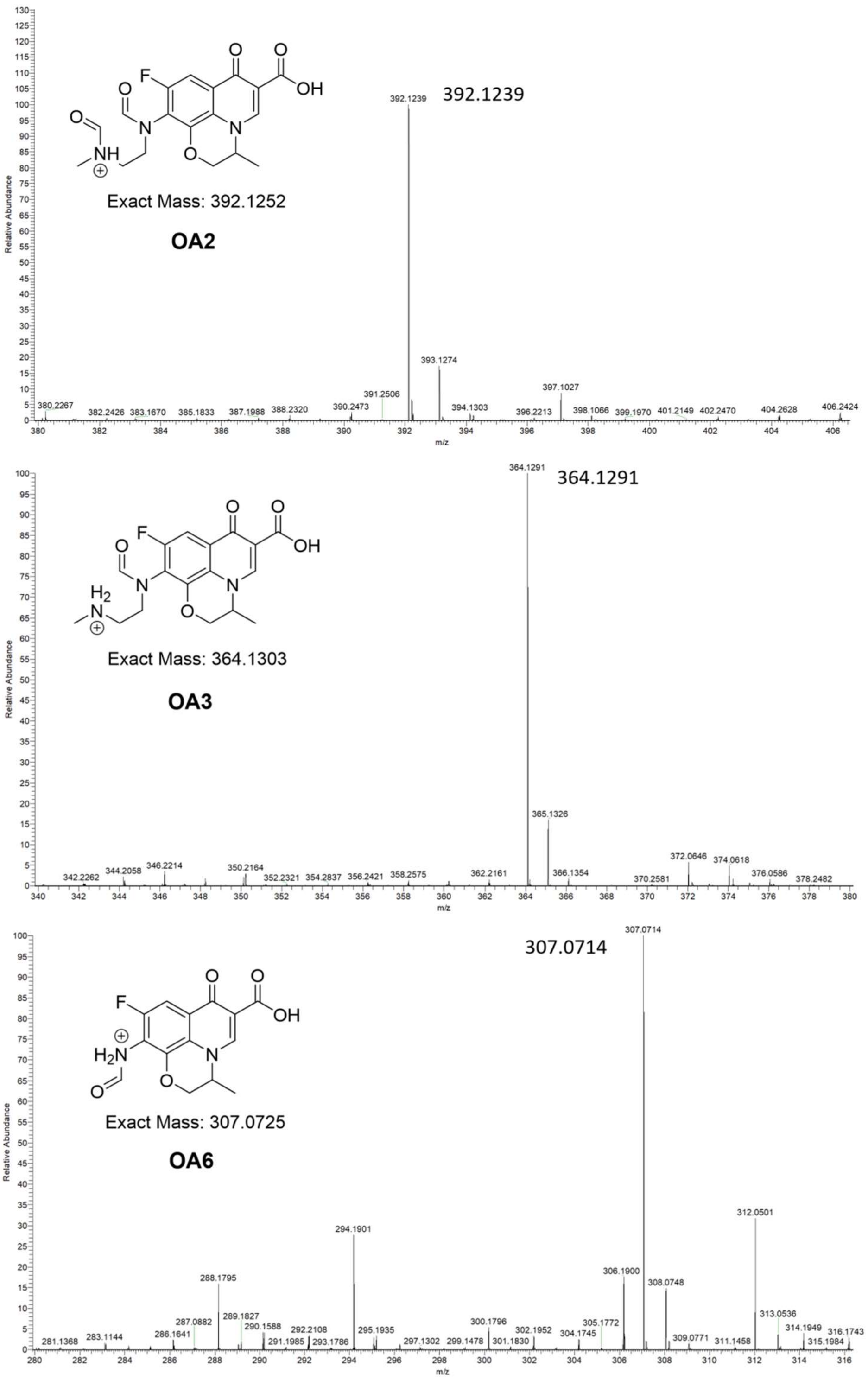

Figure S8. The HR-ESI-MS spectra of OA2 (Top), OA3 (Middle), OA6 (Bottom).

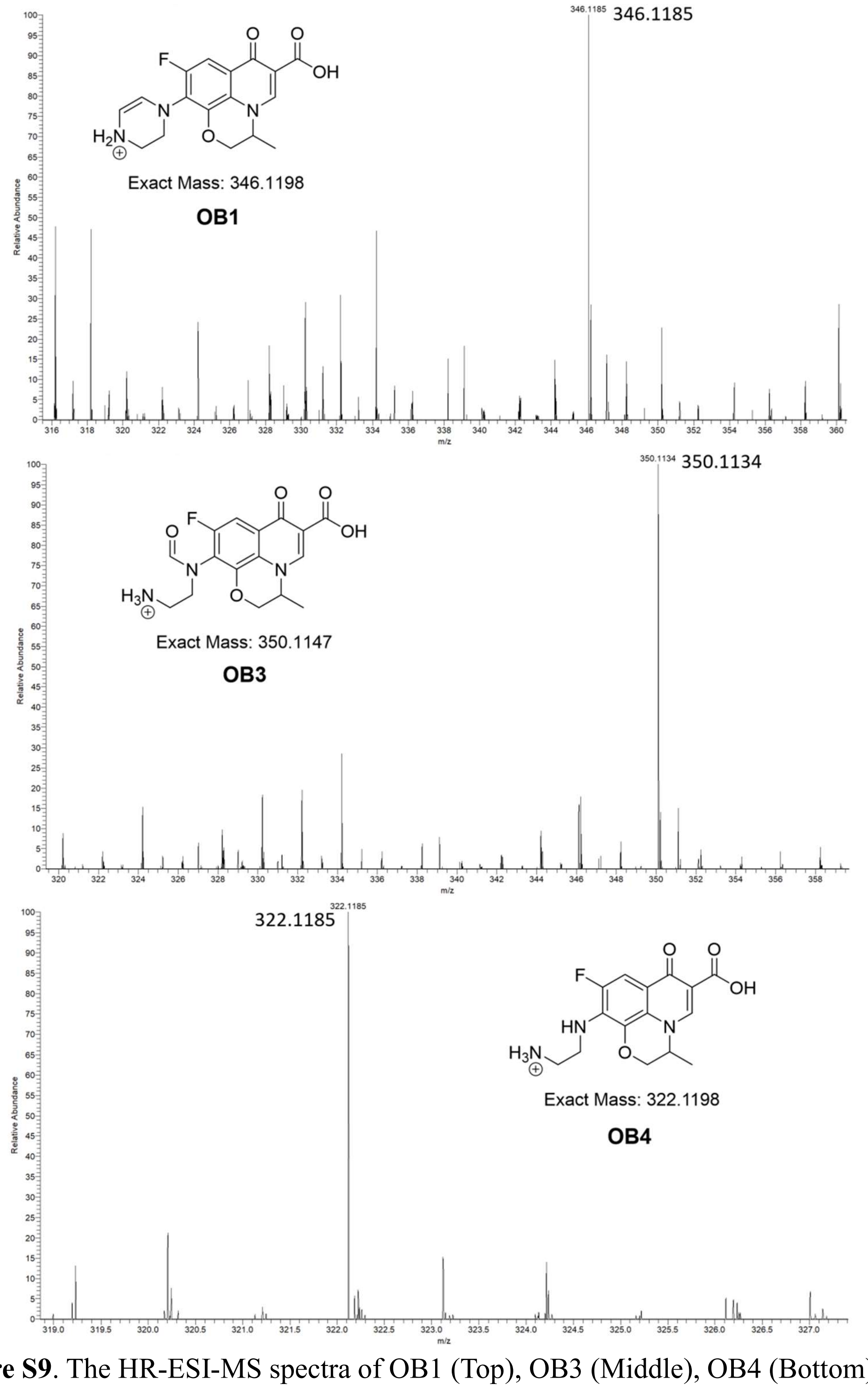

Figure S9. The HR-ESI-MS spectra of OB1 (Top), OB3 (Middle), OB4 (Bottom).

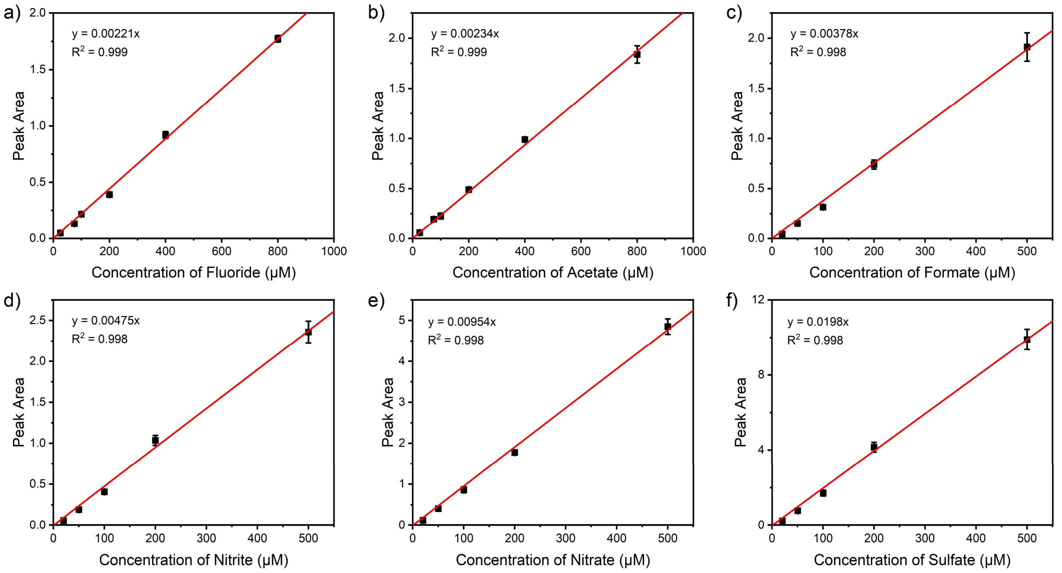

Figure S10. Calibration curves for a) fluoride, b) acetate, c) formate, d) nitrite, e) nitrate, f) sulfate in ion chromatogram analysis.

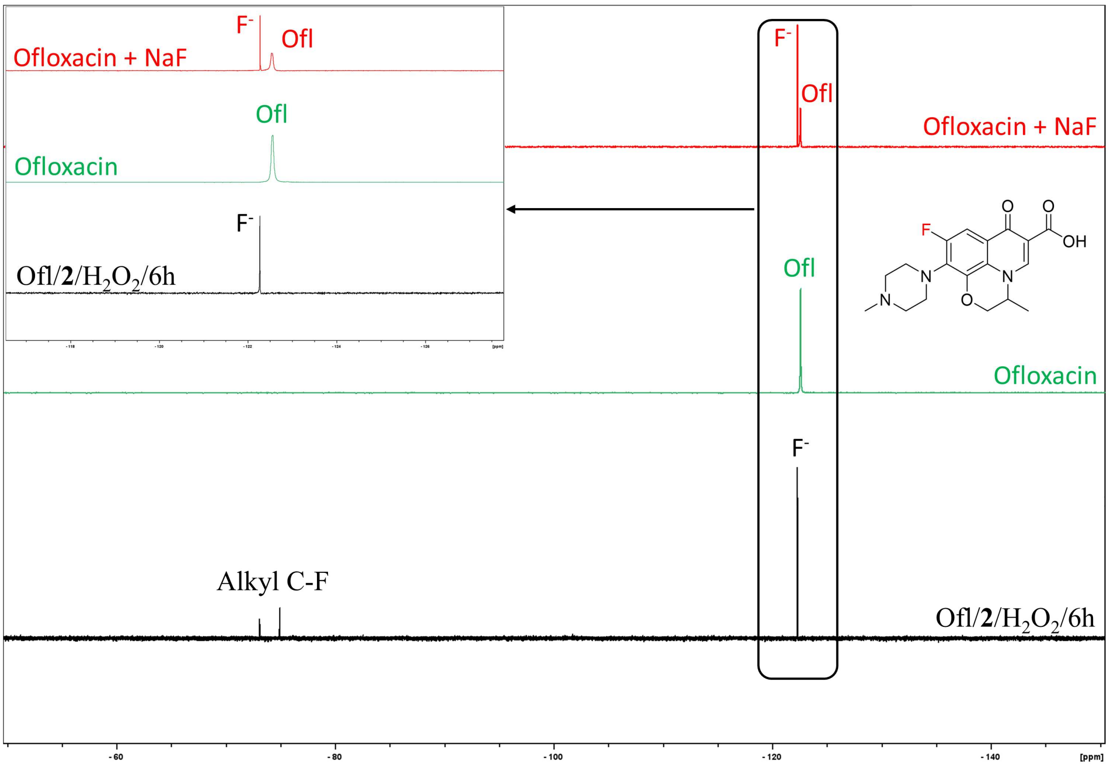

Figure S11. ^{19}F NMR spectra of ofloxacin + NaF (red), ofloxacin (green), and reaction mixture after 6h reaction (black). Initial conditions: [ofloxacin] = 2×10^{-5} M, [**2**] = 1×10^{-7} M (added at 0 and 3h), [H_2O_2] = 2×10^{-3} M, pH 7.0 (0.01 M phosphate), 25 °C, concentrated 50 times and redissolved in D_2O before NMR test.

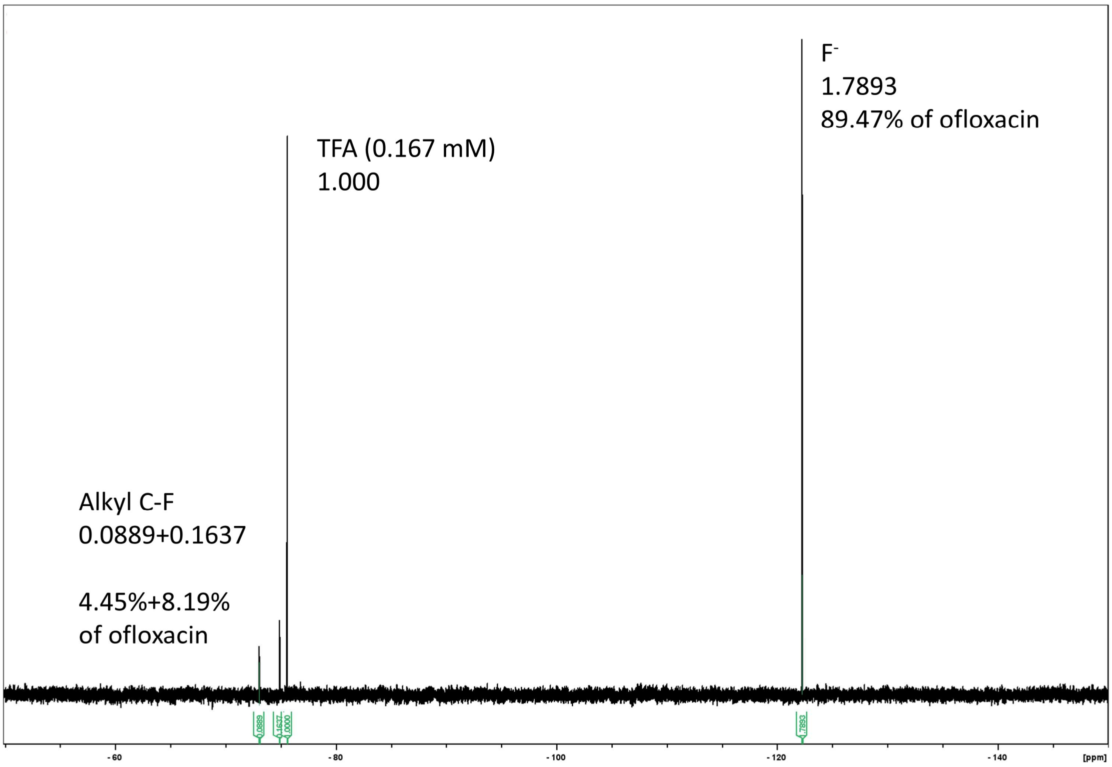

Figure S12. ^{19}F NMR spectra of final products after **2**/H_2O_2 treatment with the addition of 0.167 mM TFA as an internal standard. The numbers shown below the peak assignments represent the integral values of each resonance, normalized to 1 based on the integration of 0.167 mM TFA.

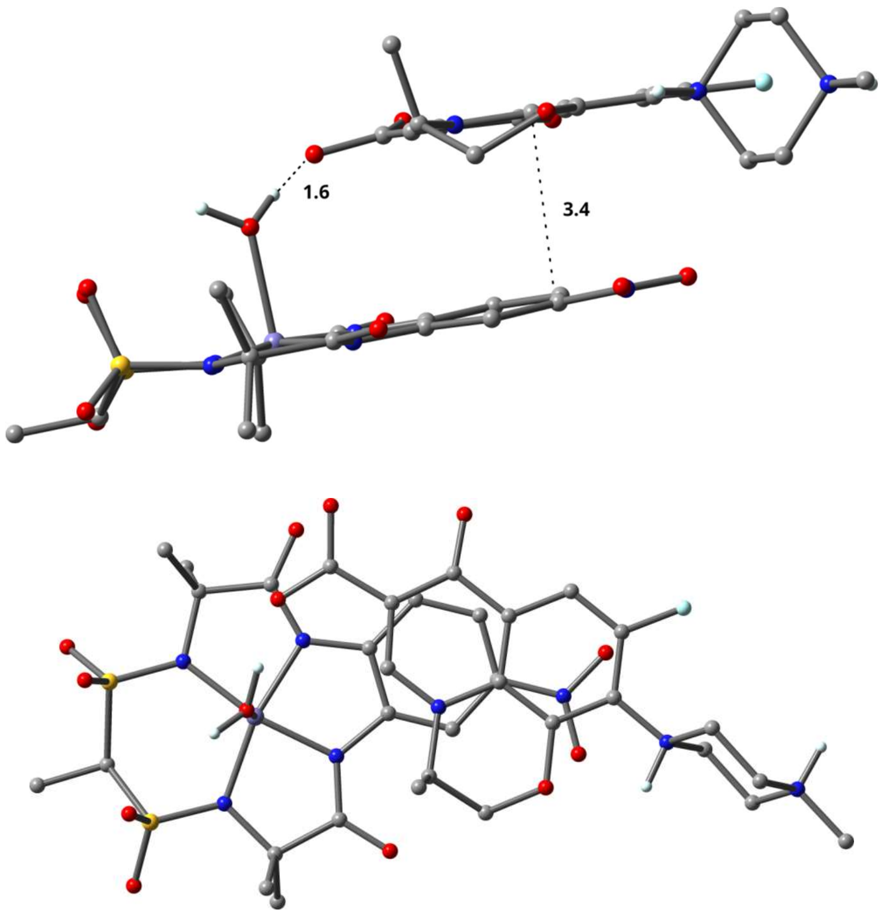

Figure S13. Side and top views of the DFT optimized structure of the non-covalent associate between **2** and ofloxacin featuring (*i*) a hydrophobic stacking contact between the quinolinone ring of ofloxacin and aromatic ring of **2** (the closest C···C contact is 3.4 Å) and (*ii*) a hydrogen bond (OH···O, 1.6 Å) between axial aqua ligand and the ofloxacin carboxylate as major stabilizing factors. The estimated free energy change for equilibrium 6 equals -12 kcal mol^{-1}. Only polar hydrogen atoms are shown.

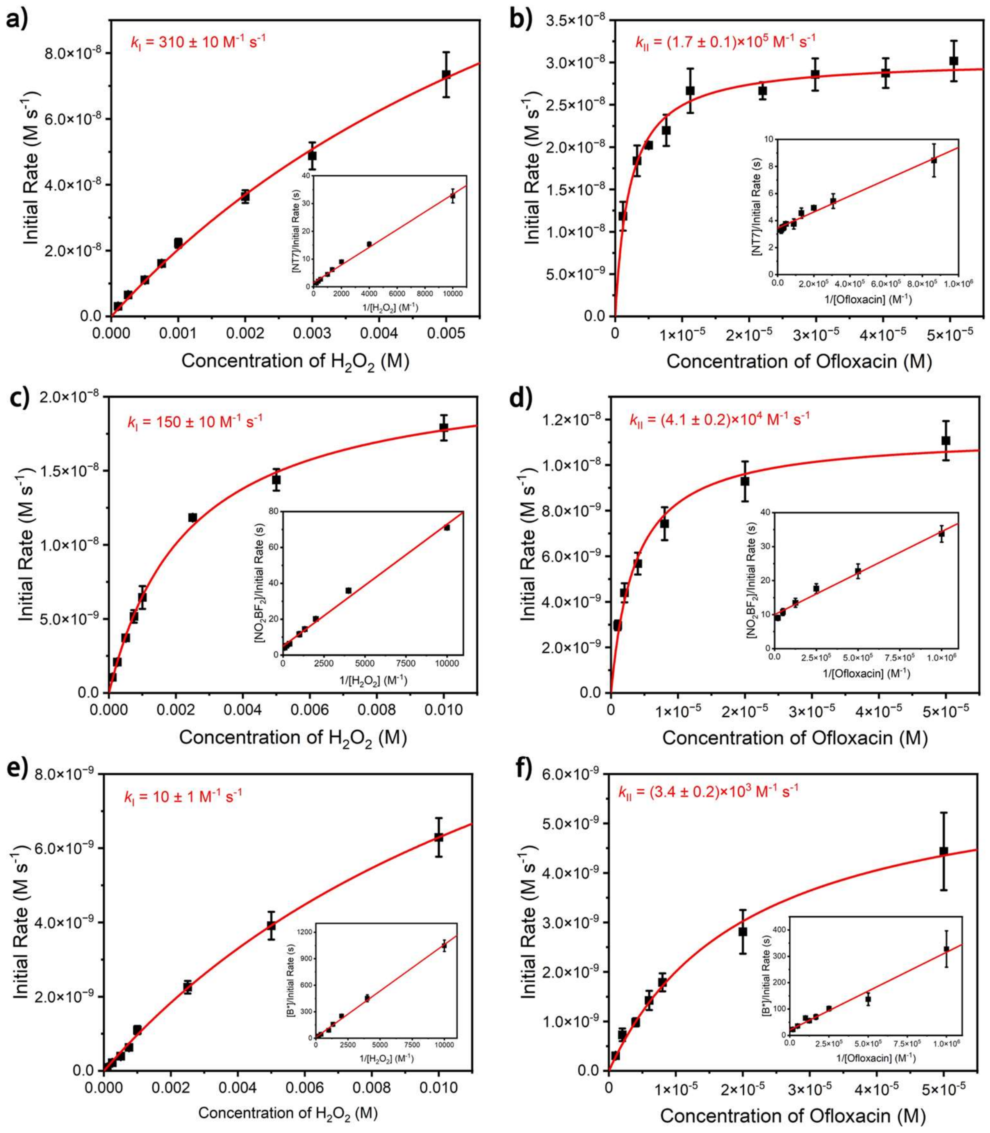

Figure S14. Initial rates of ofloxacin degradation by H_2O_2 catalyzed by TAML catalysts as a function of [H_2O_2] or [Ofloxacin] measured by HPLC. Inset shows the double inverse linear plot, [TAML]/(Initial rate) versus [H_2O_2]$^{-1}$ or [Ofloxacin]$^{-1}$. Conditions: pH 7.0 (0.01 M phosphate), 25 °C. Other concentrations are shown below.

Figure	[Ofloxacin]	[H_2O_2]	[TAML]
14a	2×10^{-5} M	-	**2** 1×10^{-7} M
14b	-	1×10^{-3} M	**2** 1×10^{-7} M
14c	2×10^{-5} M	-	**1b** 1×10^{-7} M
14d	-	1×10^{-3} M	**1b** 1×10^{-7} M
14e	2×10^{-5} M	-	**1a** 1×10^{-7} M
14f	-	4×10^{-3} M	**1a** 1×10^{-7} M

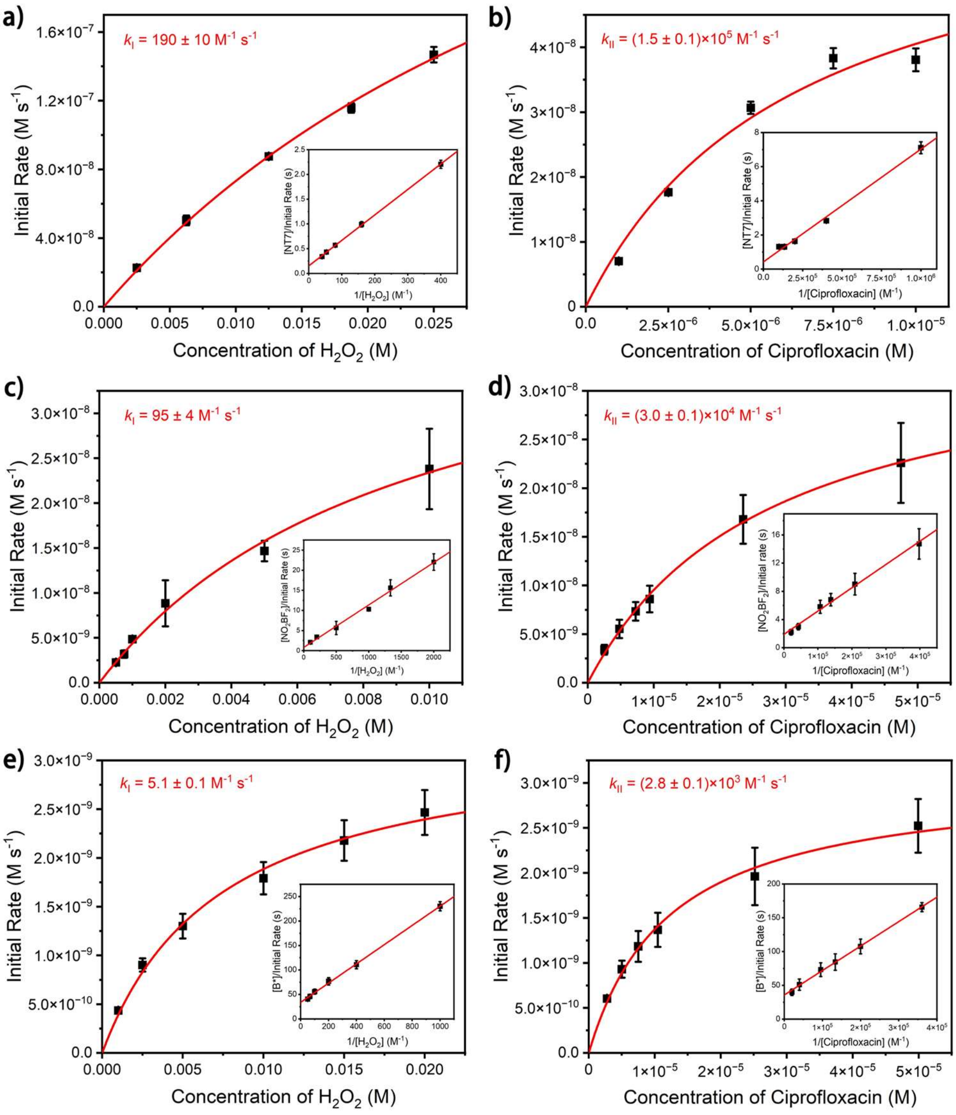

Figure S15. Initial rates of ciprofloxacin degradation by H_2O_2 catalyzed by TAMLs as a function of [H_2O_2] or [Ciprofloxacin] measured by HPLC. Inset shows the double inverse linear plot, [TAML]/(Initial rate) versus $[H_2O_2]^{-1}$ or $[\text{Ciprofloxacin}]^{-1}$. Conditions: pH 7.0 (0.01 M phosphate), 25 °C. Other concentrations are listed below.

Figure	[Ciprofloxacin]	[H_2O_2]	[TAML]
15a	7.5×10^{-5} M	-	**2** 5×10^{-8} M
15b	-	1×10^{-2} M	**2** 5×10^{-8} M
15c	7.5×10^{-5} M	-	**1b** 5×10^{-8} M
15d	-	1×10^{-2} M	**1b** 5×10^{-8} M
15e	7.5×10^{-5} M	-	**1a** 1×10^{-7} M
15f	-	2×10^{-2} M	**1a** 1×10^{-7} M

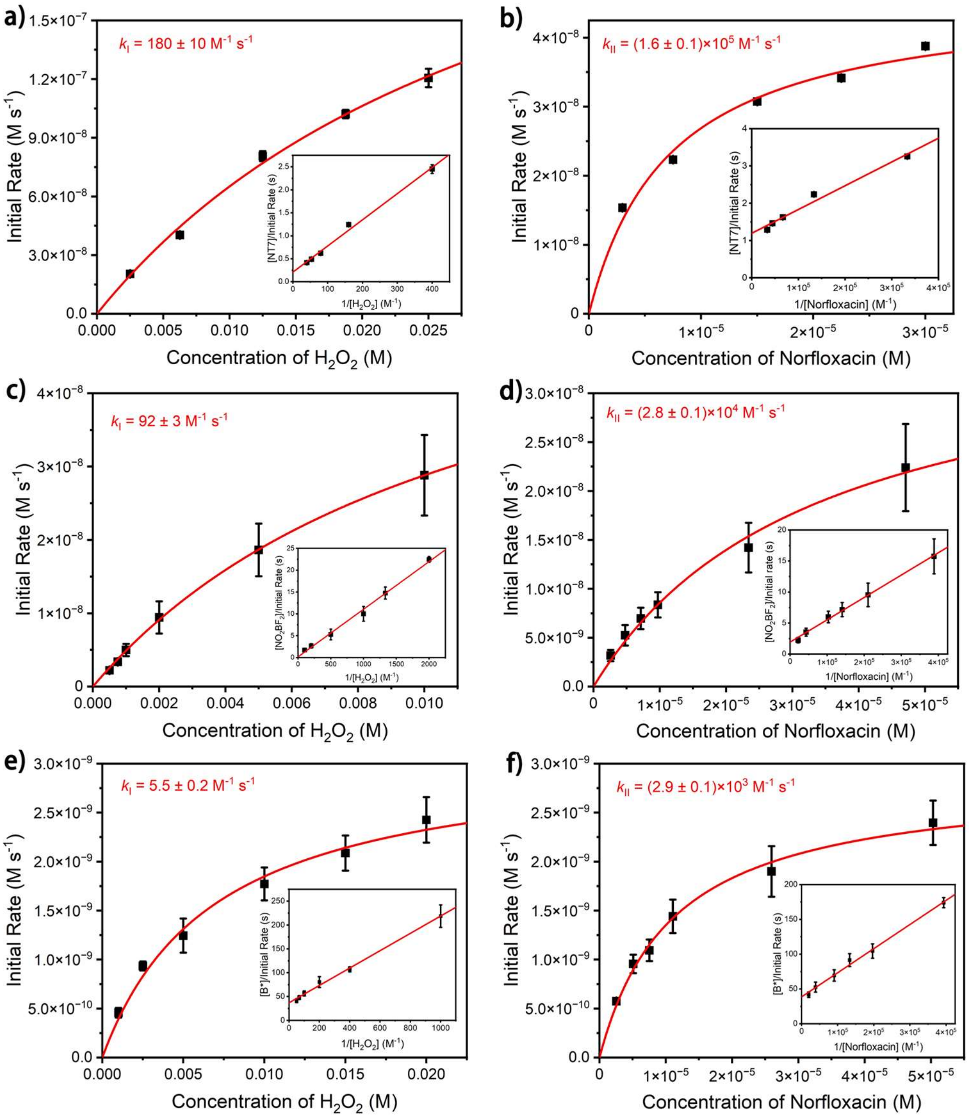

Figure S16. Initial rates of norfloxacin degradation by H_2O_2 catalyzed by TAML catalysts as a function of [H_2O_2] or [Norfloxacin] measured by HPLC. Inset shows the double inverse linear plot, [TAML]/(Initial rate) versus [H_2O_2]$^{-1}$ or [Norfloxacin]$^{-1}$. Conditions: pH 7.0 (0.01 M phosphate), 25 °C. Other concentrations are listed below.

Figure	[Norfloxacin]	[H_2O_2]	[TAML]
16a	7.5×10^{-5} M	-	**2** 5×10^{-8} M
16b	-	5×10^{-3} M	**2** 5×10^{-8} M
16c	7.5×10^{-5} M	-	**1b** 5×10^{-8} M
16d	-	1×10^{-2} M	**1b** 5×10^{-8} M
16e	7.5×10^{-5} M	-	**1a** 1×10^{-7} M
16f	-	2×10^{-2} M	**1a** 1×10^{-7} M

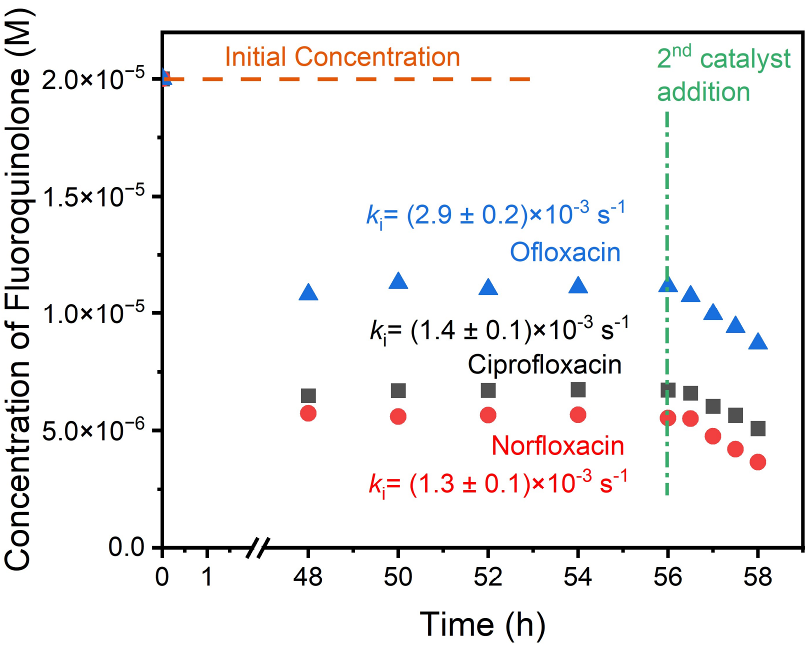

Figure S17. Concentration versus time profiles for the incomplete degradation of three FQs by H_2O_2 catalyzed by **2**. The dotted horizontal orange line indicates the initial concentration of the FQs. The dotted vertical green line indicates a second addition of **2**. Conditions: [fluoroquinolone] = 2×10^{-5} M, [**2**] = 1×10^{-8} M (each addition), [H_2O_2] = 1×10^{-3} M, pH 7.0 (0.01 M phosphate), 25 °C.

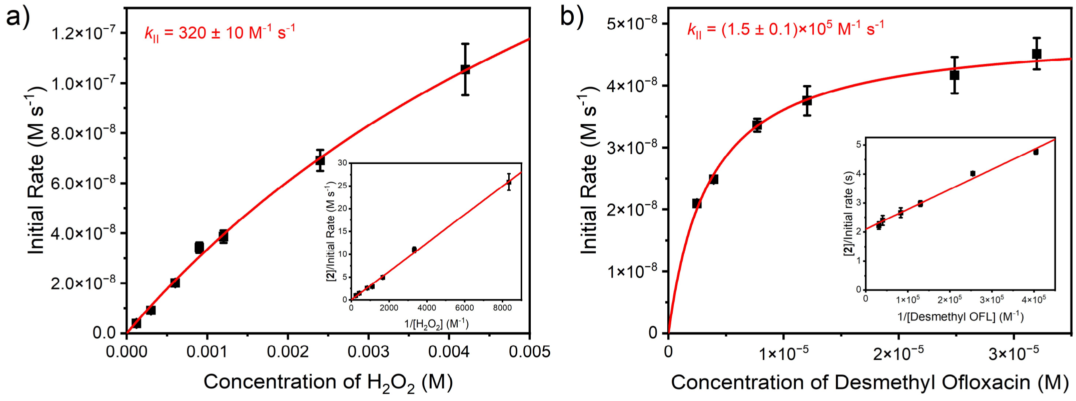

Figure S18. Initial rate of desmethylofloxacin degradation by H_2O_2 catalyzed by **2** as a function of a) [H_2O_2] or b) [Desmethylofloxacin] measured by HPLC. Inset shows the double inverse linear plot, [**2**]/(Initial rate) versus a) [H_2O_2]$^{-1}$ or b) [Desmethylofloxacin]$^{-1}$. Conditions: [Desmethylofloxacin] = 2×10^{-5} M, [**2**] = 1×10^{-7} M, [H_2O_2] = 1×10^{-3} M, pH 7.0 (0.01 M phosphate), 25 °C.

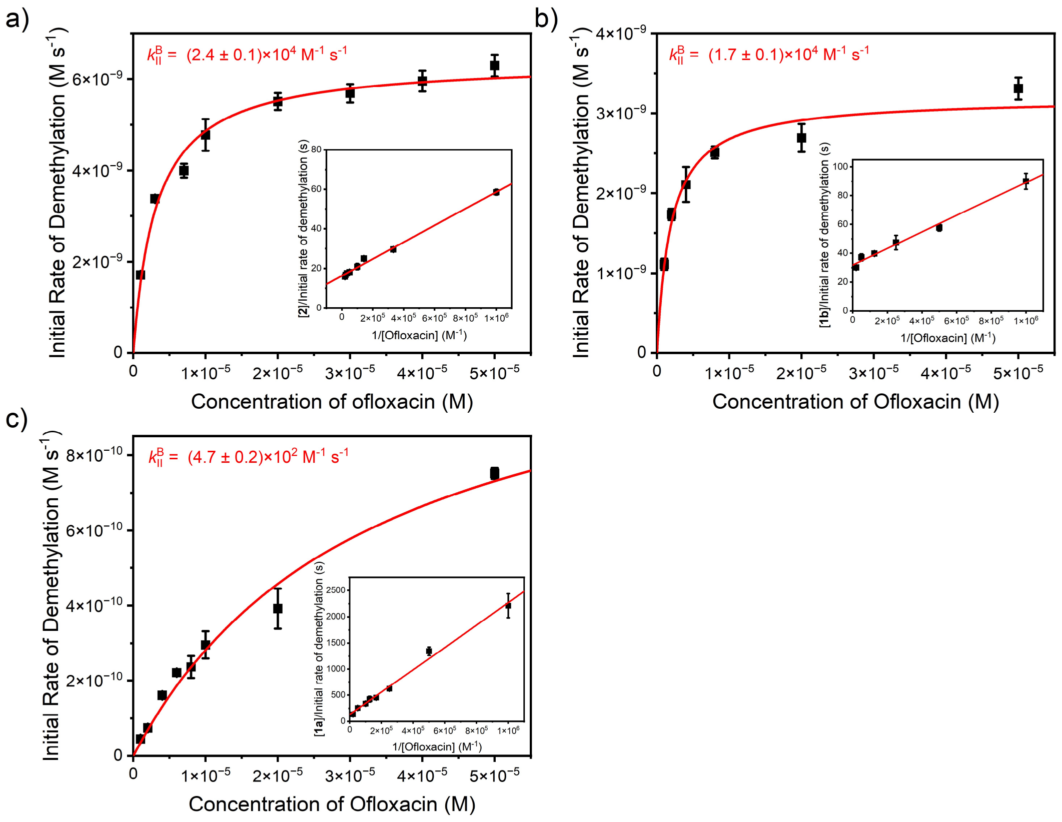

Figure S19. Initial rates of ofloxacin demethylation by H_2O_2 catalyzed by TAML catalysts as a function of [Ofloxacin] measured by HPLC. Inset shows the double inverse linear plot, [TAML]/(Initial rate) versus [Ofloxacin]$^{-1}$. Conditions: pH 7.0 (0.01 M phosphate), 25 °C. Other concentrations are listed in the following table:

Figure	[Ofloxacin]	[H_2O_2]	[TAML]
19a	-	1×10^{-3} M	**2** 1×10^{-7} M
19b	-	1×10^{-3} M	**1b** 1×10^{-7} M
19c	-	4×10^{-3} M	**1a** 1×10^{-7} M

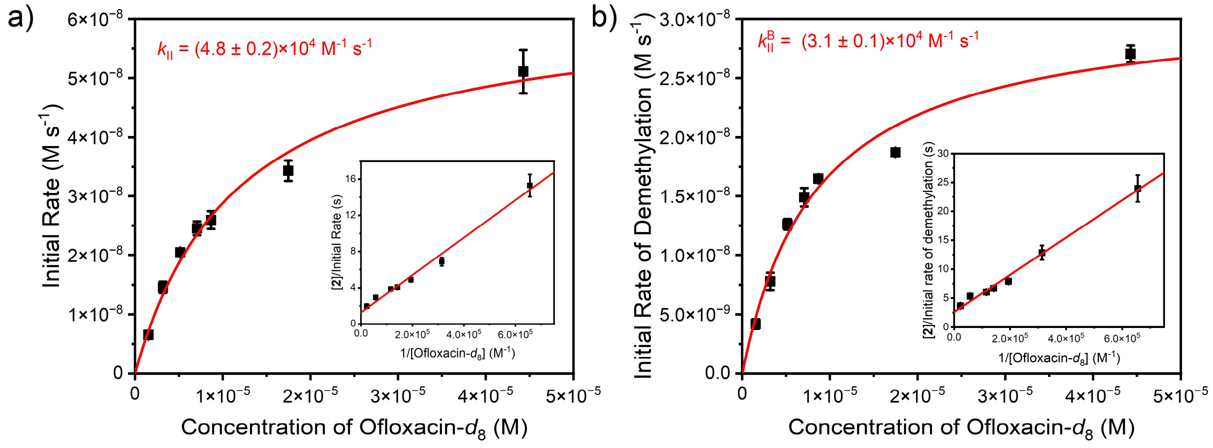

Figure S20. Initial rate of a) ofloxacin-d_8 degradation b) ofloxacin-d_8 demethylation by H_2O_2 catalyzed by **2** as a function of [ofloxacin-d_8] measured by HPLC. Inset shows the double inverse linear plot, [**2**]/(Initial rate) versus [ofloxacin-d_8]$^{-1}$. Conditions: [**2**] = 1×10^{-7} M, [H_2O_2] = 1×10^{-3} M, pH 7.0 (0.01 M phosphate), 25 °C.

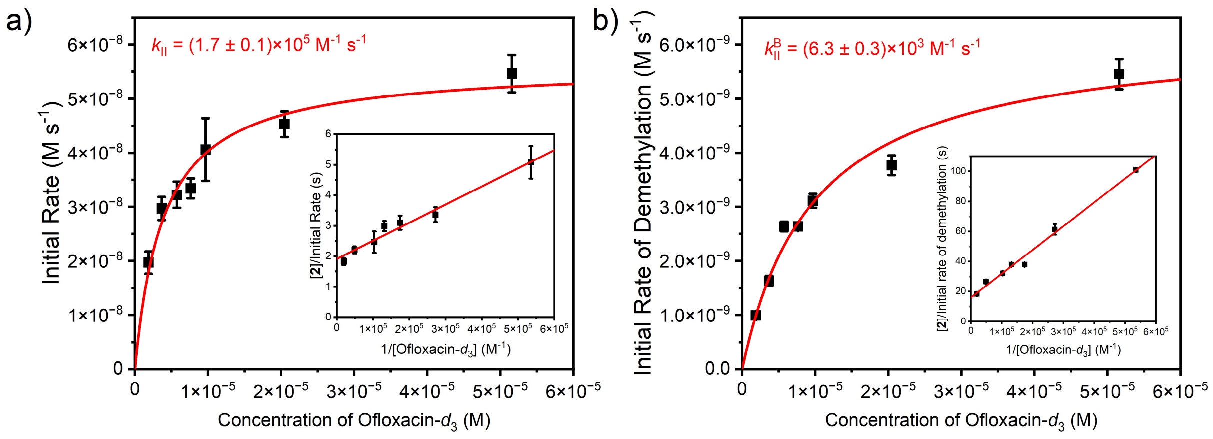

Figure S21. Initial rate of a) ofloxacin-d_3 degradation b) ofloxacin-d_3 demethylation by H_2O_2 catalyzed by **2** as a function of [ofloxacin-d_3] measured by HPLC. Inset shows the double inverse linear plot, [**2**]/(Initial rate) versus [ofloxacin-d_3]$^{-1}$. Conditions: [**2**] = 1×10^{-7} M, [H_2O_2] = 1×10^{-3} M, pH 7.0 (0.01 M phosphate), 25 °C.

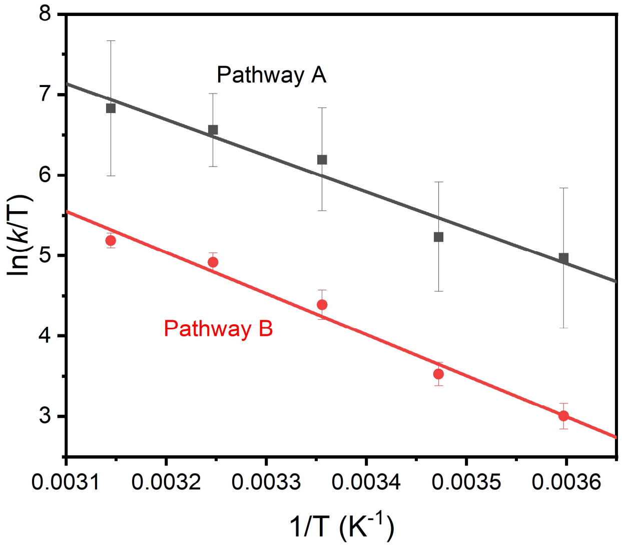

Figure S22. Calculation of the activation parameters for the desaturation (k_{II}^{A}, Pathway A) and *N*-demethylation (k_{II}^{B}, Pathway B) for ofloxacin (**2**/H_2O_2) in water at pH 7 (0.01 M phosphate).

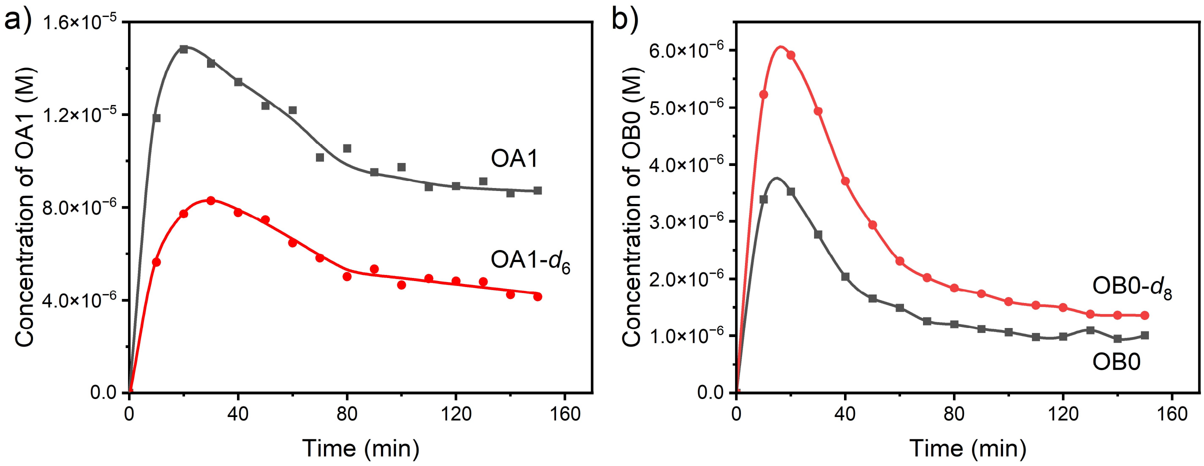

Figure S23. Concentration versus time profiles for OA1 and OB0 during the degradation of ofloxacin and ofloxacin-d_8 by **2**/H_2O_2. Conditions: [ofloxacin] = 3×10^{-5} M, [**2**] = 1×10^{-7} M, [H_2O_2] = 1×10^{-3} M pH 7.0 (0.01 M phosphate), 25 °C.

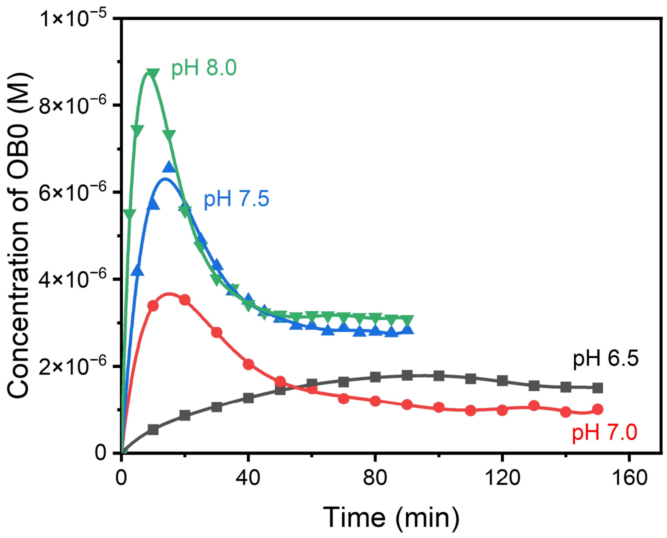

Figure S24. Concentration versus time profiles for OB0 during the degradation of ofloxacin by **2**/H_2O_2 under different pH. Conditions: [ofloxacin] = 3×10^{-5} M, [**2**] = 1×10^{-7} M, [H_2O_2] = 1×10^{-3} M, 25 °C.

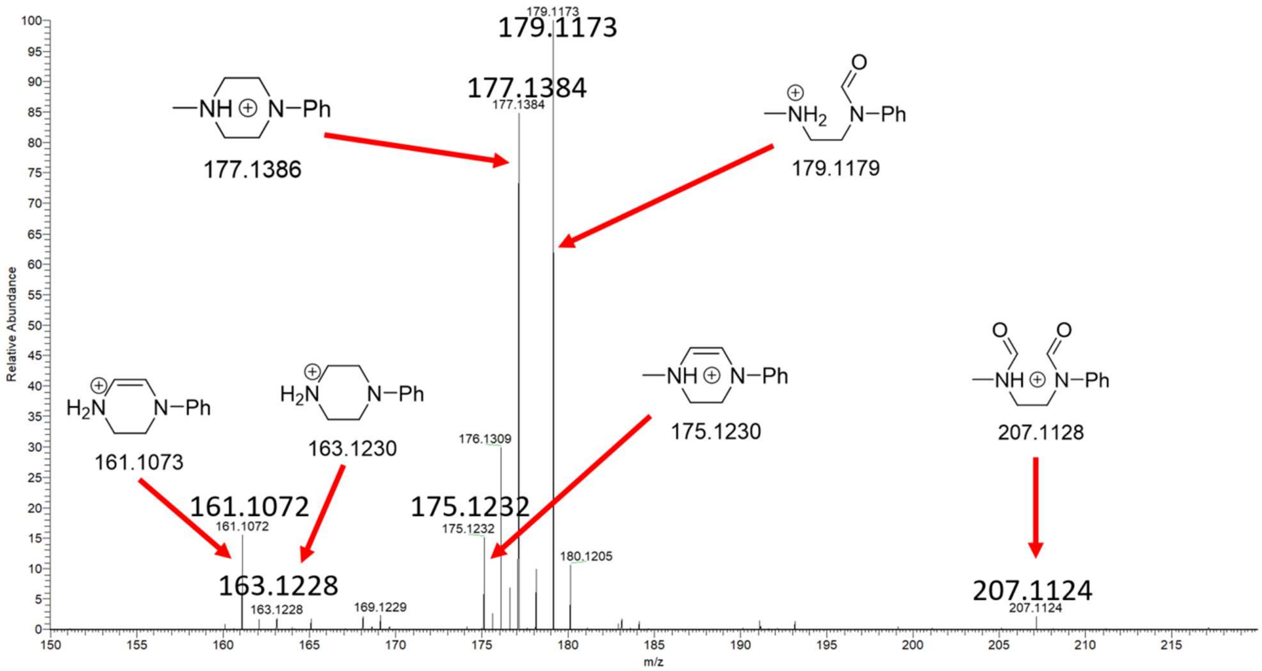

Figure S25. The HR-ESI-MS spectrum of the reaction mixture after degradation of 1-methyl-4-phenylpiperazine by **2**/H_2O_2. Conditions: [1-methyl-4-phenylpiperazine] = 1×10^{-4} M, [**2**] = 2×10^{-7} M, [H_2O_2] = 5×10^{-3} M, H_2O, 25 °C. Reaction time: 20 min. Numbers under structures are exact masses.

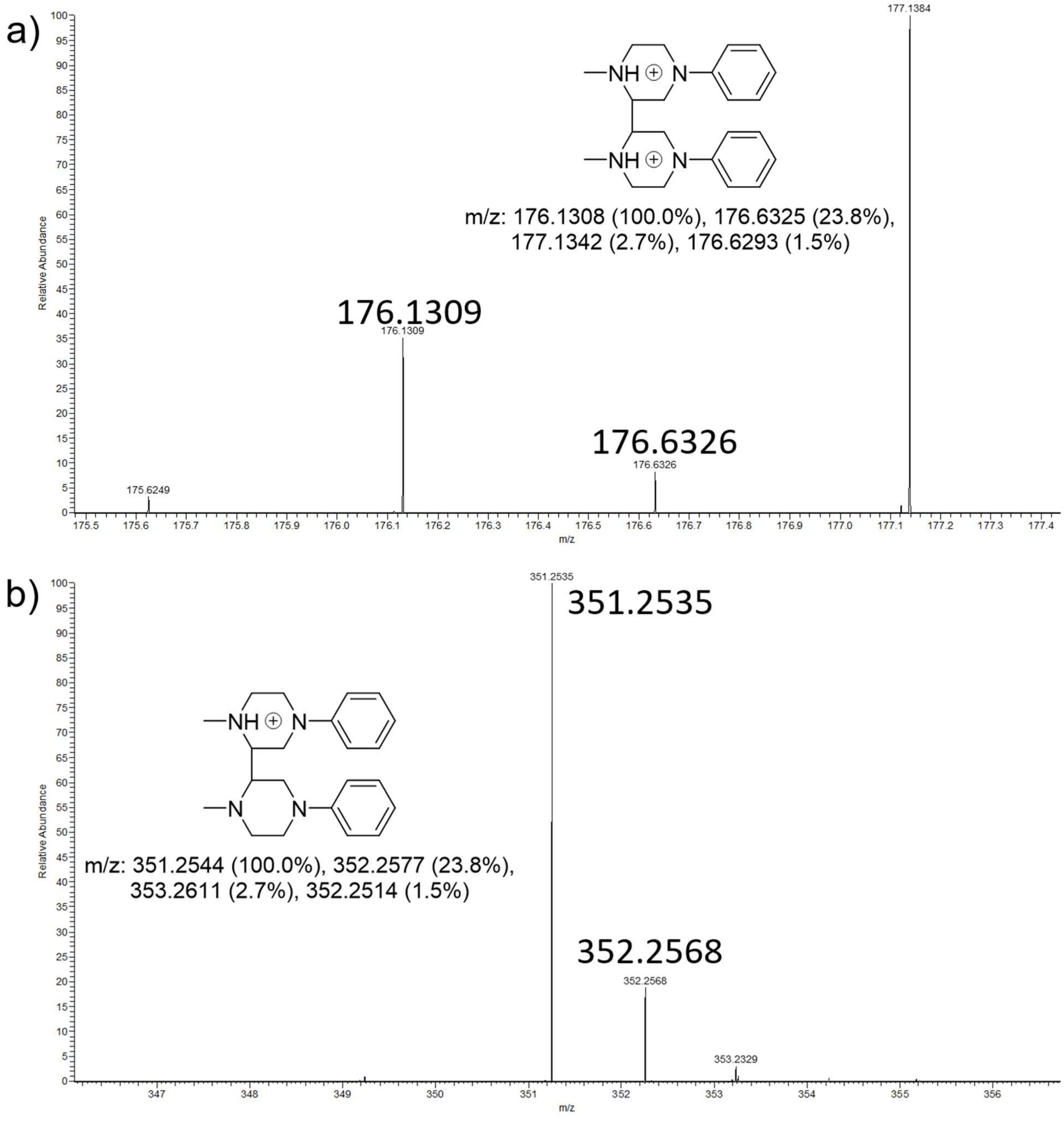

Figure S26. The HR-ESI-MS spectra of a) $[M+2H]^{2+}$ peaks and b) $[M+H]^{+}$ peaks for 1-methyl-4-phenylpiperazine dimer.

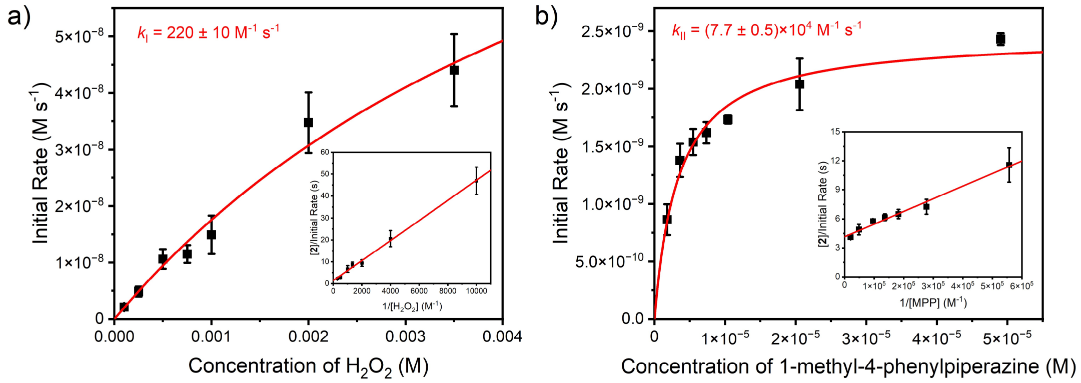

Figure S27. Initial rate of the **2**/H_2O_2 degradation of 1-methyl-4-phenylpiperazine as a function of a) [H_2O_2] or, b) [1-methyl-4-phenylpiperazine] as measured by HPLC. Inset shows the double inverse linear plot, [**2**]/(Initial rate) versus $[H_2O_2]^{-1}$ or [1-methyl-4-phenylpiperazine]$^{-1}$. Conditions when a reagent's concentration is not being systematically varied: [1-methyl-4-phenylpiperazine] = 2×10^{-5} M, [**2**] = 1×10^{-7} M, [H_2O_2] = 1×10^{-3} M, pH 7.0 (0.01 M phosphate), 25 °C.

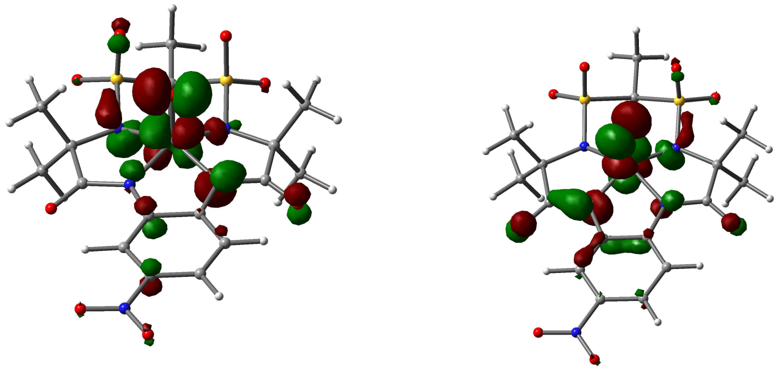

(aHOMO) (aLUMO)

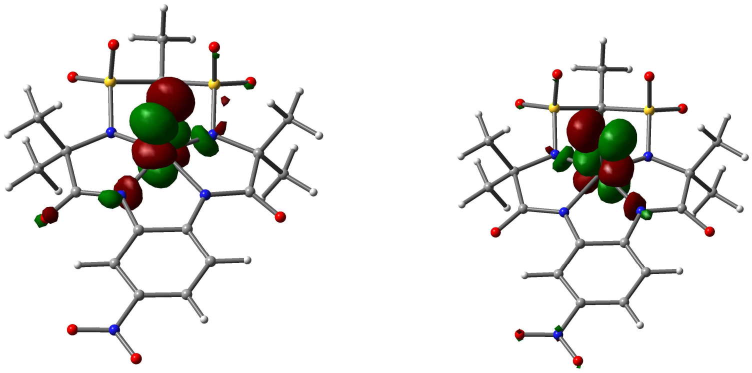

(bLUMO) (bLUMO+1)

Figure S28. Selected molecular orbital contours for the [(L•$^+$)Fe(IV)(O)]$^-$.

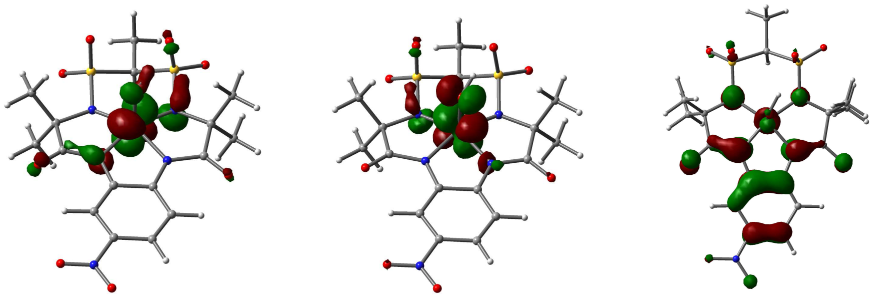

(bLUMO) (bLUMO+1) (aLUMO)

Figure S29. Selected molecular orbital contours for (L•$^+$)Fe(IV)(OH).

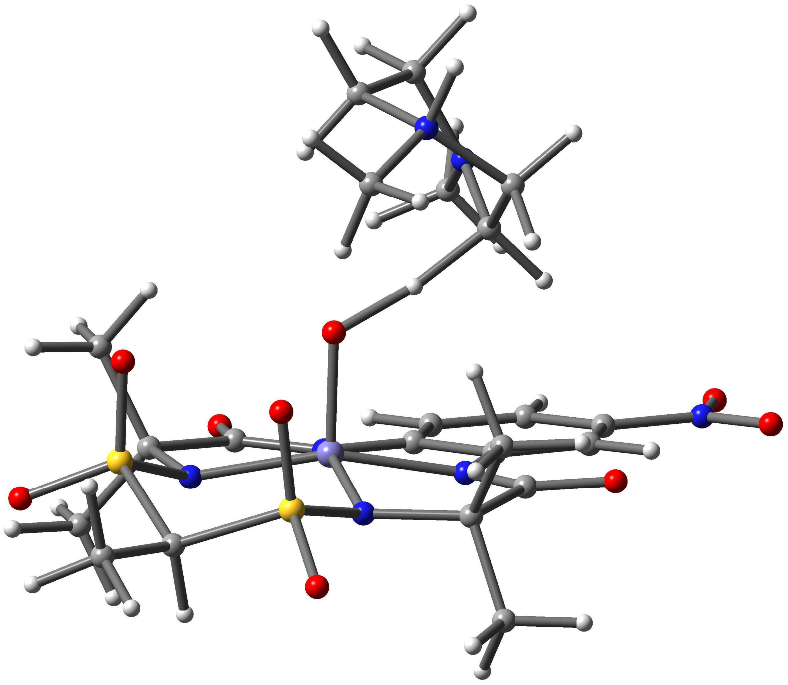

Figure S30. Transition state structure from DFT for abstracting a hydrogen atom from 1,4-dimethylpiperazine by [(L•+)Fe(IV)(O)]$^{-}$. Atoms are color coded: O, red; N, blue, S, yellow; C, grey; H, white. Key bond lengths in angstrom. Fe-N(neighboring): 1.94 and 1.95 to N(S) and 19.0 and 1.91 to N(C). Fe-O(axial): 1.67. O(axial)-H(transferring): 1.28. C(1,4-dimethylpiperazine)-H(transferring): 1.31.

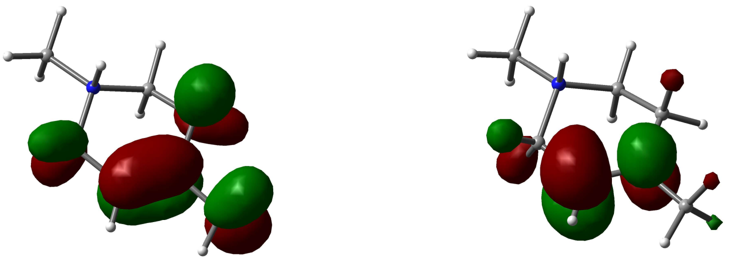

(HOMO) (LUMO)

Figure S31. Molecular orbital contours from DFT for the iminium species proposed to form in the **2**/H_2O_2 oxidation of 1,4-dimethylpiperazine. The in-ring C-N bonds distances for the reduced and oxidized carbons bonded to the iminium-N are 1.5 and 1.3 angstroms, respectively.

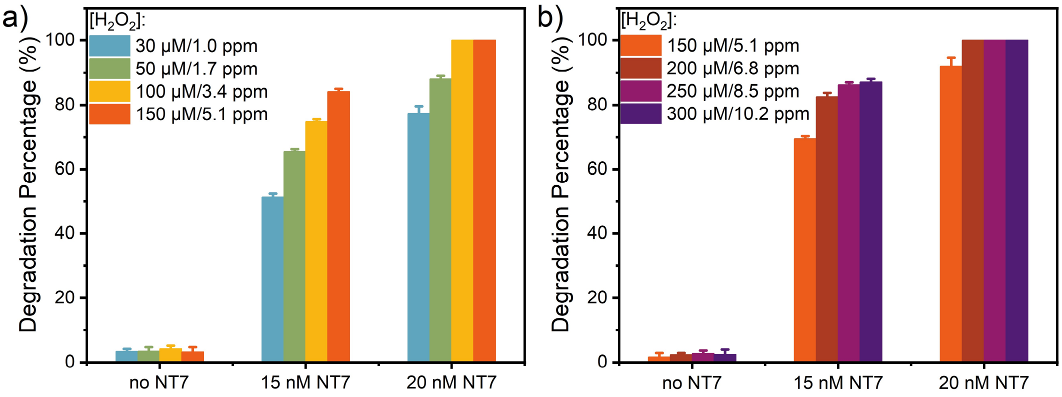

Figure S32. Ultradilute oxidation by **2**/H_2O_2 of a fluoroquinolone mixture (5×10^{-8} M each of ofloxacin, ciprofloxacin and norfloxacin) in a) secondary effluent (6 hours), b) primary effluent (12 hours). Note: for H_2O_2, 1 ppm equals 29.4 μM ≈ 30 μM.

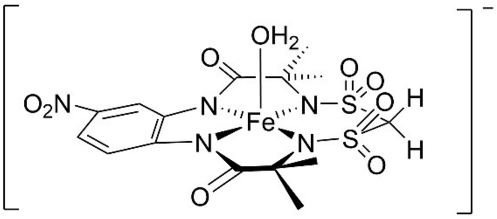

3

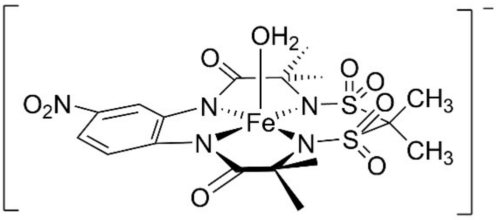

4

Chart S1. The structures of NewTAMLs used for benchmarking the DFT method. The crystal structure of **3** can be found in literature[1] and the crystal structure of **4** is currently unpublished.

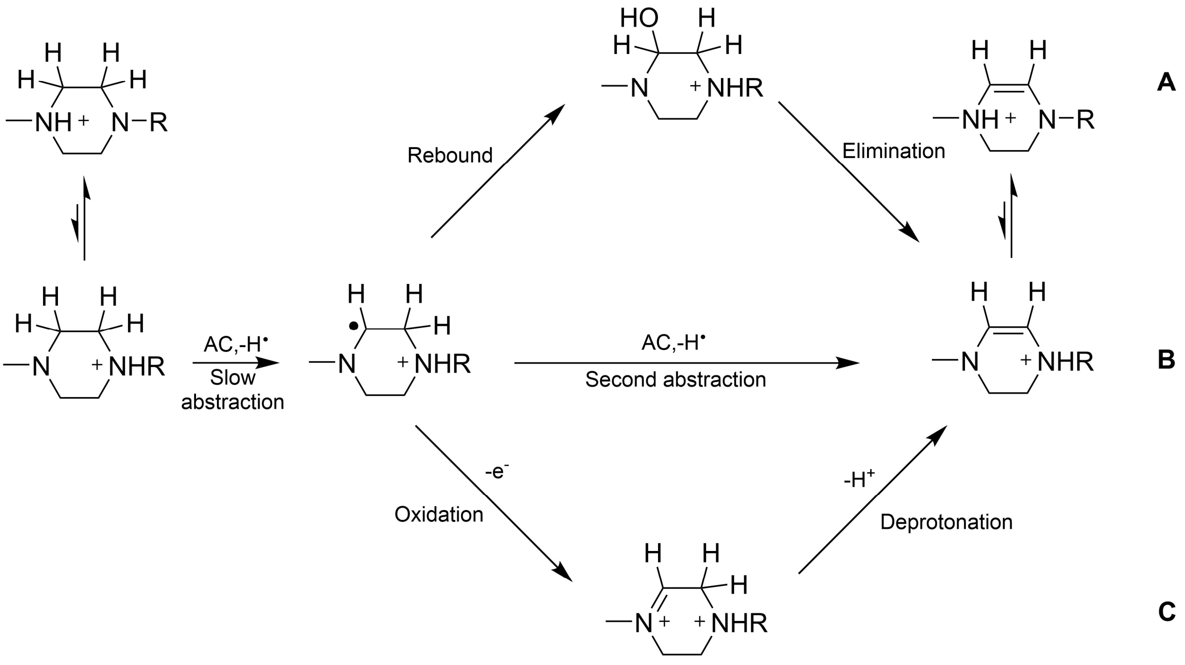

Scheme S1. Potential pathways for the TAML/H_2O_2 desaturase activity following rate-limiting *sp*^{3} C−H abstraction by the activated species (initial abstraction on the carbon alpha to the *N*-methyl): (A) rebound-elimination, (B) 2nd radical abstraction and, (C) oxidation/deprotonations.

Table S1. HPLC conditions for different substrates used in this study. Mobile phase A: phosphate buffer (0.01 M, pH 3.0); mobile phase B: MeCN; flow rate 1 mL min^{-1}.

Chemical	Ratio (A:B)	Detection wavelength (nm)
Ofloxacin	92:8	287
Ciprofloxacin	88:12	278
Norfloxacin	90:10	278
1-Methyl-4-phenylpiperazine	92:8	238

Table S2. The LC-MS chromatographic methods for different substrates.

Chemical	Flow rate (mL/min)	Mobile phase A	Mobile phase B	Ratio-A:B (time)
Ofloxacin	0.3	H_2O + 0.1% HCOOH	MeOH + 0.1% HCOOH	90:10 (0 min)<br>90:10 (2 min)<br>10:90 (7 min)<br>90:10 (11 min)<br>90:10 (13 min)
Ciprofloxacin	0.2	H_2O + 0.1% HCOOH	CH_3CN + 0.1% HCOOH	88:12 (0-13 min)
Norfloxacin	0.2	H_2O + 0.1% HCOOH	CH_3CN + 0.1% HCOOH	90:10 (0-13 min)

Table S3. Root mean square errors (rmsd) for varied computational methods against two NewTAML crystal structures (Chart S1). The numerical quantities bear the physical unit of angstrom and were obtained using the open-source version of the pymol software. (The PyMOL Molecular Graphics System, Version 3.0 Schrödinger, LLC.)

Methods	NewTAML **3**	NewTAML **4**
M06L[2]/6-311+G(d)	0.19	0.07
M06[3]/6-311+G(d)	0.20	0.09
B3LYP[4–6]_D3BJ[7]/6-311+G(d)	0.22	0.09
TPSSh[8,9]_D3BJ[7]/6-311+G(d)	0.22	0.08
wB97XD/6-311+G(d)	0.22	0.09

Table S4. Rate constants $k_{\mathrm{II}}^{\mathrm{A}}$ and $k_{\mathrm{II}}^{\mathrm{B}}$ (in M^{-1} s^{-1}) for the **2**-catalyzed degradation of ofloxacin by H_2O_2 at pH 7 under different temperatures. Temperatures at 278 and 288 were controlled by HPLC autosampler, temperatures at 308 and 318 K were controlled by water bath.

T / K	$10^{-3} \times k_{\mathrm{II}}^{\mathrm{A}}$ [a)]	$10^{-3} \times k_{\mathrm{II}}^{\mathrm{B}}$ [b)]
278	40 ± 7	5.6 ± 0.3
288	54 ± 7	9.8 ± 0.4
298	146 ± 15	24 ± 1
308	218 ± 15	42 ± 1
318	293 ± 36	57 ± 1

[a)] $\Delta H^{\neq} = 37 \pm 5$ kJ mol^{-1}; $\Delta S^{\neq} = -23 \pm 2$ J K^{-1} mol^{-1};
[b)] $\Delta H^{\neq} = 43 \pm 4$ kJ mol^{-1}; $\Delta S^{\neq} = -20 \pm 1$ J K^{-1} mol^{-1}.

Table S5. Reaction barriers for the different considered Activated Catalyst (AC) intermediates in the **2**-catalyzed HAT from mono- and di-protonated 1,4-dimethylpiperazine and the comparative estimated rate constants for the different processes; L is TAML ligand.

AC	TAML Oxidized State	Barrier / kJ mol^{-1}	k_{II} / M^{-1} s^{-1}	Barrier / kJ mol^{-1}	k_{II} / M^{-1} s^{-1}
		1,4-dimethylpiperazine-2H$^+$		1,4-dimethylpiperazine-H$^+$	
[**2**-O]$^{2-}$	LFe(IV)	340	Negligible	452	Negligible
[**2**-OH]$^-$	LFe(IV)	unattained	Negligible	93	3.8×10^{-4}
[**2**-O]$^-$	(L•$^+$)Fe(IV)	129	Negligible	29	5×10^{7}
[**2**-OH]	(L•$^+$)Fe(IV)	888	Negligible	47	3.6×10^{4}

Reference

(1) Warner, G. R.; Somasundar, Y.; Jansen, K. C.; Kaaret, E. Z.; Weng, C.; Burton, A. E.; Mills, M. R.; Shen, L. Q.; Ryabov, A. D.; Pros, G.; Pintauer, T.; Biswas, S.; Hendrich, M. P.; Taylor, J. A.; Vom Saal, F. S.; Collins, T. J. Bioinspired, Multidisciplinary, Iterative Catalyst Design Creates the Highest Performance Peroxidase Mimics and the Field of Sustainable Ultradilute Oxidation Catalysis (SUDOC). *ACS Catal.* **2019**, *9* (8), 7023–7037. https://doi.org/10.1021/acscatal.9b01409.

(2) Zhao, Y.; Truhlar, D. G. A New Local Density Functional for Main-Group Thermochemistry, Transition Metal Bonding, Thermochemical Kinetics, and Noncovalent Interactions. *J. Chem. Phys.* **2006**, *125* (19), 194101. https://doi.org/10.1063/1.2370993.

(3) Zhao, Y.; Truhlar, D. G. The M06 Suite of Density Functionals for Main Group Thermochemistry, Thermochemical Kinetics, Noncovalent Interactions, Excited States, and Transition Elements: Two New Functionals and Systematic Testing of Four M06-Class Functionals and 12 Other Functionals. *Theor. Chem. Acc.* **2008**, *120* (1), 215–241. https://doi.org/10.1007/s00214-007-0310-x.

(4) Becke, A. D. Density-Functional Exchange-Energy Approximation with Correct Asymptotic Behavior. *Phys. Rev. A* **1988**, *38* (6), 3098–3100. https://doi.org/10.1103/PhysRevA.38.3098.

(5) Becke, A. D. Density-functional Thermochemistry. III. The Role of Exact Exchange. *J. Chem. Phys.* **1993**, *98* (7), 5648–5652. https://doi.org/10.1063/1.464913.

(6) Lee, C.; Yang, W.; Parr, R. G. Development of the Colle-Salvetti Correlation-Energy Formula into a Functional of the Electron Density. *Phys. Rev. B* **1988**, *37* (2), 785–789. https://doi.org/10.1103/PhysRevB.37.785.

(7) Grimme, S.; Ehrlich, S.; Goerigk, L. Effect of the Damping Function in Dispersion Corrected Density Functional Theory. *J. Comput. Chem.* **2011**, *32* (7), 1456–1465. https://doi.org/10.1002/jcc.21759.

(8) Staroverov, V. N.; Scuseria, G. E.; Tao, J.; Perdew, J. P. Comparative Assessment of a New Nonempirical Density Functional: Molecules and Hydrogen-Bonded Complexes. *J. Chem. Phys.* **2003**, *119* (23), 12129–12137. https://doi.org/10.1063/1.1626543.

(9) Tao, J.; Perdew, J. P.; Staroverov, V. N.; Scuseria, G. E. Climbing the Density Functional Ladder: Nonempirical Meta--Generalized Gradient Approximation Designed for Molecules and Solids. *Phys. Rev. Lett.* **2003**, *91* (14), 146401. https://doi.org/10.1103/PhysRevLett.91.146401.
