# Supplementary material for: Beyond Mimicking Enzymes: NewTAML/Peroxide Abstracts sp3 C–H Bonds to Initiate Biotranscendent Water-Purifying Mineralization of Fluoroquinolone Antibiotics
Source: J Am Chem Soc. 2025 Dec 23;148(1):292–307. doi: 10.1021/jacs.5c12768 (PMC12814164; doi:10.1021/jacs.5c12768)
Supplement: Supplementary file 2 [file ja5c12768_si_002.pdf]

Molecular Coordinates for Optimised Structures
===

================================
| Benchmarking NewTAML 3 |
================================

TPSSh_D3BJ_6-311+Gd
----

m=4, chrg=-1

Fe	0.16900	0.00000	0.10200
S	-2.49700	1.57400	0.04700
S	-2.49700	-1.57400	0.04700
C	1.29000	-2.56200	-0.28700
N	-0.92300	1.53100	-0.34800
N	-0.92300	-1.53200	-0.34800
N	1.56300	1.25600	-0.01000
N	1.56300	-1.25600	-0.01000
C	2.86100	-0.70800	0.08800
C	2.86100	0.70800	0.08800
C	5.26500	0.69700	0.27200
H	6.20200	1.24200	0.34500
C	5.26500	-0.69700	0.27200
H	6.20200	-1.24300	0.34500
C	4.06600	1.41000	0.17700
H	4.05100	2.49000	0.16500
C	4.06600	-1.41000	0.17700
H	4.05100	-2.49000	0.16500
C	-0.21700	2.84700	-0.43300
C	1.29000	2.56200	-0.28700
O	2.10800	3.47200	-0.42000
C	-0.21700	-2.84700	-0.43300
C	-0.45200	-3.48200	-1.81100
H	-1.50200	-3.75700	-1.92200
H	0.17900	-4.36900	-1.91400
H	-0.18800	-2.77100	-2.59900
C	-0.59000	-3.83300	0.69000
H	-0.45100	-3.37600	1.67300
H	0.06400	-4.70400	0.61300
H	-1.62900	-4.15600	0.59800
O	2.10800	-3.47200	-0.42100
C	-3.09600	0.00000	-0.62700
H	-4.16900	0.00000	-0.43000
H	-2.89500	0.00000	-1.69600

O	-3.26100	2.60600	-0.67900
O	-2.70000	1.55300	1.52000
O	-3.26100	-2.60600	-0.67900
O	-2.70000	-1.55300	1.52000
O	-0.50300	0.00000	2.16900
C	-0.59100	3.83300	0.68900
H	-1.62900	4.15600	0.59800
H	0.06400	4.70400	0.61300
H	-0.45100	3.37600	1.67300
C	-0.45100	3.48200	-1.81100
H	-0.18800	2.77100	-2.59900
H	0.17900	4.36900	-1.91400
H	-1.50100	3.75700	-1.92200
H	-1.12600	-0.75900	2.20400
H	-1.12600	0.75900	2.20400

M06_6-311+Gd_6-311+Gd

----

m=4, chrg=-1

Fe	5.14500	7.42900	13.07700
S	2.46900	8.02500	14.52400
S	2.47700	6.99000	11.56200
C	6.26800	6.98500	10.53200
N	4.04100	8.37100	14.36900
N	4.04900	7.35900	11.47400
N	6.53400	7.95800	14.22500
N	6.54000	7.12700	11.85600
C	7.83200	7.23100	12.40600
C	7.82900	7.69800	13.73600
C	10.22500	7.53900	13.78500
H	11.16300	7.65800	14.32500
C	10.22800	7.07900	12.47700
H	11.16800	6.83600	11.98500
C	9.02800	7.85300	14.42300
H	9.01300	8.22400	15.44100
C	9.03500	6.92500	11.77800
H	9.02400	6.57700	10.75200
C	4.75000	8.87900	15.57600
C	6.25500	8.67100	15.34800
O	7.06400	9.12200	16.14100
C	4.76400	7.00300	10.21800
C	4.52200	8.06000	9.14700
H	3.47200	8.06700	8.84400

H	5.15300	7.85600	8.27500
H	4.77800	9.05300	9.53200
C	4.42800	5.60900	9.69000
H	4.60100	4.84500	10.45700
H	5.07600	5.38800	8.83500
H	3.38600	5.53800	9.36300
O	7.08100	6.84300	9.63500
C	1.88300	8.13500	12.82200
H	0.80600	7.94700	12.88500
H	2.07000	9.15000	12.46700
O	1.71600	9.05400	15.23500
O	2.25300	6.64700	14.98700
O	1.72800	7.35100	10.36100
O	2.26000	5.62300	12.05700
O	4.48800	5.45400	13.76700
C	4.41100	8.11700	16.85700
H	3.36700	8.26000	17.15000
H	5.05200	8.48200	17.66600
H	4.59200	7.04200	16.73700
C	4.50400	10.37200	15.75200
H	4.76600	10.90800	14.83200
H	5.12900	10.75800	16.56300
H	3.45200	10.56500	15.97600
H	3.87900	5.14400	13.07000
H	3.87300	5.64700	14.50000

B3LYP_D3BJ_6-311+Gd

---

m=4, chrg=-1

Fe	5.15100	7.41300	13.08200
S	2.46800	7.98500	14.54700
S	2.47600	6.94100	11.56900
C	6.27600	6.96800	10.53000
N	4.04100	8.34400	14.38000
N	4.04800	7.32900	11.48200
N	6.54400	7.95500	14.23000
N	6.55000	7.12300	11.85300
C	7.84500	7.22100	12.40600
C	7.84200	7.68900	13.74200
C	10.24600	7.52400	13.79300
H	11.18100	7.64100	14.33300
C	10.24900	7.06400	12.48000
H	11.18700	6.82000	11.99000

C	9.04500	7.84200	14.43300
H	9.02900	8.21200	15.44700
C	9.05200	6.91200	11.77700
H	9.04100	6.56800	10.75400
C	4.75200	8.88700	15.57900
C	6.26300	8.65900	15.36000
O	7.07600	9.10100	16.16600
C	4.76500	7.00700	10.21000
C	4.52800	8.11900	9.17800
H	3.47900	8.14200	8.88400
H	5.15300	7.94700	8.30000
H	4.79100	9.08900	9.60700
C	4.40600	5.63100	9.62000
H	4.54700	4.83900	10.35900
H	5.06500	5.43300	8.77300
H	3.37200	5.60400	9.27700
O	7.09200	6.81100	9.62800
C	1.86100	8.08500	12.83900
H	0.79300	7.88000	12.90800
H	2.03800	9.09700	12.48400
O	1.70200	9.01400	15.27000
O	2.27100	6.59400	15.02300
O	1.71500	7.29200	10.35800
O	2.27800	5.55700	12.06500
O	4.48000	5.44100	13.77100
C	4.38800	8.17800	16.89600
H	3.35200	8.36900	17.17700
H	5.04200	8.55200	17.68500
H	4.53000	7.09900	16.81300
C	4.51100	10.39900	15.69200
H	4.77700	10.89000	14.75200
H	5.13300	10.81300	16.48700
H	3.46100	10.59900	15.90300
H	3.86800	5.13700	13.07200
H	3.86600	5.63800	14.50500

----

wB97XD_6-311+Gd

----

m=4, chrg=-1

Fe	5.15400	7.40500	13.08500
S	2.47300	7.98000	14.54000
S	2.48100	6.94300	11.57700
C	6.26800	6.97400	10.54000

N	4.03900	8.32900	14.38100
N	4.04700	7.31900	11.49100
N	6.53600	7.94800	14.22600
N	6.54200	7.12100	11.86100
C	7.83900	7.22100	12.40900
C	7.83500	7.68600	13.74100
C	10.23200	7.52100	13.79200
H	11.16700	7.63700	14.33200
C	10.23500	7.06300	12.48300
H	11.17300	6.81900	11.99200
C	9.03400	7.83700	14.43000
H	9.02000	8.20400	15.44600
C	9.04000	6.91100	11.78100
H	9.03200	6.56600	10.75800
C	4.74700	8.88300	15.56800
C	6.25600	8.65600	15.35000
O	7.06500	9.10200	16.15000
C	4.76000	7.01200	10.22100
C	4.52900	8.12600	9.19500
H	3.48100	8.15200	8.89500
H	5.15700	7.95600	8.31800
H	4.79200	9.09500	9.62800
C	4.40800	5.64100	9.62400
H	4.55200	4.84400	10.35800
H	5.06600	5.45000	8.77300
H	3.37300	5.61100	9.28000
O	7.08100	6.82400	9.64000
C	1.87300	8.07600	12.84200
H	0.80400	7.87200	12.91100
H	2.04500	9.09000	12.48800
O	1.71800	9.00900	15.25000
O	2.26900	6.60500	15.01700
O	1.73000	7.30300	10.37800
O	2.27800	5.57000	12.06200
O	4.49700	5.43300	13.77200
C	4.38900	8.18500	16.88900
H	3.35200	8.37600	17.17100
H	5.04300	8.56800	17.67500
H	4.53400	7.10500	16.81300
C	4.51300	10.39400	15.67200
H	4.78000	10.88100	14.73000
H	5.13700	10.81000	16.46600
H	3.46300	10.60000	15.88500
H	3.89200	5.12000	13.07900

H	3.89100	5.61900	14.50900

M06L_6-311+Gd

---

m=4, chrg=-1

Fe	5.14400	7.46700	13.06400
S	2.46600	8.05700	14.52000
S	2.47400	7.01300	11.54600
C	6.27500	6.95300	10.52200
N	4.04400	8.39300	14.37700
N	4.05100	7.36900	11.45400
N	6.54500	7.98400	14.22200
N	6.55100	7.15200	11.84100
C	7.84100	7.26100	12.39200
C	7.83800	7.72800	13.72900
C	10.24200	7.58300	13.77100
H	11.18000	7.70700	14.31000
C	10.24500	7.12500	12.45900
H	11.18600	6.88700	11.96500
C	9.04300	7.89000	14.41400
H	9.02700	8.25600	15.43300
C	9.05000	6.96300	11.76100
H	9.03900	6.61400	10.73600
C	4.75300	8.86100	15.60400
C	6.26300	8.65100	15.37700
O	7.07100	9.06600	16.20000
C	4.76600	6.97100	10.20600
C	4.53000	7.99900	9.10100
H	3.48700	7.99400	8.78200
H	5.17300	7.77400	8.24600
H	4.77500	9.00600	9.45400
C	4.40400	5.56700	9.71500
H	4.55000	4.81900	10.50000
H	5.05000	5.30400	8.87200
H	3.36300	5.51200	9.38400
O	7.08800	6.76600	9.62300
C	1.90600	8.18300	12.80400
H	0.82400	8.03300	12.85400
H	2.12100	9.19100	12.45100
O	1.71400	9.08500	15.24200
O	2.23300	6.67000	14.96900
O	1.72700	7.36400	10.33600
O	2.24100	5.65000	12.06000

O	4.46600	5.47600	13.76000
C	4.38500	8.07000	16.86300
H	3.34200	8.23100	17.14900
H	5.02500	8.39200	17.68900
H	4.53400	6.99500	16.71700
C	4.51500	10.35400	15.82500
H	4.76300	10.92100	14.92300
H	5.15300	10.71200	16.63800
H	3.47000	10.54800	16.07300
H	3.84700	5.19600	13.05900
H	3.84400	5.69400	14.47900

================================

| Hypothetical Acitve Catalysts |

================================

[2=0] 2e-ox : M06L_6-311+Gd_smd

---

m=2, chrg=-1

C	10.20100	7.24200	12.41600
C	9.02000	7.08800	11.70400
C	7.81900	7.30000	12.37800
C	7.82900	7.68200	13.75300
C	9.04700	7.82700	14.43400
C	10.23300	7.60200	13.76600
N	6.53700	7.21400	11.85700
Fe	5.13400	7.32900	13.10800
N	4.04900	7.44000	11.47700
S	2.45200	7.13400	11.54700
O	1.79200	7.46800	10.27700
N	6.56400	7.90700	14.24000
C	6.26900	8.57100	15.39700
C	4.77600	8.81600	15.58800
C	4.34400	7.98000	16.79500
C	6.23500	7.02100	10.53100
C	4.74300	6.97500	10.23600
C	4.48900	7.91600	9.05900
O	7.07100	6.86200	9.65100
O	7.09100	8.96200	16.21700
C	4.41900	5.52600	9.86000
O	4.90400	5.82000	13.59800
N	4.10100	8.42100	14.30400
S	2.47500	8.18600	14.42300

O	1.85700	9.27700	15.18600
C	4.60300	10.31000	15.85800
O	2.11200	5.81000	12.08800
C	1.87600	8.39800	12.72200
C	0.36000	8.39000	12.74800
O	2.13200	6.83100	14.87200
H	11.18200	7.69100	14.28200
N	11.45600	7.00000	11.72700
H	9.05400	8.09600	15.48100
H	9.03700	6.79200	10.66700
H	3.46600	7.83300	8.69800
H	5.16300	7.65700	8.23900
H	4.67700	8.95600	9.33900
H	4.59600	4.84500	10.69700
H	5.04800	5.21300	9.02300
H	3.37800	5.42500	9.54400
H	2.28300	9.35000	12.37100
H	3.30700	8.18000	17.07200
H	4.96800	8.24200	17.65300
H	4.45600	6.90900	16.60400
H	4.89500	10.90200	14.98700
H	5.24000	10.60000	16.69600
H	3.57300	10.55100	16.11300
H	-0.02500	8.56900	11.74400
H	-0.03700	7.43800	13.10800
H	-0.00700	9.18800	13.39300
O	12.50700	7.16400	12.35000
O	11.42200	6.64600	10.54700

[2-OH] 2e-ox : M06L_6-311+Gd_smd

----

m=2, chrg=0

C	10.19000	7.30400	12.36300
C	9.02800	7.02800	11.68900
C	7.81600	7.28100	12.35900
C	7.82800	7.83900	13.69500
C	9.05700	8.09900	14.33500
C	10.22600	7.82700	13.67300
N	6.56100	7.10200	11.87300
Fe	5.13100	7.46600	13.07700
N	4.08500	7.39100	11.48000
S	2.43500	7.24300	11.48500
O	1.87900	7.72200	10.21900

N	6.57800	8.07500	14.16800
C	6.26200	8.65400	15.38900
C	4.75900	8.79000	15.61900
C	4.40100	7.85400	16.77500
C	6.23500	6.64900	10.60000
C	4.76100	6.84100	10.25500
C	4.72600	7.87200	9.12200
O	7.02400	6.16200	9.81600
O	7.07400	9.01600	16.21800
C	4.21300	5.49300	9.79200
O	4.91600	5.82300	13.73000
N	4.08200	8.42000	14.32900
S	2.46200	8.10800	14.42700
O	1.77700	9.07500	15.28000
C	4.48500	10.25000	15.97800
O	2.01100	5.91100	11.91900
C	1.90700	8.46200	12.73000
C	0.39200	8.52600	12.71400
O	2.23600	6.68900	14.73100
H	11.18400	8.01400	14.14500
N	11.46300	7.04100	11.68300
H	9.07600	8.50600	15.33600
H	9.04300	6.62600	10.68700
H	3.71000	8.01100	8.75500
H	5.34500	7.51800	8.29400
H	5.11400	8.83800	9.45500
H	4.22000	4.75700	10.59900
H	4.83700	5.11900	8.97800
H	3.19600	5.58600	9.40700
H	2.36900	9.41300	12.44900
H	3.35500	7.96900	17.06800
H	5.01300	8.10500	17.64500
H	4.58200	6.80800	16.51600
H	4.71600	10.91200	15.14000
H	5.11600	10.53300	16.82300
H	3.44600	10.39700	16.26500
H	0.04300	8.76800	11.70900
H	-0.06400	7.58200	13.02300
H	0.05100	9.31500	13.38400
O	12.49800	7.28400	12.29500
O	11.42900	6.59500	10.54100
H	4.08100	5.78800	14.23500

[2=0] 1e_ox : M06L_6-311+Gd_smd

----

m=3, chrg=-2

C	10.22100	7.17800	12.43900
C	9.03300	7.02900	11.72000
C	7.83400	7.33400	12.34900
C	7.83600	7.78900	13.70200
C	9.05100	7.92000	14.39300
C	10.24300	7.61500	13.76300
N	6.55300	7.25800	11.80100
Fe	5.13600	7.41800	13.07300
N	4.04300	7.45500	11.44100
S	2.46000	7.15200	11.52800
O	1.76700	7.50200	10.27200
N	6.56700	8.05500	14.18400
C	6.26800	8.66900	15.35500
C	4.76300	8.84500	15.59800
C	4.40600	7.95100	16.78800
C	6.25800	6.99700	10.51200
C	4.75300	6.97700	10.21000
C	4.51700	7.92800	9.03800
O	7.07500	6.79000	9.60500
O	7.08300	9.08000	16.18600
C	4.39900	5.54000	9.82300
O	5.00000	5.86300	13.61300
N	4.05100	8.47200	14.33200
S	2.46700	8.17900	14.45800
O	1.77400	9.23500	15.22300
C	4.53200	10.31700	15.94000
O	2.10800	5.81400	12.04300
C	1.87200	8.38500	12.74200
C	0.35700	8.34700	12.76000
O	2.11600	6.81200	14.89000
H	11.18500	7.71000	14.29100
N	11.46600	6.86400	11.78600
H	9.05200	8.25500	15.42100
H	9.05100	6.68500	10.69700
H	3.48300	7.89000	8.69900
H	5.16100	7.64800	8.20000
H	4.75200	8.95900	9.31600
H	4.55100	4.85000	10.65800
H	5.03100	5.21400	8.99200
H	3.36000	5.46000	9.49400
H	2.25800	9.35000	12.40300

H	3.36700	8.09500	17.09600
H	5.03800	8.20300	17.64300
H	4.55300	6.89300	16.55300
H	4.77600	10.96000	15.09000
H	5.17100	10.60600	16.77900
H	3.49700	10.49900	16.22300
H	-0.03400	8.53300	11.75800
H	-0.02200	7.38200	13.10500
H	-0.03000	9.12600	13.41800
O	12.52300	6.99700	12.41700
O	11.44200	6.47200	10.61200

[2-OH] 1e_ox : M06L_6-311+Gd_smd

m=3, chrg=-1

C	10.24100	7.12800	12.44900
C	9.05800	6.96800	11.73100
C	7.86800	7.33400	12.34700
C	7.87700	7.85200	13.67300
C	9.08900	7.98800	14.36700
C	10.27200	7.62700	13.75100
N	6.58200	7.25300	11.81400
Fe	5.18200	7.49800	13.05200
N	4.08300	7.47200	11.45500
S	2.48700	7.14000	11.53400
O	1.80800	7.51900	10.28700
N	6.60800	8.15700	14.12900
C	6.30700	8.81000	15.29000
C	4.80400	8.89500	15.56600
C	4.53200	7.96400	16.75100
C	6.28300	7.00100	10.50900
C	4.78300	7.01000	10.21200
C	4.56000	8.00500	9.07100
O	7.10300	6.79700	9.61800
O	7.12300	9.29300	16.06800
C	4.40900	5.59100	9.78100
O	4.95200	5.84500	13.69800
N	4.09800	8.48200	14.30700
S	2.52600	8.07100	14.46500
O	1.79400	9.01400	15.31700
C	4.47600	10.34400	15.91700
O	2.18900	5.78300	12.01400
C	1.87800	8.32300	12.78200

C	0.36600	8.23100	12.82700
O	2.35900	6.64900	14.82200
H	11.21700	7.72500	14.27300
N	11.48700	6.75100	11.81400
H	9.09700	8.37000	15.37800
H	9.07300	6.56600	10.73000
H	3.52500	7.99200	8.73400
H	5.20100	7.73700	8.22800
H	4.81300	9.02200	9.38200
H	4.55300	4.87300	10.59300
H	5.04000	5.29000	8.94100
H	3.37000	5.53500	9.44500
H	2.22700	9.31100	12.46700
H	3.49300	8.03200	17.08100
H	5.16400	8.25400	17.59400
H	4.75200	6.92300	16.50000
H	4.66000	11.00600	15.06600
H	5.11100	10.66900	16.74400
H	3.43600	10.45100	16.22200
H	-0.04600	8.41600	11.83500
H	0.02500	7.25100	13.16800
H	-0.03200	8.99100	13.50000
O	12.53600	6.87400	12.45300
O	11.45600	6.32200	10.65700
H	4.10200	5.80900	14.17800

Transition States

[2-OH] 2e_ox + piperazineMe2_H+ : M06L_6-311+Gd_smd

m=2, chrg=1

N	1.25600	1.60300	0.01300
N	-1.21300	2.20400	-0.11100
N	1.00100	-0.36000	1.51500
N	-1.53700	-0.24100	1.68400
N	6.14900	1.04800	-0.11300
C	4.82500	0.66300	0.34500
C	3.73400	1.39100	-0.11000
C	2.47000	1.01300	0.33400
C	2.32100	-0.10400	1.21100
C	3.45300	-0.81100	1.64900

C	4.70400	-0.42500	1.21500
C	0.54900	-1.23200	2.47300
C	-0.97500	-1.37900	2.48600
C	-1.27300	-2.75400	1.88100
C	1.10000	2.81000	-0.61900
C	-0.36200	3.18800	-0.85800
C	-0.56200	4.59900	-0.30400
C	-0.57300	3.16300	-2.37500
C	-1.44400	-1.30100	3.93600
C	-3.58100	1.31400	0.85500
C	-5.07900	1.34000	0.62700
O	-3.37900	3.45000	-0.65900
O	2.01500	3.54800	-0.96200
O	1.25700	-1.85100	3.25700
O	-0.55000	-0.47600	-1.00200
O	-3.96300	-0.90600	2.22100
O	-2.99400	1.26100	-1.75300
O	-3.15000	-1.15500	-0.10800
O	7.11900	0.40400	0.29200
O	6.25000	2.00000	-0.88800
Fe	-0.27000	0.60900	0.48500
S	-2.77300	2.11400	-0.57500
S	-3.07200	-0.40800	1.16800
H	5.58900	-0.96100	1.53700
H	3.34300	-1.66000	2.30900
H	3.86900	2.22100	-0.78800
H	-1.55900	4.97200	-0.53400
H	0.17000	5.27300	-0.75500
H	-0.42300	4.61800	0.78000
H	-0.44700	2.15600	-2.78200
H	0.15700	3.82100	-2.85200
H	-1.56700	3.52700	-2.64400
H	-3.28200	1.88500	1.73800
H	-2.33800	-2.99000	1.93200
H	-0.73700	-3.52600	2.44100
H	-0.95400	-2.80500	0.83600
H	-1.24300	-0.31500	4.36200
H	-0.90700	-2.04500	4.53000
H	-2.51000	-1.50400	4.01600
H	-5.41300	2.36700	0.47900
H	-5.37100	0.75000	-0.24500
H	-5.59400	0.94500	1.50300
H	-1.43300	-0.89900	-0.93900
N	-0.85000	-3.22600	-3.31000

N	1.14000	-3.43600	-1.19500
C	-1.10800	-3.91200	-1.99700
C	0.45600	-2.44900	-3.30800
C	0.15800	-4.48500	-1.42000
C	1.03000	-2.28800	-1.93100
C	2.00100	-3.53100	-0.04300
C	-2.01500	-2.38700	-3.70600
H	-1.54400	-3.16500	-1.33100
H	-1.84800	-4.69000	-2.17800
H	1.14600	-3.00300	-3.95100
H	0.27300	-1.47700	-3.76300
H	-0.06600	-4.97600	-0.46900
H	0.59300	-5.24800	-2.08000
H	1.92600	-1.66200	-1.90100
H	0.20900	-1.39000	-1.31000
H	2.44800	-4.52900	-0.01000
H	1.42700	-3.39400	0.88100
H	2.78900	-2.78200	-0.10700
H	-0.75000	-3.96000	-4.01300
H	-2.91100	-3.00600	-3.69300
H	-2.11300	-1.56600	-2.99600
H	-1.83800	-2.00000	-4.70800

[2-OH] 2e_ox + piperazineMe2_2H+ : M06L_6-311+Gd_smd

----

m=2, chrg=2

N	2.02500	-0.94800	-0.37700
N	-0.31200	-0.97700	-1.32100
N	2.00000	1.40600	0.38900
N	-0.26800	1.97000	-0.56400
N	6.80700	-1.43300	0.73400
C	5.53200	-0.66900	0.67600
C	4.42200	-1.30600	0.18400
C	3.23500	-0.55300	0.10900
C	3.22100	0.82800	0.56100
C	4.39800	1.42800	1.05700
C	5.54700	0.67800	1.10500
C	1.68800	2.75100	0.65900
C	0.42200	3.20900	-0.07600
C	-0.42900	4.05100	0.86900
C	1.74000	-2.21900	-0.91400
C	0.34000	-2.31300	-1.53000
C	0.53200	-2.63000	-3.01500

C	-0.40000	-3.43900	-0.80700
C	0.92900	4.04900	-1.26300
C	-2.04300	0.84800	-2.32700
C	-3.39900	1.02100	-2.98400
O	-2.35900	-1.75600	-2.68300
O	2.50300	-3.15100	-0.89200
O	2.34800	3.48300	1.35100
O	-0.13700	-0.24700	1.61400
O	-1.99000	3.42400	-1.82700
O	-2.68800	-0.86600	-0.38900
O	-2.76100	1.93800	0.01600
O	7.82500	-0.76700	0.80500
O	6.72700	-2.64600	0.69400
Fe	0.61300	0.30100	-0.26600
S	-1.90600	-0.84200	-1.65200
S	-1.83100	2.18100	-1.10000
H	6.48100	1.09600	1.46900
H	4.38400	2.45500	1.39900
H	4.46800	-2.34300	-0.12300
H	-0.42200	-2.81300	-3.50600
H	1.15100	-3.52400	-3.11700
H	1.03700	-1.80900	-3.53200
H	-0.52700	-3.21000	0.25700
H	0.17300	-4.36600	-0.88400
H	-1.38000	-3.61500	-1.25700
H	-1.21700	0.95800	-3.03700
H	-1.26600	4.52100	0.35000
H	0.19200	4.84400	1.29000
H	-0.81800	3.45400	1.69800
H	1.53100	3.44700	-1.94900
H	1.55000	4.86400	-0.88500
H	0.09300	4.47500	-1.81700
H	-3.52900	0.26900	-3.76300
H	-4.21400	0.91700	-2.26300
H	-3.46900	2.00800	-3.44200
H	0.49700	-0.29200	2.34100
N	-4.68600	-2.37000	2.05500
N	-3.32600	0.24300	2.10200
C	-5.23200	-1.16700	1.33700
C	-3.14600	-2.25000	2.12900
C	-4.82400	0.11200	2.02600
C	-2.69000	-0.95900	2.68300
C	-2.95200	1.47000	2.86400
C	-5.10200	-3.64300	1.39500

H	-4.84300	-1.21600	0.31700
H	-6.32100	-1.25200	1.30500
H	-2.79500	-3.08200	2.74300
H	-2.83500	-2.40700	1.09400
H	-5.20900	0.97200	1.47100
H	-5.20400	0.17400	3.05200
H	-2.62300	-0.86800	3.76700
H	-0.99500	-0.66100	1.94400
H	-3.31500	1.37700	3.88800
H	-3.38800	2.33800	2.37600
H	-1.86600	1.55600	2.85700
H	-2.99300	0.38100	1.12600
H	-5.05000	-2.36900	3.01300
H	-6.19000	-3.70500	1.37800
H	-4.71400	-3.64400	0.37600
H	-4.69000	-4.48400	1.95100

[2-OH] 1e_ox + piperazineMe2_H+ : M06L_6-311+Gd_smd

----

m=3, chrg=0

N	6.98300	2.18200	13.08000
N	7.66500	3.47400	15.43400
C	5.87300	2.63300	13.97700
C	8.05800	3.23300	13.01200
C	6.38700	2.76800	15.38600
C	8.11500	4.07000	14.26300
C	7.92600	4.16200	16.68000
C	6.49400	1.81500	11.72500
H	5.50600	3.57500	13.56400
H	5.07300	1.89500	13.92100
H	8.99800	2.70600	12.82100
H	7.84500	3.85800	12.14200
H	5.64400	3.31900	15.97700
H	6.50500	1.78800	15.86300
H	9.08100	4.56100	14.39400
H	7.32400	5.14600	14.05100
H	7.23500	5.00700	16.80800
H	8.95000	4.53400	16.69600
H	7.78000	3.47300	17.51500
H	7.39600	1.34300	13.49800
H	5.81400	0.96900	11.81700
H	5.97500	2.67500	11.30300
H	7.34800	1.54800	11.10500

N	7.14400	8.09500	11.74900
N	4.81400	7.06000	11.56900
N	6.83200	9.08200	14.05700
N	4.50500	8.11500	14.48100
N	11.53400	10.29300	11.28600
C	10.33100	10.00700	12.02900
C	9.37000	9.17500	11.45000
C	8.21500	8.89900	12.16400
C	8.03000	9.45700	13.46400
C	9.01800	10.28900	14.01100
C	10.16900	10.56500	13.29500
C	6.36400	9.50100	15.26400
C	5.02400	8.86700	15.66900
C	5.31400	7.96400	16.87000
C	6.94100	7.63400	10.49300
C	5.60600	6.89700	10.30300
C	4.89700	7.55100	9.11800
C	5.94400	5.43900	9.99300
C	4.07500	9.99800	16.05700
C	2.56800	6.88400	13.08000
C	1.27000	6.11600	13.22900
O	2.67600	6.04000	10.59300
O	7.71700	7.76300	9.54100
O	6.92200	10.29800	16.01900
O	6.50500	6.05700	13.94000
O	2.29000	7.50200	15.63100
O	3.91500	4.72100	12.27100
O	3.87300	5.68100	15.09400
O	12.38700	11.03100	11.79400
O	11.67400	9.79100	10.16400
Fe	5.82700	7.77900	13.08300
S	3.56000	6.06600	11.77500
S	3.34900	7.02100	14.72900
H	10.93700	11.20600	13.71200
H	8.88100	10.71200	14.99600
H	9.53200	8.75400	10.46900
H	3.98700	7.01200	8.85500
H	5.55600	7.55000	8.24700
H	4.63100	8.58800	9.34200
H	6.42800	4.95600	10.84800
H	6.62400	5.38800	9.13800
H	5.05000	4.86500	9.73500
H	2.40500	7.91000	12.73900
H	4.39300	7.53900	17.27800

H	5.78700	8.54400	17.66700
H	5.98500	7.14300	16.60000
H	3.86100	10.64300	15.20000
H	4.52800	10.61100	16.83900
H	3.13100	9.60600	16.43600
H	0.76100	6.05100	12.26600
H	1.43600	5.10200	13.60000
H	0.60700	6.63400	13.92200
H	5.76200	5.70900	14.46900

[2=O] 2e_ox + piperazineMe2_2H+ : M06L_6-311+Gd_smd

----

m=2, chrg=1

N	1.20600	-0.27900	0.91700
N	-1.10200	0.62300	1.36400
N	0.44700	-2.39100	-0.13300
N	-2.02800	-1.86300	-0.00300
N	5.93800	-1.43300	0.16500
C	4.52100	-1.72900	0.09000
C	3.62100	-0.78100	0.57300
C	2.26800	-1.07600	0.49200
C	1.82900	-2.29700	-0.08800
C	2.76400	-3.22600	-0.56300
C	4.11300	-2.93900	-0.47000
C	-0.27800	-3.48900	-0.49600
C	-1.78200	-3.22300	-0.58600
C	-2.14300	-3.32000	-2.07100
C	1.28900	0.75400	1.79800
C	-0.07600	1.31200	2.20900
C	-0.27500	0.89500	3.67000
C	-0.05200	2.83600	2.10000
C	-2.49200	-4.30100	0.22900
C	-3.72300	0.02100	0.98000
C	-5.10600	0.62300	0.82400
O	-2.94400	2.10200	2.34300
O	2.33000	1.17900	2.29600
O	0.18900	-4.59700	-0.73500
O	-0.40600	-0.08900	-1.42700
O	-4.56000	-2.12300	-0.26800
O	-2.53600	2.16800	-0.10900
O	-3.41600	-0.39800	-1.63800
O	6.73900	-2.27400	-0.25100
O	6.29100	-0.35100	0.64100

Fe	-0.46100	-0.75500	0.16500
S	-2.51800	1.37900	1.13900
S	-3.45800	-1.16000	-0.38100
H	4.85200	-3.64200	-0.83800
H	2.43400	-4.15500	-1.00700
H	3.97100	0.15100	0.99200
H	-1.24500	1.24100	4.03200
H	0.50400	1.34100	4.29400
H	-0.22900	-0.19200	3.78200
H	-0.16500	3.17900	1.06900
H	0.90200	3.21000	2.47900
H	-0.84600	3.29100	2.69200
H	-3.62400	-0.55700	1.90400
H	-3.22200	-3.24000	-2.22400
H	-1.82800	-4.29300	-2.45700
H	-1.64800	-2.54200	-2.65800
H	-2.24600	-4.21700	1.29000
H	-2.17100	-5.28600	-0.11800
H	-3.57300	-4.23500	0.11500
H	-5.31600	1.29300	1.65800
H	-5.20400	1.18400	-0.10800
H	-5.85800	-0.16700	0.83600
N	2.27900	3.83000	-1.25300
N	-0.44400	3.03100	-2.00500
C	1.12500	4.78300	-1.18600
C	1.77500	2.42000	-1.05900
C	0.07600	4.43700	-2.20300
C	0.69600	2.09700	-2.02600
C	-1.46500	2.68800	-3.03600
C	3.32400	4.15400	-0.24500
H	0.74500	4.74300	-0.16200
H	1.50700	5.78700	-1.37500
H	2.61800	1.73800	-1.17400
H	1.42700	2.38700	-0.02700
H	-0.77600	5.11300	-2.11900
H	0.46000	4.47900	-3.22500
H	0.98800	1.87500	-3.05400
H	0.16100	0.86000	-1.64500
H	-1.00300	2.76900	-4.01800
H	-2.29000	3.39200	-2.94400
H	-1.81100	1.67100	-2.85800
H	-0.91600	2.96700	-1.08600
H	2.69900	3.89200	-2.18700
H	3.69100	5.16100	-0.43800

H	2.87300	4.09700	0.74600
H	4.13200	3.43000	-0.33500

[2=0] 2e_ox + piperazineMe2_H+ : M06L_6-311+Gd_smd

----

m=2, chrg=0

N	1.01700	0.41100	1.28200
N	-1.45200	0.96500	1.46500
N	0.70800	-1.89500	0.35600
N	-1.82700	-1.80400	0.20700
N	5.91300	-0.16000	1.39700
C	4.58100	-0.65000	1.13000
C	3.50000	0.20400	1.35400
C	2.22100	-0.27000	1.09700
C	2.03800	-1.59200	0.59600
C	3.14900	-2.42400	0.39500
C	4.42100	-1.95300	0.66200
C	0.23500	-3.06000	-0.16400
C	-1.28100	-3.06500	-0.38900
C	-1.49700	-3.16300	-1.90200
C	0.87600	1.67400	1.74800
C	-0.58500	2.09100	1.95300
C	-0.75700	2.28800	3.46100
C	-0.81100	3.40500	1.21000
C	-1.83800	-4.29900	0.32000
C	-3.83700	-0.27100	1.11700
C	-5.32900	-0.02700	1.00900
O	-3.60500	2.05500	2.31000
O	1.79500	2.44200	2.04800
O	0.91100	-4.04800	-0.45100
O	-0.34700	0.24800	-1.05700
O	-4.25700	-2.52300	-0.17400
O	-3.22400	2.01000	-0.13300
O	-3.39200	-0.62400	-1.50300
O	6.87400	-0.91300	1.20100
O	6.04900	0.99700	1.81100
Fe	-0.49400	-0.41400	0.49000
S	-3.00100	1.35000	1.16500
S	-3.32800	-1.38100	-0.24000
H	5.28700	-2.58500	0.50400
H	3.01100	-3.43000	0.02400
H	3.66000	1.20600	1.72400
H	-1.75600	2.65400	3.69800

H	-0.03000	3.02000	3.82300
H	-0.59500	1.35100	4.00100
H	-0.76200	3.26500	0.12700
H	-0.04300	4.12700	1.50100
H	-1.78200	3.84300	1.45500
H	-3.56700	-0.78100	2.04500
H	-2.55700	-3.27000	-2.14600
H	-0.98100	-4.04400	-2.29100
H	-1.10900	-2.28100	-2.42100
H	-1.70300	-4.22300	1.40300
H	-1.31100	-5.19000	-0.02700
H	-2.90000	-4.42800	0.11300
H	-5.66400	0.60700	1.83100
H	-5.59600	0.45700	0.06700
H	-5.86600	-0.97400	1.07700
N	0.13500	2.81400	-3.28400
N	2.28200	0.88000	-2.81900
C	0.27700	1.52300	-4.05200
C	1.19500	2.94500	-2.20600
C	1.71900	1.09600	-4.14700
C	1.72200	1.60600	-1.78700
C	2.94000	-0.37200	-2.53700
C	-1.24800	2.95000	-2.74900
H	-0.31500	0.77500	-3.51700
H	-0.16200	1.68100	-5.03600
H	1.99400	3.56100	-2.62900
H	0.76100	3.48100	-1.36400
H	1.78300	0.17000	-4.72000
H	2.31700	1.84800	-4.67900
H	2.35400	1.64100	-0.89500
H	0.70700	0.91600	-1.33300
H	3.62300	-0.62000	-3.35100
H	2.20500	-1.18500	-2.44500
H	3.50000	-0.28900	-1.60400
H	0.29300	3.57800	-3.94300
H	-1.94900	2.85000	-3.57700
H	-1.40700	2.15800	-2.02000
H	-1.35000	3.93000	-2.28600

[2=0] 1e_ox + piperazineMe2_H+ : M06L_6-311+Gd_smd

---

m=3, chrg=-1

N	3.81100	-2.46600	1.47500

N	0.90200	-2.70200	1.90200
C	2.98500	-3.59000	0.96600
C	2.70400	-1.15300	1.66200
C	1.78500	-3.84600	1.85800
C	1.55000	-1.52400	2.40800
C	-0.35900	-2.98600	2.55500
C	4.90900	-2.10700	0.57000
H	2.65800	-3.28800	-0.03600
H	3.61800	-4.48200	0.87100
H	3.32500	-0.36300	2.08900
H	2.55400	-1.00100	0.59500
H	1.24400	-4.70900	1.44600
H	2.12900	-4.14900	2.87500
H	1.63600	-1.48400	3.50900
H	0.41700	-0.40000	1.82200
H	-0.23200	-3.20400	3.63400
H	-0.84500	-3.84600	2.08500
H	-1.02600	-2.12700	2.44500
H	4.15500	-2.69000	2.41000
H	5.59100	-2.95000	0.41800
H	4.47400	-1.82100	-0.38900
H	5.45900	-1.25400	0.97000
N	0.92000	0.14000	-0.92800
N	-1.30700	-1.02300	-1.13600
N	-0.00700	2.28900	-0.06100
N	-2.41400	1.50800	0.05900
N	5.52400	1.91800	-0.79600
C	4.09100	2.07300	-0.64700
C	3.26500	0.98700	-0.94200
C	1.89900	1.10900	-0.72200
C	1.35900	2.33600	-0.23100
C	2.21800	3.41900	0.02700
C	3.58100	3.28300	-0.17500
C	-0.79400	3.31200	0.39300
C	-2.25900	2.89400	0.59300
C	-2.54200	2.98500	2.09600
C	1.11600	-1.10300	-1.43100
C	-0.20400	-1.80500	-1.77400
C	-0.31900	-1.71600	-3.30400
C	-0.12300	-3.26500	-1.33400
C	-3.11600	3.89300	-0.18800
C	-3.95200	-0.48300	-0.87200
C	-5.32000	-1.13000	-0.76800
O	-3.06400	-2.52200	-2.22600

O	2.20800	-1.63000	-1.66800
O	-0.42300	4.45400	0.62800
O	-0.45400	0.08100	1.53100
O	-4.95200	1.60000	0.35000
O	-2.80600	-2.63000	0.24500
O	-3.68300	-0.02100	1.75100
O	6.24300	2.90800	-0.63600
O	5.96600	0.79400	-1.06200
Fe	-0.76700	0.53400	-0.11800
S	-2.72300	-1.82800	-0.97300
S	-3.78700	0.71600	0.49700
H	4.26400	4.09800	0.04000
H	1.79600	4.34500	0.39600
H	3.68200	0.07100	-1.33400
H	-1.22500	-2.21900	-3.64300
H	0.55200	-2.18500	-3.77200
H	-0.36400	-0.67100	-3.62800
H	-0.10100	-3.33600	-0.24500
H	0.79500	-3.70400	-1.73500
H	-0.97200	-3.83900	-1.71000
H	-3.82800	0.09700	-1.79100
H	-3.60400	2.82500	2.30200
H	-2.26600	3.97900	2.46100
H	-1.97100	2.23200	2.64500
H	-2.94600	3.78800	-1.26400
H	-2.83600	4.90900	0.10400
H	-4.17600	3.73500	0.00600
H	-5.46900	-1.82100	-1.59900
H	-5.41100	-1.68700	0.16700
H	-6.09500	-0.36300	-0.79400

[2=0] 1e_ox + piperazineMe2_2H+ : M06L_6-311+Gd_smd

----

m=3, chrg=0

N	-3.32900	-2.54700	2.28800
N	-2.12500	0.03700	2.88400
C	-4.09200	-1.26200	2.16500
C	-1.82000	-2.23100	2.04200
C	-3.56700	-0.23000	3.13500
C	-1.26600	-1.19300	2.92300
C	-1.59600	1.08300	3.78700
C	-3.83700	-3.59300	1.36000
H	-3.96700	-0.93200	1.12800

H	-5.14900	-1.47000	2.35100
H	-1.29000	-3.18000	2.15700
H	-1.81500	-1.94000	0.98600
H	-4.12500	0.70100	2.99800
H	-3.66800	-0.54400	4.18200
H	-1.12800	-1.48200	3.97100
H	-0.21900	-0.59500	2.05800
H	-1.67400	0.74100	4.82200
H	-2.16900	2.00100	3.64900
H	-0.55400	1.24800	3.51900
H	-2.05500	0.43400	1.91800
H	-3.41200	-2.88300	3.25100
H	-4.88900	-3.79000	1.57300
H	-3.71400	-3.22000	0.34300
H	-3.25100	-4.50100	1.50300
N	1.77000	-0.84000	-0.59900
N	-0.64400	-0.96900	-1.36100
N	1.78800	1.61200	-0.24900
N	-0.67400	1.99000	-0.71200
N	6.53400	-1.22100	0.68000
C	5.31300	-0.44600	0.43500
C	4.16600	-1.12500	0.03900
C	3.01300	-0.37600	-0.17800
C	3.02500	1.03400	0.00500
C	4.20200	1.68200	0.40300
C	5.34800	0.93400	0.61600
C	1.49200	2.94600	-0.13400
C	-0.00800	3.25300	-0.27900
C	-0.48700	3.72600	1.09600
C	1.49100	-2.11200	-1.01600
C	0.12900	-2.20400	-1.72000
C	0.47200	-2.22500	-3.21800
C	-0.55700	-3.50500	-1.31600
C	-0.15800	4.35300	-1.32700
C	-2.65100	0.77800	-2.02100
C	-4.13100	0.84600	-2.34100
O	-2.45600	-1.60800	-3.07300
O	2.24200	-3.07200	-0.96200
O	2.29600	3.84000	0.07000
O	0.08700	0.24800	1.37500
O	-2.93400	3.17500	-0.95900
O	-3.08200	-1.47400	-0.68700
O	-2.80800	1.24800	0.58500
O	7.54300	-0.60700	1.01800

O	6.48000	-2.43800	0.53900
Fe	0.36400	0.37900	-0.33000
S	-2.20700	-0.97500	-1.77800
S	-2.27800	1.90900	-0.64200
H	6.27500	1.40400	0.92300
H	4.20400	2.75600	0.53000
H	4.17600	-2.19800	-0.09500
H	-0.42000	-2.36700	-3.82500
H	1.17400	-3.03900	-3.41800
H	0.94700	-1.28500	-3.51500
H	-0.81500	-3.51000	-0.25300
H	0.12900	-4.33700	-1.49000
H	-1.46700	-3.67700	-1.89600
H	-2.02600	1.11900	-2.85200
H	-1.53400	4.04000	1.06000
H	0.11500	4.57600	1.42700
H	-0.38100	2.92100	1.82900
H	0.13800	3.98900	-2.31500
H	0.49500	5.18900	-1.06600
H	-1.18800	4.70600	-1.38500
H	-4.36300	0.18800	-3.18000
H	-4.73200	0.53600	-1.48300
H	-4.40600	1.86800	-2.60400
